# Hepatic retromer is essential for systemic cholesterol homeostasis by regulating lysosomal cholesterol handling

**DOI:** 10.1016/j.molmet.2026.102456

**Published:** 2026-10-11

**Authors:** Cristy R.C. Verzijl, Markus G. Barbosa, Dyonne Y. Vos, Andries H. Heida, Amandine Caillaud, Lauri Vanharanta, Xiaoke Ge, Joël J. Tissink, Mirjam H. Koster, Marieke Smit, Nicolette Huijkman, Niels J. Kloosterhuis, Aurore Girardeau, Zoé Begué-Racapé, Vincent W. Bloks, Rick Havinga, Marceline M. Fuh, Fulvio Reggiori, Muriel Mari, Ludger Scheja, Folkert Kuipers, Santiago Solé-Domènech, Sander Kooijman, Joerg Heeren, Jan Freark de Boer, Patrick C.N. Rensen, Elina Ikonen, Antoine Rimbert, Justina C. Wolters, Jan Albert Kuivenhoven, Bart van de Sluis

**Affiliations:** 1 Department of Pediatrics, University of Groningen, University Medical Center Groningen, Groningen, The Netherlands; 2 Nantes Université, CHU Nantes, CNRS, INSERM, l'institut du thorax, Nantes, F-44000, France; 3 Department of Anatomy and Stem Cells and Metabolism Research Program, University of Helsinki, Helsinki, Finland; 4 Minerva Foundation Institute for Medical Research, Helsinki, Finland; 5 Department of Medicine, Division of Endocrinology, Leiden University Medical Center, Albinusdreef 2, Leiden, 2333 ZA, The Netherlands; 6 Einthoven Laboratory for Experimental Vascular Medicine, Leiden University Medical Center, Albinusdreef 2, Leiden, 2333 ZA, The Netherlands; 7 Department of Biochemistry and Molecular Cell Biology, University Medical Center Hamburg-Eppendorf, Hamburg, Germany; 8 Department of Biomedicine, Aarhus University, Aarhus, Denmark; 9 European Research Institute for the Biology of Ageing (ERIBA), University of Groningen, University Medical Center Groningen, Groningen, The Netherlands; 10 Department of Biochemistry and Biophysics, Weill Cornell Medicine, New York, USA; 11 Department of Laboratory Medicine, University of Groningen, University Medical Center Groningen, Groningen, The Netherlands

**Keywords:** Lysosome, Endosome, Cholesterol, Liver, Cardiovascular disease

## Abstract

Disturbed hepatic cholesterol homeostasis is associated with atherosclerotic cardiovascular disease (ASCVD) and metabolic dysfunction–associated steatotic liver disease (MASLD). The endo-lysosomal system plays a central role in cholesterol uptake and intracellular trafficking, yet the molecular mechanisms governing these processes remain poorly understood. Here, we identify hepatic vacuolar protein sorting 35 (VPS35), a core component of the retromer complex, as a critical regulator of hepatocellular and systemic cholesterol homeostasis. Liver-specific VPS35 ablation in mice elevated plasma and hepatic cholesterol levels due to impaired hepatic uptake of atherogenic lipoproteins, defective cholesterol trafficking through the endo-lysosomal system, and increased *de novo* cholesterol biosynthesis. The decreased hepatic uptake of atherogenic lipoproteins was associated with altered subcellular distribution of the low-density lipoprotein receptor (LDLR) and reduced levels of LDLR-related protein 1 (LRP1). The increased hepatic cholesterol content was associated with altered lysosomal ultrastructure and pH and decreased levels of a wide range of lysosomal proteins, including key proteins for lysosomal cholesterol egress, such as lysosomal acid lipase (LAL), scavenger receptor class B member 2 (SCARB2), Niemann–Pick type C1 (NPC1), and NPC2. Collectively, these findings reveal a previously unrecognized function of the hepatic retromer complex in systemic cholesterol homeostasis. Beyond its established role in endosomal cargo transport, VPS35 is essential for lysosomal cholesterol handling by preserving key proteins involved in lysosomal cholesterol metabolism.

## Highlights

• Hepatic loss of the retromer subunit VPS35 increases plasma LDL cholesterol levels.
• Hepatic VPS35 plays an essential role in the functioning of the lipoprotein receptors LDLR and LRP1.
• VPS35 is essential for lysosomal function in hepatocytes.
• Lysosomal dysfunction in liver-specific VPS35-deficient mice is correlated with reduced lysosomal levels of the lysosomal cholesterol-handling proteins LAL, SCARB2, NPC2, and NPC1, and subsequently with elevated hepatic cholesterol content and *de novo* cholesterol synthesis.
• Hepatic retromer is an essential player in systemic cholesterol homeostasis beyond its role in endosomal transport of integral membrane proteins.

## Graphical abstract

**Figure:**
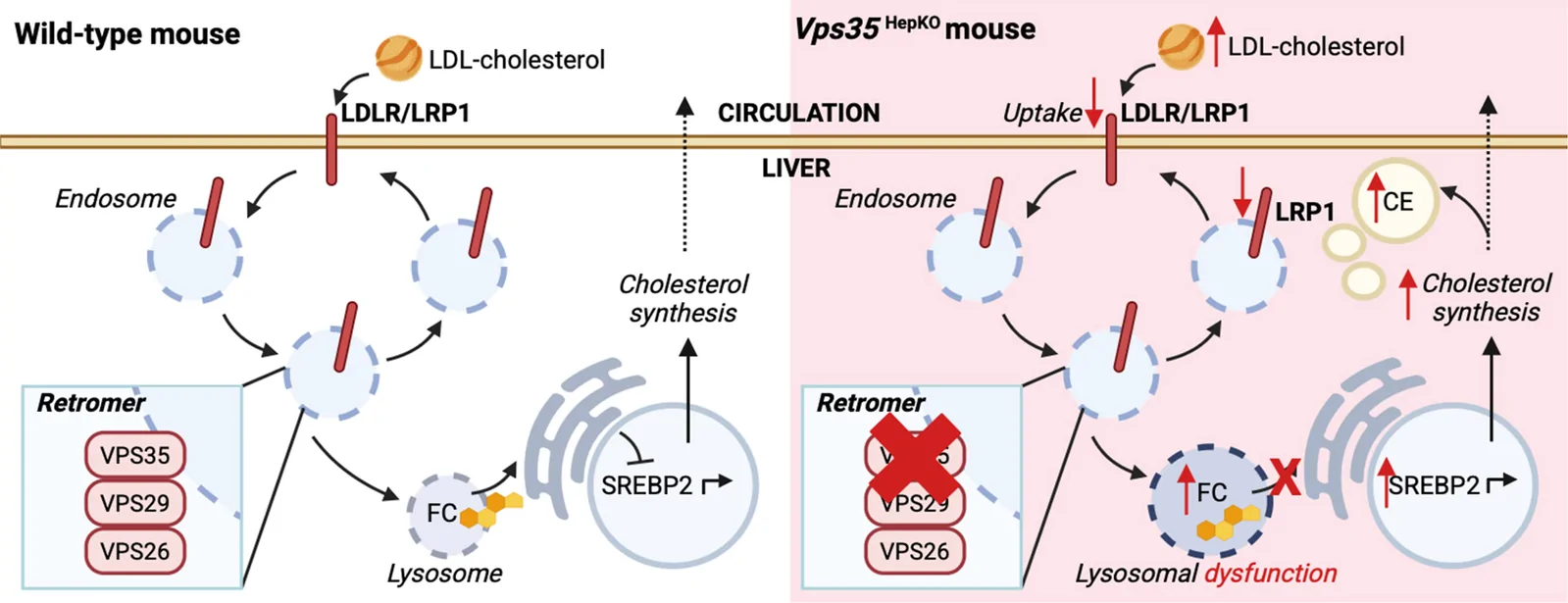

## 1 Introduction

The liver is crucial for maintaining systemic cholesterol homeostasis, and its dysregulation is linked to various diseases, including atherosclerotic cardiovascular disease (ASCVD) and metabolic dysfunction–associated steatotic liver disease (MASLD) [1]. The endo-lysosomal system plays a central role in cholesterol metabolism, as it facilitates receptor-mediated uptake of low-density lipoprotein (LDL) and triglyceride-rich lipoprotein remnants, as well as the intracellular transport of cholesterol towards different compartments, such as plasma membrane (PM), endoplasmic reticulum (ER), and trans-Golgi network (TGN) [2], [3].

Within lysosomes, the lipoprotein-derived cholesteryl esters (CE) are hydrolyzed by lysosomal acid lipase (LAL) to release fatty acids (FA) and free cholesterol (FC) [4]. Lysosomal FC egress is controlled by Niemann-Pick type C proteins (NPCs). The small luminal NPC2 protein binds FC and delivers it to the lysosomal membrane transporter NPC1 [5]. Together, they enable the distribution of FC to the PM, ER, and TGN [3]. The scavenger receptor class B member 2 (SCARB2), also known as lysosomal integral membrane protein type 2 (LIMP2), acts in parallel with NPCs to mediate lysosomal cholesterol export [6]. In the ER, where FC levels are very low under normal conditions [7], cholesterol is sensed by the sterol regulatory-element binding protein (SREBP)/SREBP cleavage-activating protein (SCAP) machinery to regulate cholesterol biosynthesis and LDL-cholesterol uptake in response to the cellular needs [8].

The importance of the endo-lysosomal system in maintaining cholesterol homeostasis is well demonstrated by our recent studies and those of colleagues. For example, we have shown that the endosomal protein sorting complexes, such as the Wiskott-Aldrich syndrome protein and SCAR homolog (WASH) and the COMMD/CCDC22/CCDC93 (CCC) complexes, play a key role in regulating plasma cholesterol levels in mice, dogs, and humans [9], [10], [11]. These two complexes act together to facilitate the endosomal recycling of the LDL receptor (LDLR) and its family member LDLR-related protein 1 (LRP1), which are essential for hepatic lipoprotein uptake (Fig. S1A) [9], [10], [11]. Furthermore, in patients with lysosomal acid lipase deficiency (LAL-D, MIM #613497) or NPC diseases (MIM #257220, MIM #607625), cellular and systemic cholesterol homeostasis is also disturbed. These rare disorders are caused by loss-of-function mutations in *LIPA*, the gene encoding LAL, and *NPCs* genes, respectively [12], [13]. LAL-D is characterized by the accumulation of CE in late endosomes and lysosomes, which mainly affects macrophages, the spleen, and the liver (MIM #613497). Complete loss of LAL in humans results in early-onset Wolman's disease, which is fatal within the first year of life (MIM #620151) [14]. Milder mutations in LAL cause CE storage disease (CESD; MIM #278000), which has a later onset due to 5–10% residual LAL activity, resulting in a range of clinical phenotypes, including hypercholesterolemia, premature atherosclerosis, hepatosplenomegaly, and progressive liver disease [13], [14]. NPC disease (MIM #257220, MIM #607625), characterized by FC accumulation in lysosomes of different organs, is primarily a neurodegenerative disorder [12]. However, it also leads to progressive liver disease associated with hepatosplenomegaly, cholestasis, fibrosis, and an increased risk of hepatocellular carcinoma [12], [15]. To date, no cures are available for either LAL-D or NPC disease, and current treatments only alleviate symptoms [12], [13]. Despite the well-described roles of LAL and NPCs in regulating cellular cholesterol homeostasis, the mechanisms controlling their levels and functions are poorly understood.

Recent studies have implied a role for the endosomal protein sorting machinery complex, retromer, in cholesterol homeostasis. Retromer consists of the vacuolar protein sorting VPS26, VPS29, and VPS35, and mainly localizes to endosomal membranes (Fig. S1A and B). In concert with sorting nexins (SNXs), like SNX27, retromer regulates the endosomal sorting of an array of integral membrane cargo proteins and recycles these to the plasma membrane or the TGN for reuse[16], [17], [18], [19]. Marquer et al. were the first to link retromer dysfunction to aberrant cholesterol homeostasis *in vitro* [20]. They showed that the transport of NPC2 from the TGN to the lysosome is mediated via the cation-independent (CI) mannose-6-phosphate receptor (M6PR). CI-M6PR is a retromer cargo [21], [22], and loss of retromer causes lysosomal cholesterol accumulation in HeLa cells due to impaired CI-M6PR retrograde trafficking [20]. Others have shown that cholesterol accumulation in *in vitro* NPC1-deficient models is associated with reduced retromer function [23]. Furthermore, *in vitro* studies have shown that retromer is essential for the recruitment of the WASH and CCC complexes to endosomes. However, a retromer-independent model for recruiting these two multiprotein complexes to the endosomes has also been proposed (Fig. S1A and B) [24], [25].

Taken together, these studies suggest a relation between retromer and cholesterol homeostasis, but the molecular connection and physiological relevance remain to be fully elucidated. In this study, we reveal a previously unknown role for hepatic retromer in maintaining whole-body cholesterol homeostasis.

## 2 Results

### 2.1 Liver-specific depletion of VPS35 destabilizes retromer without affecting other endosomal sorting complexes

To examine the physiological role of retromer in cholesterol homeostasis, we generated a liver-specific *Vps35* knockout (*Vps35*^HepKO^) mouse model. Using immunoblotting, we confirmed the ablation of *Vps35* in the livers of *Vps35*^HepKO^ mice (Figure 1A). In line with previous studies [26], [27], loss of *Vps35* strongly reduced the protein levels of the other retromer subunits VPS26 and VPS29 by 91% and 83%, respectively (Figure 1A,B). Retromer shares its subunit VPS29 with retriever, which consists of VPS26C, VPS29, and VPS35L (Fig. S1B), and shows structural similarities with retriever [28]. Since retriever is physically and functionally connected with the COMMD1/CCDC22/CCDC93 (CCC) complex (Fig. S1B) [28], [29], we also determined the protein levels of VPS35L and the CCC components CCDC22, CCDC93, and COMMD1. Immunoblotting shows that hepatic loss of *Vps35* did not affect the levels of these proteins (Fig. S2A). To get further insight into the complex formation of the retriever, CCC, and WASH complexes, we determined the relative distribution of the components of these multiprotein complexes within the endo-lysosomal system, using sucrose density gradient fractionation of homogenates from *Vps35*^HepKO^ and control mouse livers. We analyzed the proteome in all fractions by protein mass spectrometry and detected the retromer subunits mainly in fractions 5–7 in control livers (Fig. S2B) and confirmed the marked reduction of the subunits upon *Vps35* ablation (Fig. S2B). The components of the retriever, CCC, and WASH complexes were mainly present in fractions 9–14, and loss of *V35* did not affect their relative distribution in the gradient (Fig. S2B). These data indicate that hepatic *Vps35* ablation specifically impairs retromer function without affecting the formation of retromer-associated protein complexes, such as retriever, CCC, and WASH.

**Figure 1:**
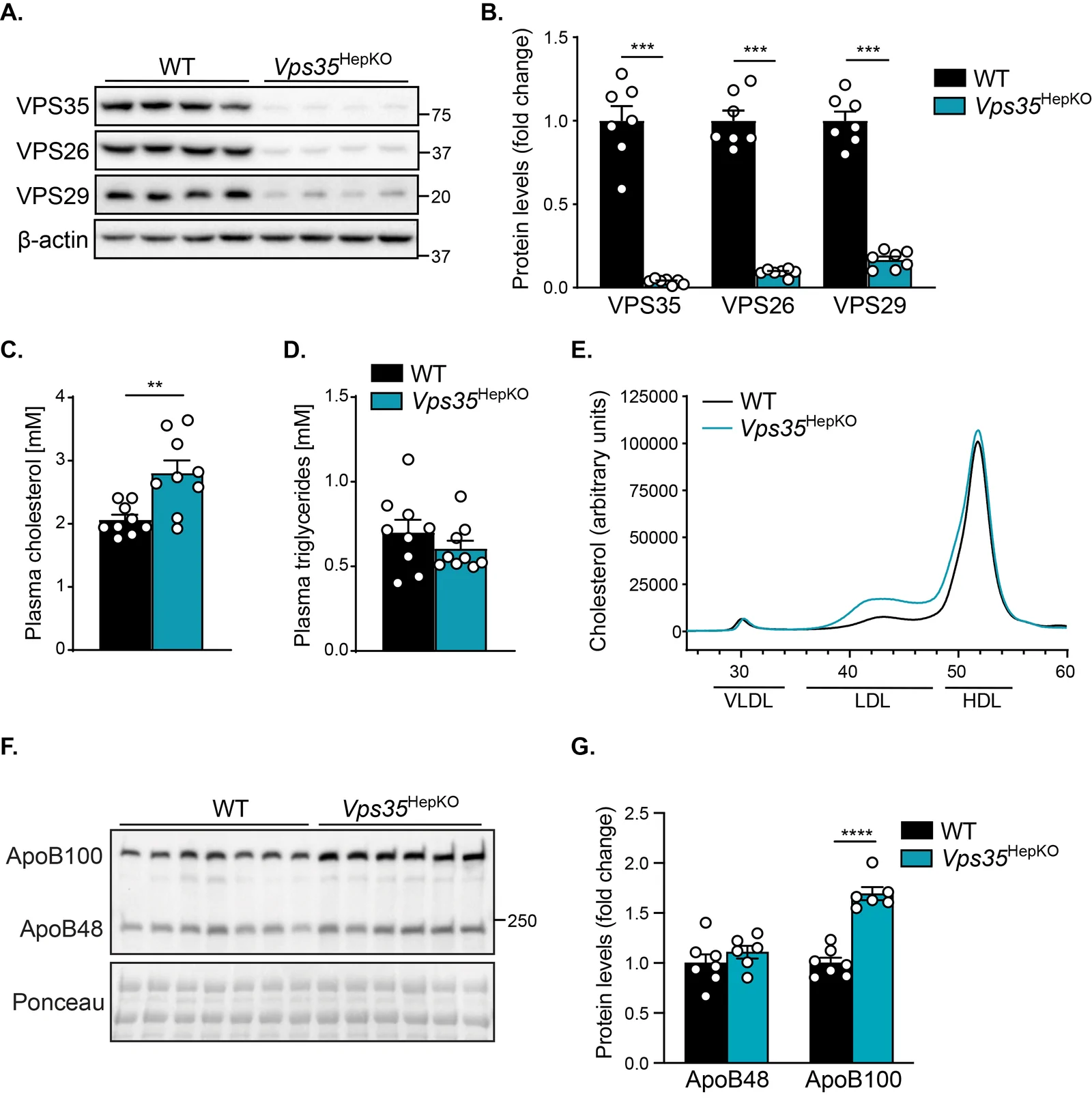
**Liver-specific VPS35-deficient male mice are hypercholesterolemic.** (**A**) Hepatic protein levels of the retromer subunits VPS35, VPS26, and VPS29 were determined by immunoblotting (n = 4). (**B**) Quantification of hepatic protein levels of the retromer subunits, as determined by immunoblotting (n = 7). (**C**) Plasma cholesterol and (**D**) TG levels in WT and *Vps35*^HepKO^ male mice (n = 9) fed a standard laboratory diet. (**E**) Cholesterol levels in plasma fractionated by FPLC (n = 7). (**F**) ApoB levels in plasma, determined by immunoblotting (n = 6–7). (**G**) Quantification of ApoB levels shown in **F** (n = 6–7). Data are presented as mean ± SEM; ⁎⁎p < 0.01 and ⁎⁎⁎p < 0.001.

### 2.2 Hepatic VPS35 deficiency increases plasma cholesterol levels

While *Vps35*^HepKO^ male mice were born at the expected Mendelian ratio (data not shown), the body and liver weights of these animals were slightly higher compared to their wild-type (WT) littermates (Fig. S3A and B). To determine the effect of hepatic *Vps35* depletion on plasma lipid levels, we measured cholesterol and triglyceride (TG) concentrations in plasma samples. We found that *Vps35*^HepKO^ mice showed a 36% increase in plasma cholesterol levels compared to control mice (Figure 1C), while plasma TG levels were not different between control and *Vps35*^HepKO^ mice (Figure 1D). Fast protein liquid chromatography (FPLC) separation showed that the elevated plasma cholesterol levels in *Vps35*^HepKO^ mice were mainly caused by an increase in LDL cholesterol (Figure 1E), which was supported by a significant increase in plasma levels of ApoB100 (Figure 1F,G).

### 2.3 Hepatic ablation of VPS35 reduces hepatic LDL cholesterol uptake due to impaired LDLR and LRP1 functioning

Retromer is physically associated with the WASH complex, a complex that acts together with the CCC complex to recycle LDLR and LRP1 from endosomes back to the PM (Fig. S1A and B) [9], [10], [11]. Since depletion of either WASH or CCC reduces surface LDLR and LRP1 levels in primary hepatocytes, accompanied by elevated plasma LDL cholesterol levels in mice [9], [10], [11], we studied the hepatic levels of both receptors in *Vps35*^HepKO^ and control mice. We found that loss of VPS35 resulted in a 50% increase in hepatic LDLR protein levels and a 40% decrease in LRP1 levels (Figure 2A,B). These changes in protein levels could not be explained by their respective mRNA levels (Fig. S3C). To further explore the effect of hepatic VPS35 deficiency on the localization of LDLR and LRP1 at the PM, we performed an *in vivo* PM biotinylation experiment. VPS35 deficiency reduced the protein levels of LRP1 at the PM but not LDLR at the PM (Figure 2C). The decrease in surface LRP1 levels, without affecting surface LDLR, was confirmed by isolating PM fractions from WT and *Vps35*^HepKO^ livers (Figure 2D).

**Figure 2:**
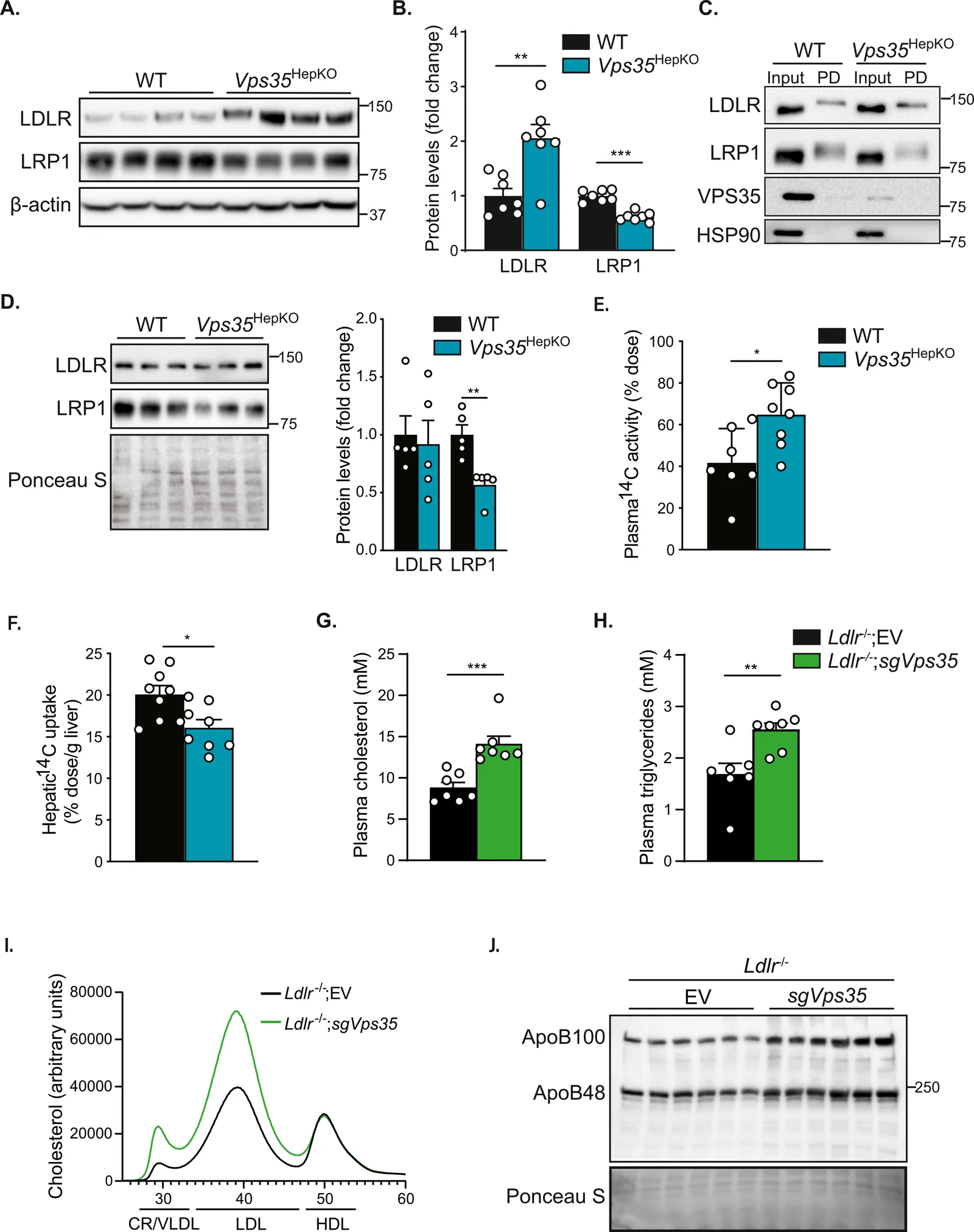
**Hepatic VPS35 is required for LDLR and LRP1 function to clear apoB-containing lipoprotein particles.** (**A**) Immunoblot of LDLR and LRP1 in livers of WT and *Vps35*^HepKO^ male mice (n = 4) fed a standard laboratory diet. (**B**) Quantification of LDLR and LRP1 protein levels (n = 7), as determined by immunoblotting. (**C**) *In vivo* cell surface biotinylation was performed in WT and *Vps35*^HepKO^ male mice. LDLR and LRP1 were detected in total liver lysates (input, 0.5%) and the isolated cell surface protein fraction by immunoblotting. PD = pull-down. (**D**) LDLR and LRP1 at the plasma membrane after enrichment of the plasma membrane. Quantification of LDLR and LRP1 levels at the plasma membrane, as determined by immunoblotting (n = 5). (**E**) Plasma ^14^C activity and (**F**) hepatic ^14^C activity 2 h after i.v. administering [^14^C]cholesteryl oleate (CO)-labelled human LDL in WT and *Vps35*^HepKO^ male mice (n = 6–9). (**G**) Plasma cholesterol and (**H**) TG levels, and (**I**) cholesterol in plasma fractionated by FPLC of LDLR-deficient and sg*Vps35*; LDLR-deficient male mice (n = 7). (**J**) Immunoblot of ApoB in plasma of LDLR-deficient and sg*Vps35*; LDLR-deficient male mice (n = 6). Data are presented as mean ± SEM; ⁎p < 0.05, ⁎⁎p < 0.01 and ⁎⁎⁎p < 0.001. (**J**) Quantification of APOB48 and APOB100 protein levels (n = 6) determined by immunoblotting.

Next, using our sucrose density gradient fractionation proteomics data, we assessed the relative distribution of LDLR and LRP1 within the various subcellular compartments (Fig. S2B). The total levels of LDLR were increased, and the relative distribution of LDLR was changed upon hepatic *Vps35* depletion (Fig. S2B). In control livers, the majority of LDLR is present in fractions 13–15, while in *Vps35*^HepKO^ livers, LDLR is mainly present in fractions 10–13 and 15, which partially co-sediment with the endosomal protein sorting complexes WASH and CCC, and the lysosomal marker LAMP1 (Fig. S2B). The relative distribution of LRP1 was also shifted to fractions 10–13, and the total levels were decreased in VPS35-deficient livers (Fig. S2B).

Because retromer retrieves cargoes away from lysosomes, we studied the protein levels of these receptors in lysosomes. We immunoprecipitated intact lysosomes from liver homogenates of transgenic mice expressing the lysosomal protein TMEM192 fused with a 3xHA peptide (LysoTag mice) [30] (Fig. S4A and B). We crossed these LysoTag mice with liver-specific Cas9-expressing mice [29]. Using a somatic CRISPR/Cas9-mediated gene-editing approach (Fig. S4A and B), we deleted VPS35 in LysoTag mice. Immunoprecipitated lysosomes (LysoIPs) from LysoTag mice, but not from LysoTag negative control mice, were markedly enriched for the lysosomal marker LAMP2A (Fig. S4C). Consistent with initial findings (Figure 2A,B), total LDLR levels were increased, and LRP1 levels were reduced in VPS35-deficient livers (Fig. S4C and D). When correcting for the lysosomal protein LAMP2A, LDLR was enriched whereas LRP1 levels were unchanged upon VPS35 ablation (Fig. S4C and E). These data indicate that a fraction of LDLR accumulates in lysosomal compartments upon hepatic VPS35 ablation.

Next, we assessed how these alterations in subcellular LDLR distribution affect hepatic LDL uptake *in vivo*. We injected control and *Vps35*^HepKO^ male mice with [^14^C]cholesteryl oleate (CO)-labelled human LDL, and mice were sacrificed 2 h after labelled LDL administration to measure [^14^C]CO-derived activity in plasma and liver. We confirmed the elevated plasma cholesterol levels, without affecting TG levels, in *Vps35*^HepKO^ male mice at the moment of administration of radiolabelled LDL (Fig. S5A and B). The ^14^C activity in the plasma of *Vps35*^HepKO^ mice was significantly higher, while the ^14^C activity in the liver was significantly reduced compared to control mice (Figure 2E,F), indicating that hepatic LDL uptake was impaired in liver-specific VPS35-deficient mice.

To directly assess the role of VPS35 in LRP1 function [31], we ablated hepatic *Vps35* in *Ldlr* knockout (*Ldlr*^−/−^) mice by somatic CRISPR/Cas9-mediated gene editing (Fig. S5C). Four weeks after injection of adeno-associated virus (AAV) encoding sgRNAs targeting *Vps35* into *Ldlr*^−/−^ mice (Fig. S5D and E), we found that hepatic loss of VPS35 significantly increased plasma cholesterol and TG levels compared to control *Ldlr*^−/−^ mice (Figure 2G,H). These increases were caused by elevated chylomicron remnant/VLDL and LDL levels, as determined by FPLC separation (Figure 2I, Fig. S5F), and supported by a significant increase in plasma levels of ApoB48 and ApoB100 (Figure 2J, Fig. S5G). This phenotype shows similarities with the lipid phenotype seen in mice deficient in LDLR and hepatic LRP1 [31].

Taken together, these data indicate that retromer is required for LDLR and LRP1 function in mouse livers, enabling uptake of atherogenic lipoproteins.

### 2.4 Hepatic VPS35 deficiency impairs intracellular cholesterol trafficking and increases *de novo* cholesterol synthesis

To gain a deeper understanding of how hepatic VPS35 deficiency affects cholesterol homeostasis, we also studied cholesterol fluxes in *Vps35*^HepKO^ and control mice [32]. We administered D_7_-labeled cholesterol orally and measured tracer enrichment in the blood for seven consecutive days (Figure 3A). We calculated cholesterol-D_7_ turnover kinetics by curve fitting and found that the rate of cholesterol-D_7_ secretion from the liver into circulation was markedly delayed in *Vps35*^HepKO^ mice compared with controls (Figure 3A,B). Yet, fractional intestinal cholesterol absorption was similar between groups (Figure 3C). These data indicate that after receptor-mediated endocytosis of D_7_-cholesterol-containing chylomicron remnants, hepatic secretion of chylomicron-derived cholesterol into the circulation is delayed in *Vps35*^HepKO^ mice compared to controls.

**Figure 3:**
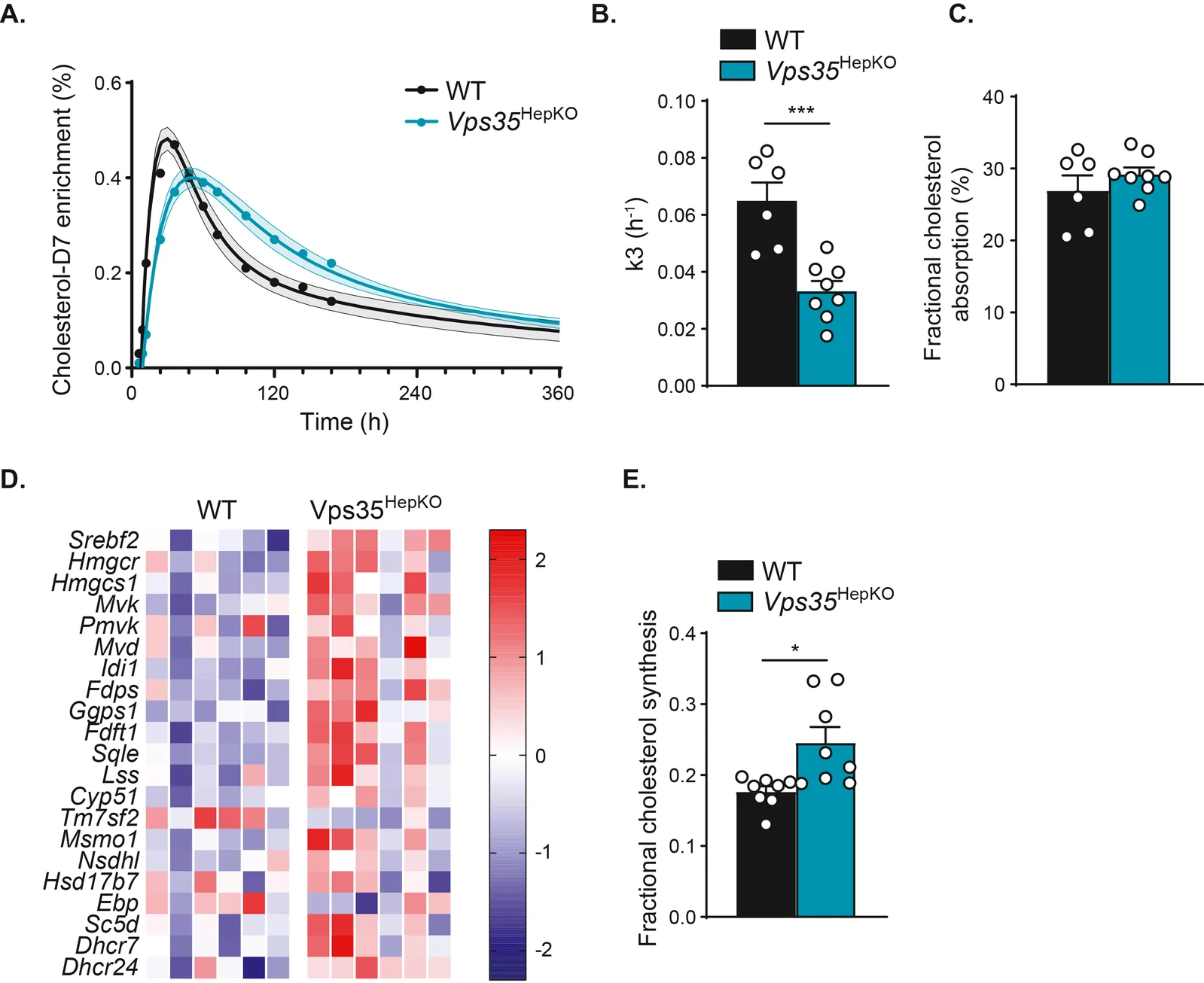
**Hepatic secretion of endocytosed cholesterol is impaired, while cholesterol biosynthesis is increased in *Vps35*^HepKO^ male mice.** (**A**) Fractional enrichment of orally administered cholesterol-D_7_ in WT and *Vps35*^HepKO^ mice (n = 6–8). (**B**) Kinetic parameter *k3* describing the rate of cholesterol-D_7_ secretion by the liver into the circulation in WT and *Vps35*^HepKO^ male mice (n = 6–8). (**C**) Fractional intestinal cholesterol absorption in WT and *Vps35*^HepKO^ mice (n = 6–8). (**D**) Heatmap presenting the z-score normalized expression of genes in the cholesterol biosynthesis pathway in livers of WT and *Vps35*^HepKO^ male mice (n = 6) fed a standard laboratory diet. (**E**) Fractional cholesterol synthesis in WT and *Vps35*^HepKO^ mice (n = 8) fed a standard laboratory diet. Data are presented as mean ± SEM; ⁎p < 0.05 and ⁎⁎⁎p < 0.001.

This observation suggests that hepatic *Vps35* ablation compromises transport of endocytosed cholesterol through the endo-lysosomal system to the ER. Impaired endo-lysosomal cholesterol transport to the ER is accompanied by increased SREBP-mediated *de novo* cholesterol biosynthesis [3]. Consistent with this, we found, using transcriptomic analysis, that the SREBP2-driven cholesterol biosynthesis is upregulated in *Vps35*^HepKO^ mice, with pronounced upregulation of 3-hydroxy-3-methylglutaryl-CoA synthase 1 *(Hmgcs1),* mevalonate kinase *(Mvk)*, and squalene epoxidase *(Sqle)* (Figure 3D). To assess the physiological consequences of these observations, we studied *de novo* cholesterol synthesis over time. To this end, we gave mice drinking water containing the labeled cholesterol precursor ^13^C-acetate for 5 days and measured the incorporation of this tracer into cholesterol from bloodspots. We then applied mass isotopomer distribution analysis (MIDA) to calculate fractional cholesterol synthesis [33], [34]. We found that loss of hepatic VPS35 significantly increased whole-body cholesterol synthesis rates (Figure 3E), which predominantly reflect hepatic cholesterol synthesis in mice [35]. These data show that retromer plays an essential role in cholesterol homeostasis, likely by regulating intracellular cholesterol trafficking and, consequently, affecting cholesterol synthesis.

### 2.5 *Vps35*^HepKO^ male mice accumulate both free and esterified cholesterol in their livers

To study the effect of compromised intracellular trafficking in *Vps35*^HepKO^ mice on total hepatic lipid levels, we measured lipid content in *Vps35*^HepKO^ and control male mice. Hepatic TC content in *Vps35*^HepKO^ male mice was significantly increased compared to control mice (Figure 4A), while TG concentrations tended to decrease (Figure 4B). The increase in TC was caused by higher FC as well as CE (Figure 4A). Using lipidomics, we confirmed the increase in CE (+46%) and found a significant 26% decrease in hepatic TG levels (Fig. S6A).

**Figure 4:**
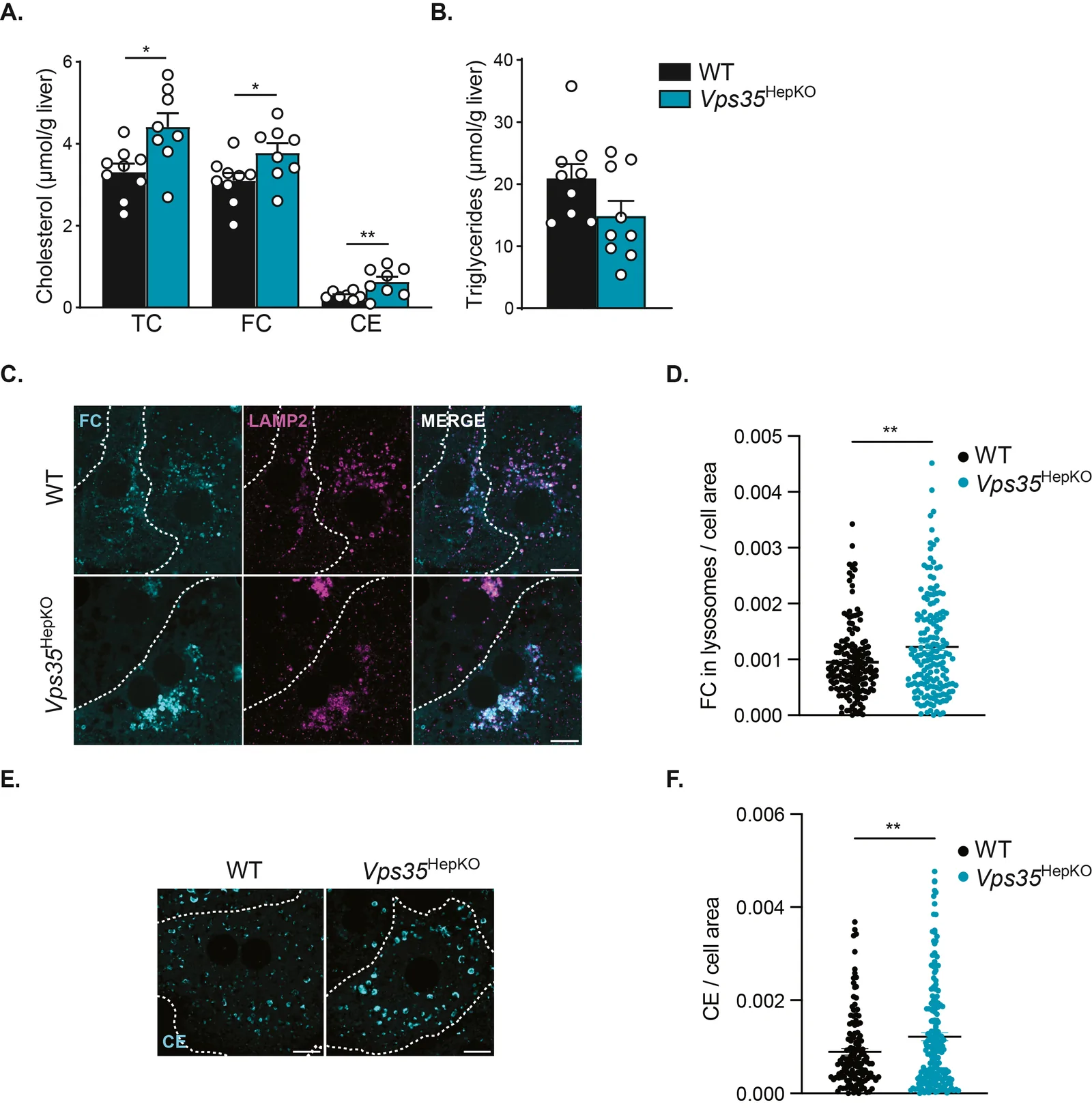
**Hepatic *Vps35* depletion increases hepatic cholesterol content in male mice.** (**A**) Hepatic cholesterol (n = 7–9) and (**B**) TG (n = 9) levels in WT and *Vps35*^HepKO^ male mice fed a standard laboratory diet. (**C**) Airyscan fluorescence microscopy images of wild-type (WT) and VPS35-deficient mouse primary hepatocytes stained with filipin to detect free cholesterol (FC) and LAMP2 antibody to detect lysosomes. Cell boundaries are indicated by dashed lines, and these are sections of larger images as shown in Fig. S7A. Scale 10 μm. (**D**) Quantification of FC (filipin intensity) in lysosomes in each imaged cell normalized to cell area. n = 162 cells for WT and 168 cells for VPS35-deficient cells. (**E**) Airyscan fluorescence microscopy images of WT and VPS35-deficient mouse primary hepatocytes treated with ethanol to extract free cholesterol, incubated with cholesterol esterase, and stained with filipin to detect cholesteryl esters (CE). Cell boundaries indicated by dashed lines. Scale 10 μm. (**F**) Quantification of CE (filipin intensity) in each imaged cell normalized to cell area. (n = 135 cells for WT and 182 cells for VPS35-deficient cells). All data are presented as mean ± SEM; ⁎p < 0.05, and ⁎⁎p < 0.01.

The increase in hepatic cholesterol content was accompanied by elevated plasma levels of the liver enzymes ALT and AST (Fig. S6B), suggesting that hepatic loss of VPS35 induces liver damage. However, histological examination using hematoxylin and eosin (H&E) and TUNEL staining did not reveal overt liver damage or hepatocellular apoptosis, respectively (Fig. S6C and D). In addition, we found increased expression of the monocyte/macrophage marker *Cd68* and monocyte chemoattractant protein 1 (*Mcp1*), but no significant changes in other inflammatory markers, such as *Il1b*, *Il6*, *Tnf*, and *F4/80* (Fig. S6E). These findings show that hepatic retromer is essential to maintain hepatic and plasma cholesterol homeostasis, and its loss leads to mild liver damage and inflammation.

### 2.6 Hepatic VPS35 deficiency increases cholesterol content in lysosomes and lipid droplets

To assess in which subcellular compartment cholesterol accumulates upon loss of VPS35, we stained intracellular free cholesterol (FC) with filipin. We observed that ablation of VPS35 in primary hepatocytes results in a significant increase in FC accumulation within LAMP2-positive compartments (Figure 4C,D, Fig. S7A), which redistributed to a perinuclear localization, while in WT cells filipin/LAMP2-positive compartments were dispersed throughout the cytoplasm (Figure 4C). To assess CE subcellular accumulation, we first extracted FC from WT and VPS35-deficient primary hepatocytes, then converted intracellular CE to FC and stained the newly formed FC with filipin. First, we validated the efficiency by which ethanol extraction removes the filipin-detectable FC from cells. As shown in Fig. S7B, most of the filipin-detectable FC is removed from cells after ethanol extraction. Using this approach, we found that VPS35 deficiency also increased the subcellular CE levels (Figure 4E,F), however, in contrast to FC, CE accumulation was primarily observed in perilipin-3 (PLIN3)-positive lipid droplets (Fig. S7C). These data suggest that hepatic VPS35 loss results in the accumulation of FC in late endosomes/lysosomes and CE in lipid droplets.

### 2.7 Hepatic VPS35 loss alters the endo-lysosomal structure and decreases the levels of a subset of lysosomal proteins involved in intracellular cholesterol transport

Lysosomes play an essential role in cholesterol homeostasis by directing cholesterol to other subcellular compartments, including the ER. Previous *in vitro* work has shown that VPS35 deficiency causes changes in late endosomal/multivesicular body (MVB) and lysosomal structures, as well as aberrant lysosomal functions [26], [27], [36], [37], [38]. For these reasons, we analyzed the ultrastructure of MVBs and the degradative compartments, i.e., lysosomes and autolysosomes/amphisomes, in VPS35-deficient hepatocytes using scanning electron microscopy. We found that the number of degradative compartments and MVBs was significantly increased in these cells compared to the WT controls (Figure 5A,B, C).

**Figure 5:**
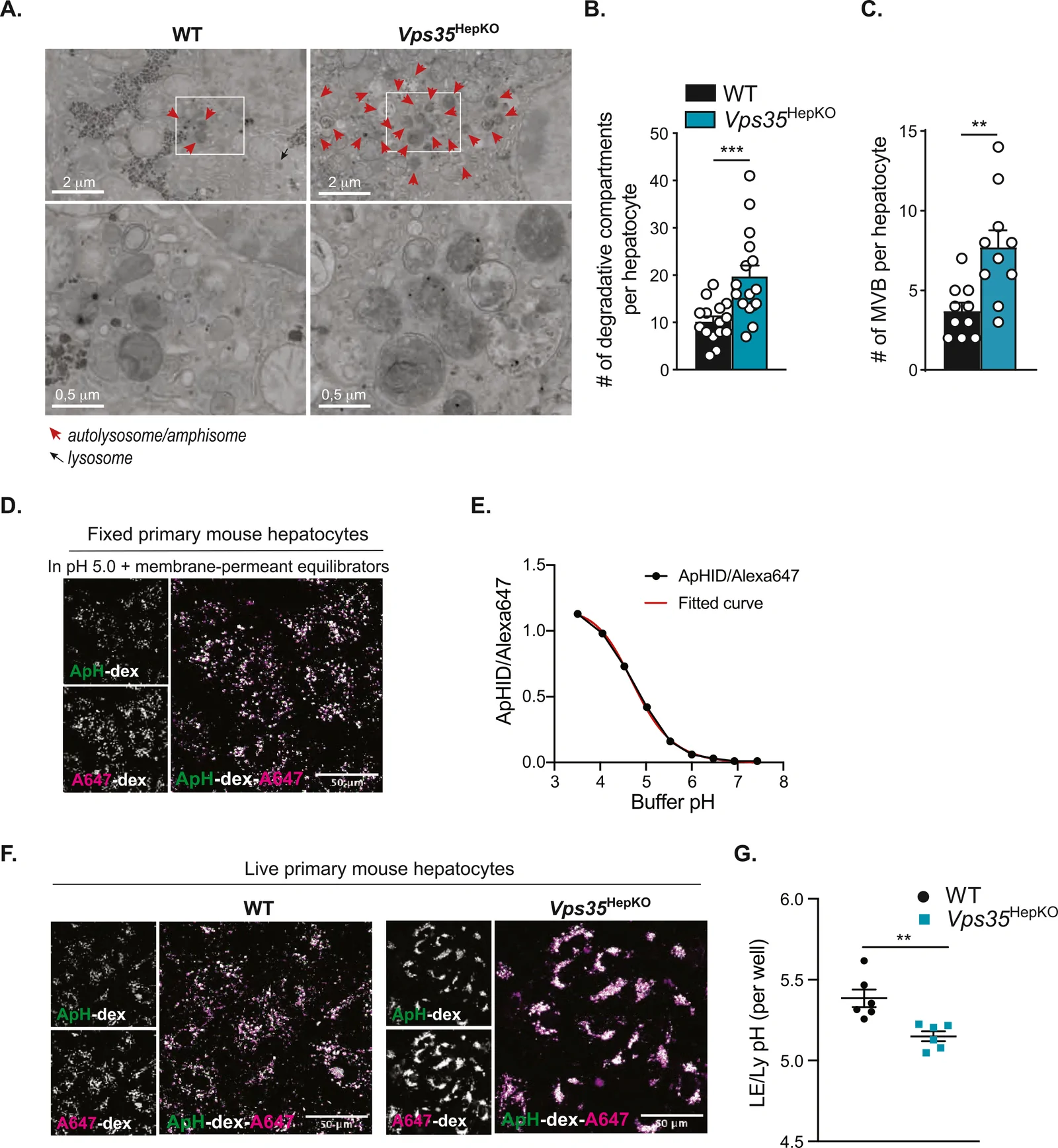
**Hepatic loss of VPS35 affects the ultrastructure and the pH of the endo-lysomal system.** (**A**) Scanning transmission electron microscopic images of livers of WT and *Vps35*^HepKO^ male mice fed a standard laboratory diet, where amphisomes, autolysosomes, and lysosomes are indicated. Full datasets are available upon request. (**B**) Quantification of degradative compartments and (**C**) multivesicular body (MVB) in WT and *Vps35*^HepKO^ hepatocytes. (**D**) Calibration of the ApHID probe in fixed primary mouse hepatocytes. Cells were pulsed with ApHID-dextran (ApH-dex) and Alexa Fluor 647-dextran (A647-dex, pH independent), chased, fixed and equilibrated in a buffer of pH 5.0 (n = 4 wells per genotype). Representative images of WT cells. (**E**) Ratio-to-pH calibration curve. The ApHID/A647 ratio measured at pH 5.0 in (**D**) was scaled to the dynamic range reported for ApHID [39] at 37 °C, and fitted to a four-parameter sigmoidal function. (**F**) Representative live-cell images of primary hepatocytes isolated from WT and *Vps35*^HepKO^ mice, pulsed with ApH—A647-dextrans. (**G**) Late endosomal/lysosomal pH in live cells, obtained by translating ApHID/A647 ratios to pH by interpolation to the calibration curve in (E) (n = 6 per well, 4–6 fields per well; each symbol represents one well). All data are presented as mean ± SEM; ⁎⁎p < 0.01 and ⁎⁎⁎p < 0.001, scale bars 50 μm.

To assess whether the ultrastructural changes in degradative compartments and MVBs were accompanied by altered lysosomal function, we measured the late endosomal/lysosomal pH in primary VPS35-deficient hepatocytes using the pH sensor ApHID [39]. We found that the late endosomal/lysosomal pH was markedly more acidic in VPS35-deficient hepatocytes (pH ∼5.1) compared to control hepatocytes (pH ∼5.4) (Figure 5D–G). In addition, we observed that late endosomal/lysosomal compartments were redistributed to a perinuclear localization upon the loss of VPS35, consistent with the perinuclear redistribution of LAMP2-positive compartments observed by filipin staining (Figure 4E).

To further understand the effect of VPS35 ablation on the endo-lysosomal system, we examined the levels of late endosomal/lysosomal proteins by whole-liver proteomics. We found that multiple lysosomal proteins were unaffected by the loss of VPS35 (Figure 6A), while some were significantly increased, including the V-ATPase subunits VATC1 and VATB2, cathepsin B, and NPC2, or decreased, including RAB7A, TPP1, RISC, cathepsin A/PPGB, and LAL (Figure 6A). Interestingly, a marked reduction was also observed in SCARB2 levels. The lysosomal protein NPC1 was not identified in the proteomic approach, however, due to its essential role in lysosomal cholesterol export, we determined its levels by immunoblotting, together with those of SCARB2. NPC1 levels, like those of SCARB2, were also strongly decreased in *Vps35*^HepKO^ livers compared to controls (Figure 6B,C). In line with its decreased protein levels, we found that LAL activity was significantly reduced in *Vps35* KO cells compared to controls (Figure 6D). The altered levels of NPC1, NPC2, SCARB2, and LAL were not due to changes in mRNA levels (Figure 6E), suggesting that they were the consequence of a post-transcriptional event.

**Figure 6:**
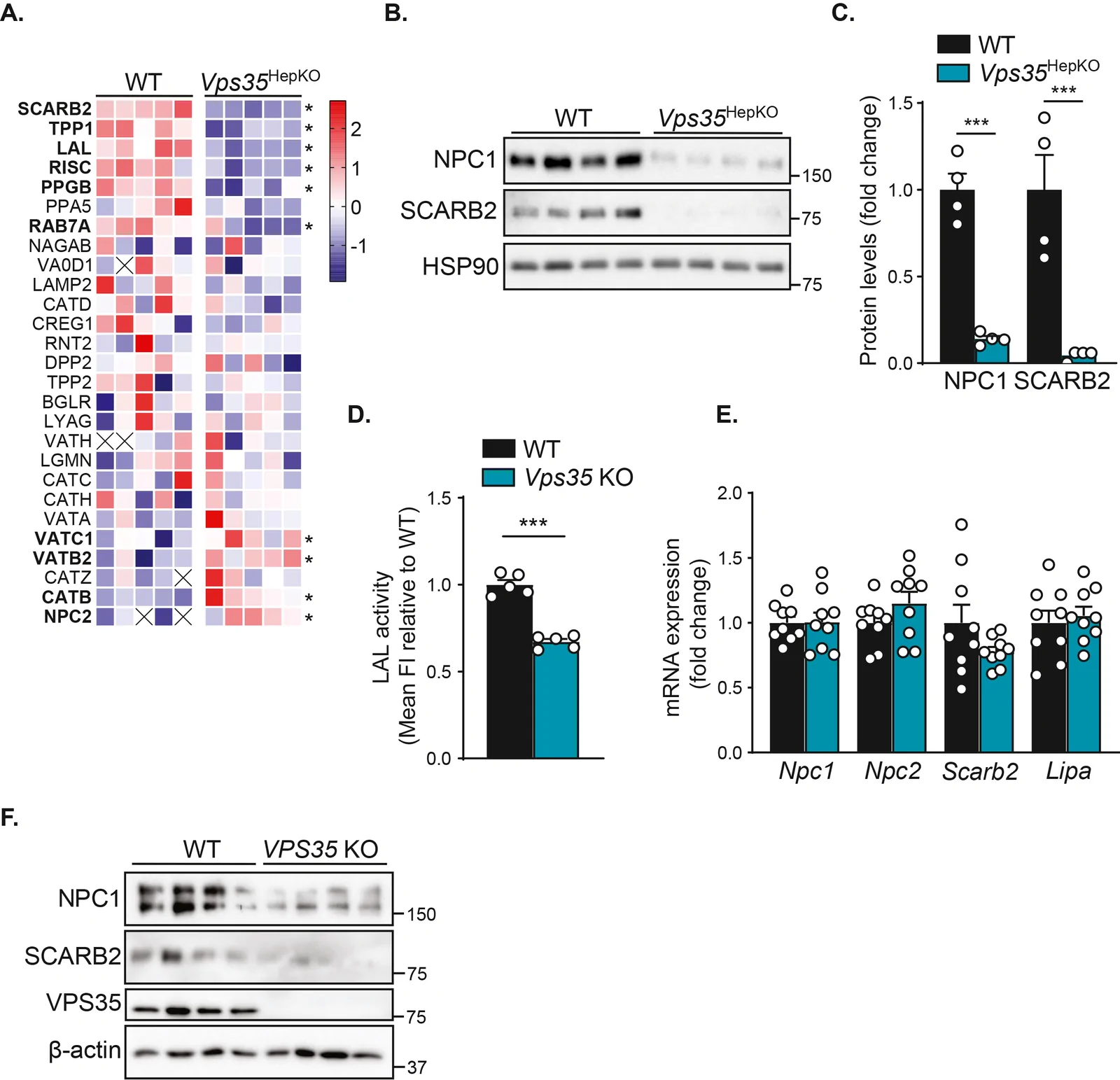
**Hepatic VPS35 deficiency reduces the expression of a specific subset of lysosomal proteins, including lysosomal cholesterol-handling proteins.** (**A**) Heatmap showing z-scores of protein levels of LE/lysosomal proteins, presented as protein names, calculated from raw counts measured with untargeted proteomics (n = 5); significantly changed proteins are indicated in bold and with ⁎. (**B**) Immunoblot of NPC1 and SCARB2 in WT and *Vps35*^HepKO^ male livers (n = 4) fed a standard laboratory diet. (**C**) Quantification of NPC1 and SCARB2 protein levels (n = 4), as determined by immunoblotting (**B**). (**D**) LAL activity, expressed as mean fluorescence intensity, in WT and VPS35–KO Hepa1-6 cells, determined by FACS analysis (n = 5 from two independent experiments). (**E**) Relative mRNA expression of *Npc1*, *Npc2*, *Scarb2,* and *Lipa* in WT and *Vps35*^HepKO^ male livers (n = 9). (**F**) NPC1 and SCARB2 protein levels in WT and VPS35-deficient Human iPSC-derived liver organoids (WT and VPS35 knockout iPSC clones were differentiated into liver organoids, data present 4 independent differentiation experiments). Unless indicated otherwise, data are presented as mean ± SEM; ⁎⁎⁎p < 0.001.

A reduction in NPC1, SCARB2, and LAL was also observed in adult female *Vps35*^HepKO^ mice (Fig. S8A and B), which was accompanied by an increase in hepatic TC and FC content (Fig. S8C). Interestingly, in contrast to male mice, hepatic CE content was similar between WT and *Vps35*^HepKO^ female mice, despite the reduction of LAL in *Vps35*^HepKO^ livers (Fig. S8B). Furthermore, plasma cholesterol and ApoB100 levels were not increased in *Vps35*^HepKO^ female mice, when compared to control mice (Fig. S8D–F), indicating that the effect of VPS35 deficiency on hepatic CE accumulation and plasma LDL cholesterol is sex-specific in our model.

To assess whether the reduced levels of NPC1, SCARB2, and LAL result from compensatory adaptations to chronic retromer deficiency starting at birth, we deleted VPS35 in young adult male mice (8–9 weeks old) using a somatic CRISPR/Cas9-mediated gene editing approach as shown in Fig. S4A and B. Two weeks after *Vps35* ablation (Fig. S8G), we killed the mice and examined NPC1 and SCARB2 levels. Acute hepatic ablation of VPS35 in adult male mice strongly reduced NPC1 and SCARB2 levels compared to the control mice (Fig. S8H), suggesting that the effect of retromer deletion on these specific lysosomal proteins is likely not an adaptive mechanism.

Collectively, these data show that hepatic loss of retromer alters the ultrastructure and pH of late endosomes/lysosomes and is accompanied by reduced levels of key proteins required for lysosomal cholesterol handling, such as NPC1, SCARB2, and LAL.

### 2.8 Lysosomal proteomics reveals widespread changes in the lysosomal proteome of VPS35-deficient livers

To further compare the lysosomes of VPS35-deficient livers with controls, we studied the lysosomal proteome of immunoprecipitated lysosomes (Fig. S4A–C) by mass spectrometry. Immunoprecipitated lysosomes (LysoIPs) from LysoTag mice were heavily enriched for lysosomal membrane and luminal proteins compared to LysoTag negative control mice (Figure 7A). Principal component analysis of the 2986 identified proteins separated the two genotypes along PC1 (40.5% of variance) (Figure 7B). Differential abundance analysis identified 648 proteins that differed significantly between genotypes (FDR <0.05, log2FC > 0.585 or < -0.585), of which 324 were decreased and 360 increased (Figure 7C). Restricting the analysis to the 509 proteins annotated to endo-lysosomal compartments revealed a clear directional effect, with 121 proteins decreased and 60 proteins increased in VPS35-deficient lysosomes (Figure 7D). In line with the reduction of the total NPC1, SCARB2 and LAL levels in VPS35-deficient livers compared to controls, the lysosomal levels of these proteins were also markedly decreased. In addition, a wide range of lysosomal luminal enzymes (e.g., cathepsins, glycosidases, and sulfatases), and lysosomal-associated proteins involved in lysosomal localization (BORC/HOPS complex), autophagy, and mTOR signaling were affected (Figure 7E). The lysosomal NPC2 levels were strongly reduced, whereas whole-liver NPC2 levels were increased (Figure 6A). The opposite was observed for RAB7A. Lysosomal RAB7A levels were enriched in VPS35-deficient lysosomes compared to controls (Figure 7E), while the total levels were reduced (Figure 6A). Taken together, these data show that loss of VPS35 markedly affects the lysosomal proteome and that lysosomal levels of the cholesterol-handling proteins LAL, NPC1, NPC2, and SCARB2 are significantly reduced.

**Figure 7:**
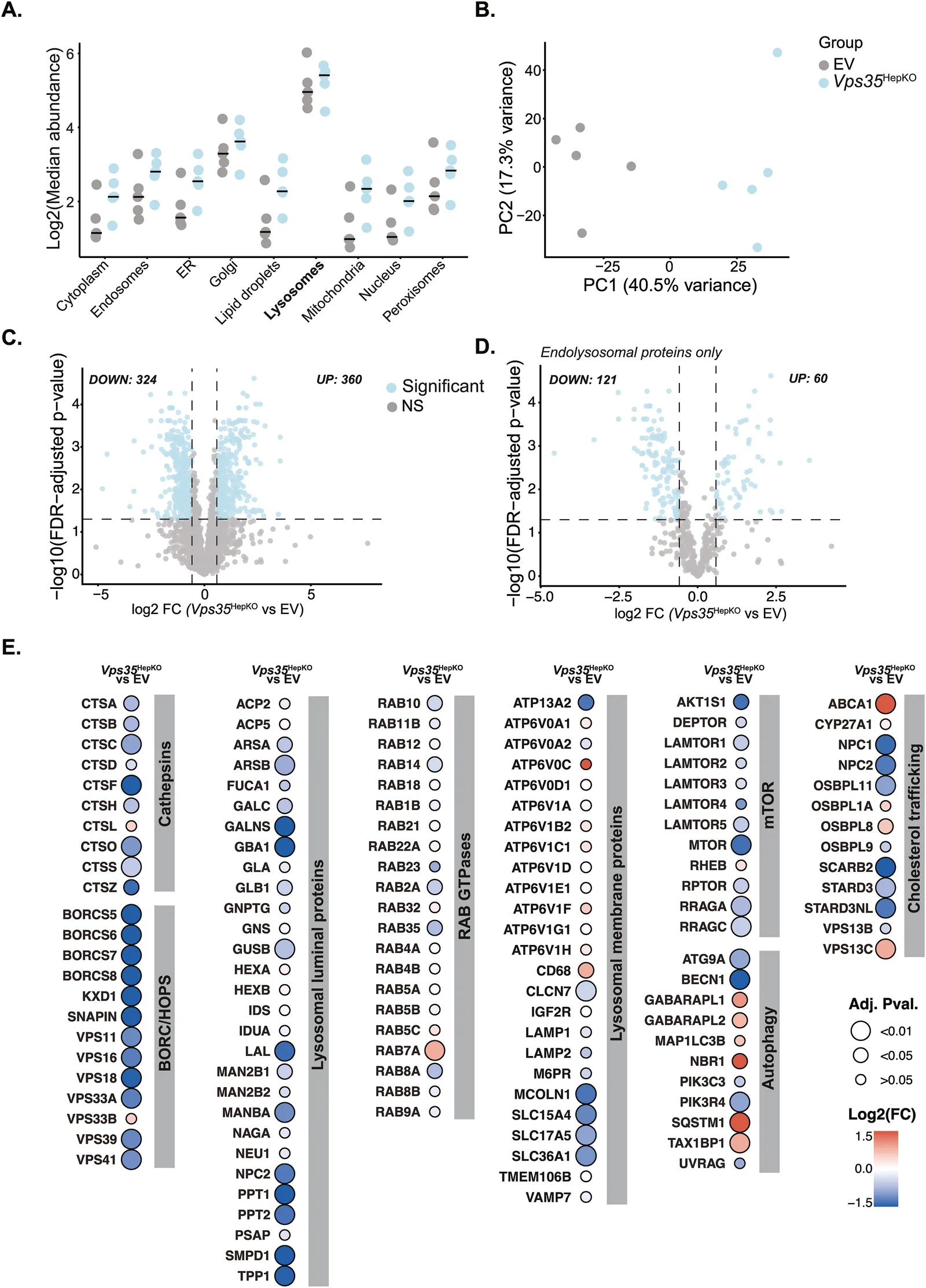
**The proteome of VPS35-deficient lysosomes is markedly affected compared to controls.** Lysosomes were immunoprecipitated (LysoIP) from livers of control (EV) and liver-specific VPS35-deficient mice and analyzed by mass spectrometry (n = 5). (**A**) Organelle representation of the LysoIP proteome. Proteins were grouped by GO-annotated subcellular compartment and their median abundance in EV and *Vps35*^HepKO^ LysoIP samples was expressed relative to LysoIP from LysoTag-negative control livers. Lysosomal proteins are the most strongly enriched fraction in both genotypes, confirming the LysoIP specificity. (**B**) Principal component analysis of protein intensities across all LysoIP samples; each symbol is one mouse and the variance explained by each component is indicated on the axes. (**C**) Volcano plot of all quantified proteins. Data were normalized to the global median and are presented as the log_2_ ratio of *Vps35*^HepKO^ versus EV; dashed lines mark the applied thresholds (log_2_ fold change > or < 0.585 and Benjamini–Hochberg FDR-adjusted P < 0.05). 360 proteins were significantly increased and 324 significantly decreased in lysosomes from VPS35-deficient livers. (**D**) As in (C), with the same thresholds, restricted to proteins annotated as endo-lysosomal, these shift predominantly downwards upon deletion of VPS35 (121 decreased versus 60 increased). (**E**) Individual proteins, grouped by functional category, that were decreased or increased in lysosomes from VPS35-deficient livers relative to EV controls. Dot color encodes the log_2_ fold change and dot size the FDR-adjusted P value. For display, the color scale is capped at ±1.5; proteins exceeding this range are shown at the scale limit.

### 2.9 Loss of VPS35 in hiPSC-derived liver organoids decreases NPC1 and SCARB2

To translate our findings to humans, we ablated *VPS35* in human-induced pluripotent stem cells (hiPSCs) using CRISPR/Cas9-mediated gene editing. We successfully targeted VPS35, resulting in the complete absence of VPS35 protein expression in hiPSCs (Fig. S9A). We subsequently differentiated control and VPS35-deficient hiPSCs into liver organoids. The success of the differentiation process was confirmed by analyzing mRNA levels of pluripotency and hepatocyte markers (Fig. S9B and C). We then assessed NPC1 and SCARB2 expression by immunoblotting and found that the loss of VPS35 in hiPSC-derived liver organoids significantly decreased the expression of these lysosomal proteins (Figure 6F), without affecting their mRNA levels (Fig. S9D). We were unable to study the protein levels of LAL due to the lack of commercially available antibodies specific to LAL, and LAL was not detectable by using mass spectrometry in these organoids. In summary, and consistent with our findings in mouse livers, human VPS35 also plays an essential role in maintaining the levels of NPC1 and SCARB2 in hepatocytes.

### 2.10 Reduced NPC1 and SCARB2 protein levels in *Vps35*^HepKO^ livers are not due to protein misfolding, increased degradation, enhanced secretion, or mislocalization

To get mechanistic insight into how VPS35 loss leads to a decrease in NPC1 and SCARB2 levels, we studied cultures of primary hepatocytes isolated from *Vps35*^HepKO^ and control mice. We first confirmed that VPS35 deficiency reduces NPC1 and SCARB2 levels (Figure 8A), a finding that again cannot be explained by their mRNA levels (Fig. S10A). A previous study showed that NPC1 can be degraded when it fails to be transported from the ER to late endosomes/lysosomes or is misfolded [40]. NPC1 mutant proteins are degraded via ER-phagy, the selective ER degradation by autophagy, or ER-associated degradation (ERAD) [41]. To determine whether VPS35 loss affects protein folding, we cultured primary hepatocytes from *Vps35*^HepKO^ and control mice at 30 °C to improve the quality of protein folding [42]. However, lowering the temperature to 30 °C did not increase NPC1 and SCARB2 protein expression compared to cells cultured at 37 °C (Figure 8B).

**Figure 8:**
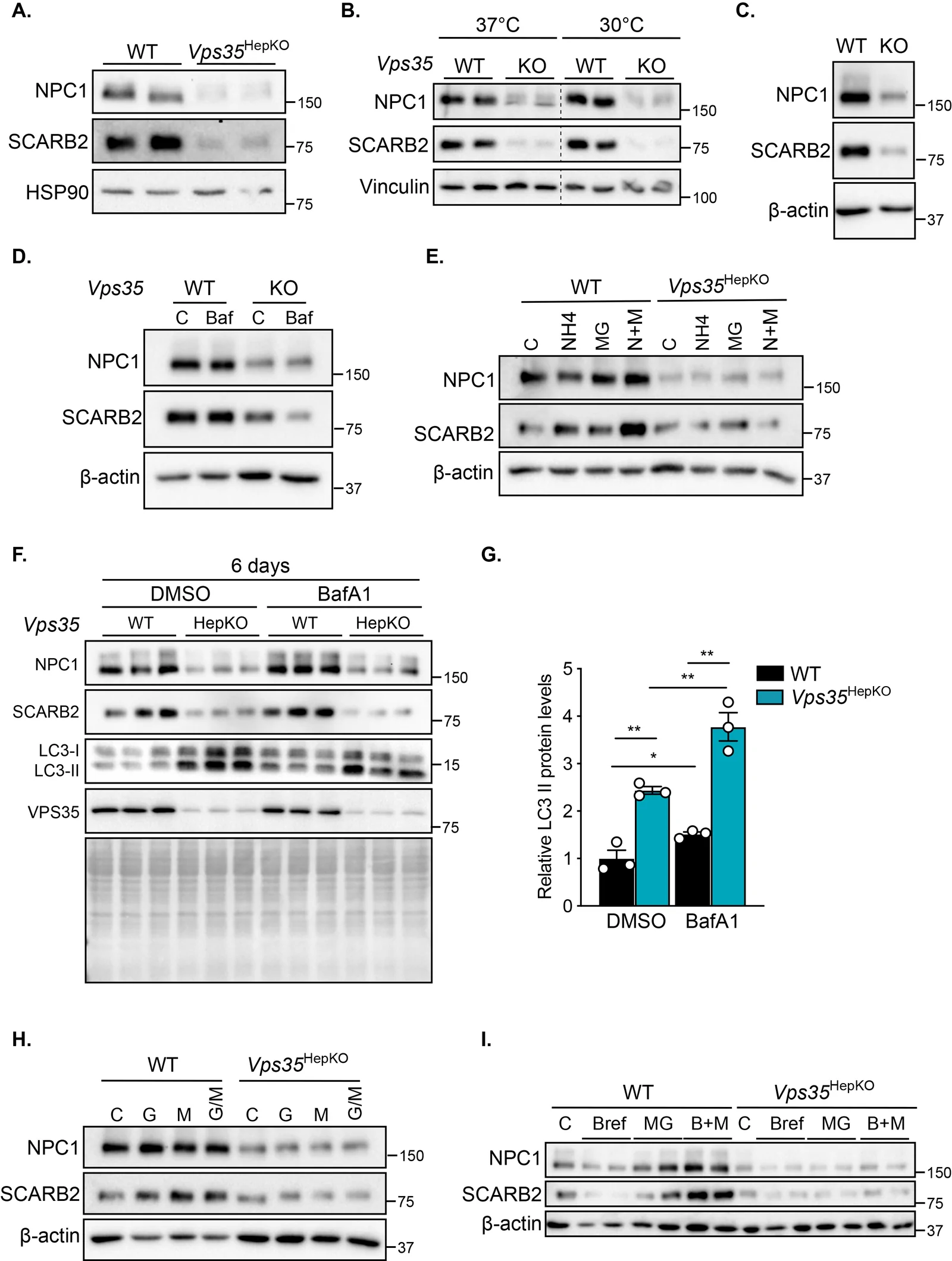
**Reduced NPC1 and SCARB2 levels in *Vps35*^HepKO^ mice are not caused by protein misfolding, enhanced proteolysis, increased exosome release, or defects in the post-ER-Golgi trafficking pathway.** (**A**) Protein levels of NPC1 and SCARB2 in primary hepatocytes isolated from WT and *Vps35*^HepKO^ mice (n = 2) fed a standard laboratory diet. (**B**) Immunoblot of NPC1 and SCARB2 in WT and VPS35-deficient primary hepatocytes cultured at 37 °C or 30 °C. (**C**) Immunoblot of NPC1 and SCARB2 in precision-cut liver slices (PCLS) of WT and *Vps35*^HepKO^ mice, untreated or treated with (**D**) DMSO (C) or 100 nM Bafilomycin A1, (**E**) DMSO (C), 2.5 mM ammonium chloride (NH4), 5 μM MG132 (MG), or 2.5 mM NH4 + 5 μM MG for 16 h. (**F**) WT and liver-specific VPS35-deficient mice were injected with DMSO or Bafilomycin A1 (BafA1) for 6 consecutive days, and the expression of the indicated proteins was determined by immunoblotting (n = 3). (**G**) Relative LC3 levels in livers from WT and *Vps35*^HepKO^ mice as determined in (G). (**H**) Immunoblot of NPC1 and SCARB2 in PCLS of WT and *Vps35*^HepKO^ mice treated with DMSO (C), 5 μM GW4869 (GW), 250 nM manumycin A1 (M), or 5 μM GW + 250 nM M (G/M) for 16 h, (**I**) 10 μg/mL Brefeldin A (Bref) (refreshing every 6 h), 2.5 μM MG or 10 μg/mL Bref (refreshing every 6 h) + 2.5 μM MG for 20 h.

To assess whether hepatic VPS35 deficiency promotes the proteolysis of NPC1 and SCARB2, we treated precision-cut liver slices (PCLS) from *Vps35*^HepKO^ and control mice with lysosomal inhibitors bafilomycin A1 (BafA1) or NH_4_Cl, and/or the proteasomal inhibitor MG132 for 16 h. We confirmed that VPS35 deficiency markedly reduces SCARB2 and NPC1 protein levels in PCLS cultured for 16h (Figure 8C). However, the inhibitors that block protein proteolysis could not rescue the strong reduction in NPC1 and SCARB2 levels (Figure 8D,E). In contrast, the accumulation of ubiquitinated proteins was markedly increased in controls and *Vps35*^HepKO^ liver slices upon this treatment (Fig. S10B and C), indicating that the inhibitors used were effective in blocking the proteolysis of proteins under these conditions.

The half-lives of NPC1 and SCARB2 can exceed 42h [40], [43]. As treating PCLS with BafA1 for more than 24h can be toxic to the *ex vivo* tissue, we treated *Vps35*^HepKO^ and control mice with BafA1 (1 mg/kg/day) for 6 consecutive days. Blocking lysosomal degradation did not rescue the decreased levels of NPC1 and SCARB2 (Figure 8F). In *Vps35*^HepKO^ mice, we also found elevated LC3-II levels compared to controls (Figure 8F,G). LC3 levels increased in *Vps35*^HepKO^ and control livers after BafA1 treatment, suggesting successful lysosomal inhibition (Figure 8F,G).

Next, we determined whether loss of VPS35 enhances the extracellular release of NPC1 and SCARB2 through exosomes, as lysosomal dysfunction can lead to an increase in exosome release[44], [45], [46], [47], [48]. We treated the liver slices with GW4869 and/or manumycin A, which block exosome biogenesis and release [49]. GW4869 inhibits ESCRT-independent exosome release, and manumycin inhibits ESCRT-dependent exosome release. These two drugs also did not reverse the reduction of NPC1 and SCARB2 levels in *Vps35*^HepKO^ liver slices (Figure 8H).

A recent study reported that loss of VPS35 in neuronal cells increases NPC1 and SCARB2 at the cell surface [38]. To assess whether this also occurs in livers, we measured NPC1 and SCARB2 levels at the PM. However, in contrast to the previous study [38], we found decreased NPC1 and SCARB2 levels at the PM of VPS35-deficient liver cells compared with WT controls (Fig. S10D).

To assess whether VPS35 deficiency affects NPC1 and SCARB2 post-ER trafficking, we treated control and *Vps35*^HepKO^ liver slices with Brefeldin A, an inhibitor that blocks protein trafficking between the ER and Golgi apparatus. Brefeldin A decreased NPC1 and SCARB1 levels in both WT and *Vps35*^HepKO^ liver slices (Figure 8I), likely due to an augmentation of ERAD [41]. This decrease in protein levels was normalized to the levels observed in untreated *Vps35*^HepKO^ liver slices by blocking proteasomal activity with MG132. In control liver slices, however, the levels of these two proteins were even increased after MG132 treatment (Figure 8I). These results indicate that the loss of VPS35 does not adversely affect post-ER trafficking.

As mRNA levels, protein degradation, secretion, surface localization and post-ER trafficking were all unaffected, reduced synthesis remained the most likely explanation for the loss of NPC1 and SCARB2 in *Vps35*^HepKO^ mice. We therefore attempted to measure protein synthesis directly using a click chemistry-based pulse-chase experiment [50]. We focused on SCARB2 as a representative lysosomal protein to elucidate the mechanism, since our antibody for immunoblotting to detect NPC1 is not suitable for immunoprecipitation assays. We performed AHA-based metabolic labelling in primary hepatocytes. Although global incorporation was robust (Fig. S11), we could not recover newly synthesized SCARB2 by immunoprecipitation, precluding measurement of its synthesis and turnover.

Taken together, these data suggest that the reduced levels of NPC1 and SCARB2 following VPS35 ablation are not caused by decreased mRNA levels, protein misfolding, enhanced proteolysis, increased exosome release, mislocalization to the cell surface, or defects in the post-ER-Golgi trafficking pathway.

## 3 Discussion

Retromer is a central player in the endo-lysosomal network that facilitates the transport of cargos from endosomes back to the trans-Golgi network (TGN) or the PM, protecting them from lysosomal proteolysis[16], [17], [18], [19]. Retromer dysfunction has been linked to neurodegenerative diseases such as Alzheimer's (AD) and Parkinson's disease (PD)[51], [52], [53], and several studies have implicated retromer in cholesterol homeostasis [20], [23]. However, its role in systemic cholesterol homeostasis remained unclear. To our knowledge, this is the first study showing that hepatic retromer is essential for maintaining systemic cholesterol homeostasis. We show that retromer is essential for receptor-mediated hepatic uptake of LDL cholesterol and for intracellular cholesterol transport through the endo-lysosomal system by maintaining the lysosomal levels of key lysosomal cholesterol-handling proteins, including LAL, NPC1, NPC2 and SCARB2.

Our study reveals that *Vps35*^HepKO^ mice exhibit elevated plasma LDL cholesterol levels. In previous work, we showed that WASH and CCC, two multimeric protein complexes associated with retromer, coordinate the endosomal recycling of LDLR and LRP1 [9], [10], [11]. This study suggests that retromer is also essential for endosomal trafficking of LDLR and LRP1 from endosomes to the PM, where they contribute to the hepatic uptake of atherogenic lipoproteins, including LDL and triglyceride-rich lipoprotein remnants. Retromer is known to recruit both the WASH and CCC complexes onto endosomes [25], [54], [55], however, we did not observe any adverse effect on the levels or subcellular distribution of WASH and CCC components in *Vps35*^HepKO^ livers. These data suggest that the elevated LDL cholesterol in *Vps35*^HepKO^ mice is independent of WASH and CCC. Others have also shown this independent role for retromer in CCC/WASH-mediated endosomal cargo sorting [24], [25]. Furthermore, total LDLR levels were increased in *Vps35*^HepKO^ mice, while its abundance at the plasma membrane remained unchanged, a phenotype we did not observe upon CCC or WASH ablation [9], [10], [11].

Although we cannot exclude that LDLR also accumulates in other cellular compartments after VPS35 deletion, our data suggest that LDLR accumulates in lysosomal compartments in VPS35-deficient livers. These data may suggest that retromer-mediated LDLR transport is impaired in VPS35-deficient livers. However, the reduced hepatic LDL cholesterol uptake could also be explained by impaired LDLR internalization, as plasma membrane cholesterol content has been shown to modulate the dynamics of LDLR trafficking [56], [57]. Notably, increased cholesterol levels may occur not only in lysosomes but also in the plasma membrane, thereby impeding LDLR internalization [56]. We also speculate that the increase in LDLR levels in livers represents a compensatory response to reduced LRP1 function in VPS35-deficient livers, consistent with previous findings [31], [58], [59]. Taken together, our data suggest that impaired lipoprotein receptor-mediated hepatic LDL cholesterol uptake, combined with increased hepatic cholesterol synthesis in *Vps35*^HepKO^ mice, causes the elevated plasma LDL cholesterol in *Vps35*^HepKO^ mice.

Unraveling the subcellular cholesterol accumulation in VPS35-deficient hepatocytes provided insight into how loss of VPS35 increases hepatic cholesterol content and *de novo* cholesterol synthesis. We found that FC mainly accumulated within LAMP2-positive lysosomal compartments, consistent with marked reductions in the lysosomal cholesterol-handling proteins NPC1, NPC2, and SCARB2, resulting in impaired lysosomal cholesterol egress and, subsequently, increased *de novo* cholesterol synthesis [60], [61], [62]. Notably, CE accumulated in PLIN3-positive lipid droplets, and not in lysosomes, suggesting that these cholesterol pools have distinct origins. Residual LAL activity in VPS35-deficient livers is likely sufficient to hydrolyze most of the endocytosed CE into FC. CE found in lipid droplets is therefore likely derived from excess cholesterol generated through the upregulated SREBP2-mediated *de novo* cholesterol synthesis pathway and esterified by sterol O-acyltransferase (SOAT) enzymes in the ER to be stored in lipid droplets and/or packaged into very-low-density lipoprotein (VLDL) particles [63].

VPS35 deficiency also profoundly altered lysosomal morphology, proteome, and function. The number of MVBs and degradative compartments was significantly increased, while many luminal enzymes (e.g., cathepsins, glycosidases, and sulfatases) were decreased in VPS35-deficient hepatocytes. Furthermore, we found that late endosomal/lysosomal pH was significantly more acidic in VPS35-deficient hepatocytes than in controls. Quantitative late endosomal/lysosomal pH measurements in live hepatocytes are scarce, however, our control primary mouse hepatocyte values are consistent with those reported in HepG2 cells using LysoSensor-conjugated dextrans (pH ∼5.2) [64] and align with the notion that hepatocyte lysosomes may be less acidic than those of other cell types, such as macrophages (pH 4.9–5.1) [39]. Although the functional consequences of this increased acidification remain to be established, it likely contributes to the altered lysosomal cargo processing observed in VPS35-deficient livers, as shown in other lysosomal storage disorders associated with reduced lysosomal pH [65], [66]. Furthermore, the perinuclear redistribution of late endosomes/lysosomes observed in VPS35-deficient hepatocytes is consistent with cholesterol-loaded lysosomes in NPC deficiency [67]. Altogether, these data strongly suggest that VPS35 ablation impairs lysosomal function.

The mechanism by which VPS35 depletion causes a marked reduction of lysosomal proteins, and whether this is the same for all of them, remains unclear. LAL is a lysosomal luminal protein, and modification of LAL with mannose-6-phosphate (M6P) in the TGN is required for its efficient targeting to lysosomes, as for many soluble lysosomal proteins, including NPC2 and cathepsins [68]. Retromer mediates the retrograde transport of CI-M6PR from the endosome back to the TGN [21], [22], and since CI-M6PR is one of the two receptors responsible for the transport of M6P-modified proteins to the lysosomes [69], loss of VPS35 may indirectly impair the transport of LAL from the TGN to the lysosomes. This defect in LAL sorting could increase extracellular LAL secretion, a phenomenon previously observed for cathepsin D[26], [70], [71], [72]. In addition to NPC2 and LAL, we found that several soluble lysosomal proteins that rely on the M6P pathway to reach the lysosomes (e.g., cathepsins, glycosidases, and sulfatases) were reduced in VPS35-deficient lysosomes. Although the lysosomal levels of CI-M6PR (IGF2R) and cation-independent M6PR (M6PR), the two receptors transporting the M6P-tagged lysosomal enzymes to the lysosomes, remained unchanged (Figure 7E), these data imply that VPS35 deficiency indirectly impairs the transport of these specific lysosomal proteins to the lysosomes, resulting in increased extracellular secretion of newly synthesized lysosomal enzymes. Interestingly, however, we found that total NPC2 levels were increased while lysosomal levels were decreased, which might indicate that newly synthesized NPC2 accumulates in specific cellular compartments and does not reach the lysosomes. Further studies are needed to elucidate the underlying mechanism.

Enhanced lysosomal exocytosis and exosome release are commonly observed under conditions of lysosomal dysfunction to protect cells and maintain cellular homeostasis[44], [45], [46], [47], [48]; because VPS35 deficiency causes lysosomal dysfunction, the reduction in NPC1 and SCARB2 could be due to increased exocytosis. However, inhibiting exocytosis failed to restore NPC1 and SCARB2 levels, excluding this pathway. However, these results should be interpreted with caution, as these compounds might act differently in VPS35-deficient cells.

Interestingly, blocking ER-to-Golgi and Golgi-to-PM transport with brefeldin A further decreased NPC1 and SCARB2 levels in both WT and VPS35-deficient liver slices, likely due to enhanced degradation via the ERAD system. This assumption is supported by experiments in which brefeldin A combined with the proteasomal inhibitor MG132 restored protein levels to those observed in untreated WT and VPS35-deficient liver slices. Notably, while dual inhibition led to even higher protein levels in WT livers compared to untreated controls, this increase was not observed in VPS35-deficient liver tissue. Together with the observation that *Npc1* and *Scarb2* mRNA levels were not negatively affected in hepatic VPS35-deficient mice, it is plausible that retromer deficiency impairs NPC1 and SCARB2 translation. Interestingly, a recent study reported a novel link between VPS35 expression and translation. Here, they showed that loss of VPS35 reduces mitochondrial translation in HEK293T and lymphoblast cells by regulating the expression of the cationic amino acid transporter SLC7A1 at the plasma membrane [73]. Since SLC7A1 is not expressed in the liver, it may be possible that VPS35 regulates the translation of these lysosomal proteins through an alternative, yet unidentified, mechanism in hepatocytes.

Using H4 neuroglioma cells, Daly and colleagues also found a critical role for retromer in the endo-lysosomal system [38]. Similar to our study, they showed that retromer dysfunction causes morphological changes in the endo-lysosomal network, accompanied by marked changes in the lysosomal proteome, including lysosomal membrane and luminal proteins such as SCARB2 and LAL. In contrast, they did not find reduced total or lysosomal NPC1 levels, but they showed markedly increased surface NPC1 levels, which they explained as increased lysosomal exocytosis to compensate for lysosomal stress. In our model system, NPC1 and SCARB2 levels were even lower at the cell surface than in controls and were not rescued after inhibiting exocytosis. Consistent with Daly's study, proteins involved in autophagy and mTOR signaling were strongly reduced in VPS35-deficient lysosomes. Although VPS35 deficiency increases LC3-II levels, autophagic flux remains present in VPS35-deficient hepatocytes, which is consistent with previous work [26], [38]. However, whether mTOR signaling and mTOR-mediated autophagy initiation are also affected in VPS35-deficient livers, as shown by Daly and others in H4 and HeLa cells [26], [38], remains to be determined.

In addition, consistent with Daly's study, we observed increased lysosomal RAB7A, despite decreased whole-liver RAB7A upon VPS35 ablation, indicating RAB7A redistribution toward the lysosomes. Jimenes-Ogaz and colleagues have also described this phenomenon. They showed that RAB7A can localize to the ER, TGN, and mitochondria in HeLa cells, but upon VPS35 removal, it is mainly localized to late endosomes/lysosomes in an active form. Interestingly, active RAB7A promotes lysosomal cholesterol export, including in NPC1-deficient cells[74], [75], [76], [77], suggesting that the increased lysosomal RAB7A in VPS35-deficient liver might counteract cholesterol accumulation. However, this increase is insufficient to prevent substantial free cholesterol accumulation in lysosomes, perhaps because RAB7A-mediated NPC1-independent cholesterol export has mainly been observed under RAB7A overexpression or activation conditions, which likely exceed the levels and activity in VPS35-deficient livers.

Although we ruled out several candidate pathways, the mechanism by which VPS35 loss reduces NPC1 and SCARB2 levels remains unclear, which we identify as a limitation of our study. Nonetheless, our findings provide novel physiological insight into how hepatic VPS35 deficiency disrupts systemic cholesterol homeostasis and lay the groundwork for future mechanistic work. Furthermore, we found an interesting sex-specific effect of VPS35 deficiency on cholesterol levels. Although NPC1, SCARB2, and LAL levels were reduced in both male and female mice, an increase in hepatic CE content and plasma cholesterol levels was observed only in *Vps35*^HepKO^ male mice, not in female mice. Although cholesterol metabolism differs between male and female mice [78], we currently don't have an explanation for these sex-specific effects, and future in-depth comparisons between male and female mice are warranted. We also found that not all SREBP2-target genes were significantly increased, including *Ldlr*, while *de novo* cholesterol synthesis was increased. This likely reflects that total cholesterol biosynthesis was relatively modest, suggesting that SREBP2 activation was likewise subtle, which may partly explain why we do not observe a corresponding increase in *Ldlr* mRNA at the time we collected the liver samples. We would also note that expression of SREBP2 target genes varies during the day [79], whereas our cholesterol flux measurements integrate biosynthesis over a longer time window. This difference in temporal resolution between a single-timepoint mRNA snapshot and an integrated flux measurement may further contribute to the apparent discordance between the transcriptional and biosynthetic readouts. In addition, we found that body and liver weights differed between *Vps35*^HepKO^ and control mice. We recently showed that liver-specific VPS35 deletion also increased hepatocellular proliferation in postnatal livers, which likely explains the difference in liver weight [80]. However, the cause of the body weight difference requires further study.

Taken together, our findings reveal that hepatic retromer, in addition to its well-established role in endosomal sorting of cargos, plays a dual role in cholesterol homeostasis: maintaining the endosomal transport of lipoprotein receptors LDLR and LRP1, and preserving the lysosomal levels of key cholesterol-handling proteins, including LAL, NPC1, NPC2, and SCARB2. Given the links between retromer dysfunction and neurodegenerative disease, our findings also raise the possibility of a role for impaired lysosomal cholesterol handling in the pathology of AD and PD, opening new avenues for investigation.

## 4 Materials and methods

### 4.1 Animals

Mice carrying a *Vps35* allele flanked with *LoxP* sites (*Vps35*^fl/fl^; 129/Ola background) [81] were crossed with a transgenic mouse line (C57Bl/6J background) expressing Cre recombinase under the control of the *Albumin* (*Alb*) gene promoter (#003574, The Jackson Laboratory), to generate a liver-specific *Vps35* knockout (*Vps35*^HepKO^) mouse model. In the case of somatic ablation of *Vps35* in mouse livers, we used a CRISPR-mediated gene editing approach as described previously [29]. The sequence of *Vps35* sgRNA's used is indicated in Fig. S4A. To ablate *Vps35* in a *Ldlr* knockout background, mice expressing Cas9 specifically in the liver (#026556, The Jackson Laboratory) [82] were crossed with *Ldlr* knockout mice (C57Bl/6J background) (#002207, The Jackson Laboratory). Conditional LysoTag mice (#033065, The Jackson Laboratory) were crossed with mice expressing Cas9 and Cre recombinase in the liver to achieve hepatocyte-specific expression of both TMEM192-3 × HA and Cas9. To delete hepatic *Vps35*, mice were injected with AAV encoding sgRNAs targeting Vps35 as described above.

All animal experiments were approved by The Central Authority for Scientific Procedures on Animals (CCD), as well as by the Animal Welfare Body (IvD) of the University of Groningen (Groningen, the Netherlands). Male and female mice were used for the experiments as indicated in the results section. Mice were individually housed under a 12h light–dark cycle and were fed *ad libitum* with standard laboratory diet (V1554-703, Ssniff Spezialdiäten GmbH).

Mice were killed after 4 h of fasting. Blood was collected through cardiac puncture under anesthesia in EDTA-coated tubes. Plasma was collected after centrifugation at 1000×*g* for 10 min at 4 °C. Liver tissues were harvested and either snap-frozen in liquid nitrogen and stored at -80 °C until further analysis, or fixed in 4% paraformaldehyde (PFA), embedded in paraffin, and cut into 4 μm sections for histological analysis.

### 4.2 Cholesterol flux measurements

Cholesterol fluxes were measured using a dual stable isotope tracer method as previously described [32] To determine intestinal cholesterol absorption, mice were administered cholesterol-D_5_ and cholesterol-D_7_, as follows: 0.3 mg cholesterol-D_5_ (Medical Isotopes Inc., Pelham, NH) was dissolved in 150 μL 20% Intralipid (Fresenius Kabi, Den Bosch, the Netherlands) and was injected intravenously. 0.6 mg cholesterol-D_7_ (Cambridge Isotope Laboratories, Inc., Andover, MA) was dissolved in 200 μL medium-chain TG oil and was administered by oral gavage. Blood spots were collected from the tail vein prior to and at 3, 6, 9, 12, 24, 36, 48, 60, 72, 96, 120, 144, and 168 h after administration for measurement of tracer enrichments. Standard pharmacokinetics were applied for fitting the enrichment curves using SAAM-II software (version 2.3, The Epsilon Group, Charlottesville, VA). The formula used for curve fitting was *f (t)* = *f*_*1*_ *e*^*-k1t*^ + *f*_*2*_ *e*^*-k2t*^ – *f*_*3*_ *e*^*-k3t*^.

To assess whole-body cholesterol synthesis using mass isotopomer distribution analysis (MIDA) [33], [34], mice received drinking water containing 2% [1-^13^C] acetate and bloodspots were collected at 0, 24, 33, 48, 57, 72, 81, 96, 105, and 120 h. After the final time point, mice were sacrificed, and livers were collected for biochemical characterization.

### 4.3 Hepatic LDL uptake assay

LDL and lipoprotein-deficient serum (LPDS) were collected after density gradient ultracentrifugation of human serum. Both fractions were dialyzed overnight against PBS. Liposomes were generated by sonication of 2 mg egg yolk phosphatidylcholine and 40 μCi of [^14^C]cholesteryl oleate in 1 mL 50 mM Tris/HCl pH 7.4. The LDL fraction (6 mL containing approximately 10 mg cholesterol) was incubated with 4.5 mL LPDS, 0.5 mL liposomes and 0.1 mL 0.2 M EDTA pH 7.4 overnight at 37 °C. After radiolabelling, LDL was isolated again using density gradient ultracentrifugation and the fraction was dialyzed against PBS.

Mice were intravenously injected with [^14^C]cholesteryl oleate-labelled LDL (0.33 mg cholesterol in 200 μL saline per mouse) and sacrificed 120 min later. Collected blood was centrifuged to obtain plasma. The animals were subsequently perfused with ice-cold saline before the liver was collected. A piece of liver was dissolved overnight at 55 °C in Solvable (PerkinElmer). Samples were mixed with Ultima Gold liquid scintillation cocktail (PerkinElmer) and scintillation counting was performed in a Tri-Carb 2910 TR low-activity liquid scintillation analyzer (PerkinElmer).

### 4.4 Histological analysis

Paraffin-embedded liver samples were cut into sections of 4 μm, which were stained with hematoxylin-eosin (H&E). Cell death was assessed by Terminal deoxynucleotidyl transferase-mediated dUTP nick-end labeling (TUNEL; S7100, Merck Millipore). Images were obtained with a Leica DM3000 microscope with a mounted Leica DFC420 camera. To quantify cell death, the number of TUNEL-positive cells was counted in ten high-power fields with 20× magnification for each liver sample of each experimental group. For each sample, the average of positive cells per field was calculated, which was used to calculate the average per field per experimental group.

### 4.5 Scanning transmission electron microscopy (TEM)

For TEM analysis, small pieces of fresh liver (1–2 mm^3^) were fixed in 2% PFA and 2% glutaraldehyde in 0.1 M sodium cacodylate buffer (pH 7.4) at 4 °C. Tissue processing, including staining with neodymium, sectioning, and image acquisition, was performed as previously described [83], [84]. The images of the preparations are available upon request.

### 4.6 Hepatic lipid extraction

Liver homogenates (15% w/v in PBS) were used for lipid extraction according to Bligh and Dyer [85]. 200 μL of liver homogenate was added to 600 μL of demi-water, mixed with 3 mL chloroform/methanol (1:2 v/v) in a glass tube, and incubated for 30 min. Then, 1.2 mL of water and 1 mL of chloroform were added, mixed, and samples were centrifuged at 500×*g* for 10 min at room temperature (RT). The organic layer was transferred to a new glass tube, and the solvent was evaporated with nitrogen at 50 °C. The dried lipids were dissolved in 1 mL chloroform, from which 400 μL was again dried and dissolved in 500 μL of 2% Triton X-100 in chloroform, and evaporated with nitrogen at 50 °C. Then, 500 μL of water was added, and samples were incubated for 15 min at 37 °C before further analysis for cholesterol and TG concentrations.

### 4.7 Lipid measurements in plasma and liver

Colorimetric assays were used to determine total cholesterol (TC), FC, TG, and phospholipid concentrations. Cholesterol enzymatic reagents were used to determine TC (#11489232, Roche; Cholesterol FS, DiaSys) and FC concentrations (#113609910930, DiaSys), with the cholesterol standard FS (DiaSys) as a reference. TG levels were determined with the Trig/GB kit (#1187771, Roche) or Triglycerides FS (DiaSys), using the Precimat Glycerol standard (Roche) as a reference.

Lipidomic analyses of liver samples were performed using the Lipidyzer™ Platform (SCIEX, Framingham, MA, USA), as previously described [86].

### 4.8 Fast protein liquid chromatography (FPLC)

TC and TG content of the major lipoprotein classes (VLDL, LDL, and HDL) was determined using FPLC analysis as previously described [29]. Briefly, measurements were performed with a system containing a PU-4180 RHPLC pump and a UV-4075 UV-Vis detector (Jasco). Measurements were done in individual plasma samples when enough sample was available, or in pooled plasma samples as indicated. The samples, diluted in PBS, were loaded onto a Superose™ 6 Increase 10/300 GL column (GE Healthcare) for separation of lipoproteins at a flow rate of 0.31 mL/min. An enzymatic reagent for cholesterol (Roche) was added through a second flow at a flow rate of 0.1 mL/min.

### 4.9 Liver enzyme measurements in plasma

Concentrations of alanine aminotransferase (ALT) and aspartate aminotransferase (AST) in plasma were measured with a clinical chemistry analyzer (Cobas 6000, Roche Diagnostics), using standard reagents (Roche Diagnostics).

### 4.10 Lysosomal immunoprecipitation (LysoIP)

LysoIP was performed as described by Abu-Remaileh et al. and Laqtom et al. [30], [87] with adaptations. Immediately after sacrifice, a 5 mm biopsy punch was taken from the large liver lobe and homogenized fresh (without freezing) in 1 mL ice-cold PBS supplemented with protease and phosphatase inhibitor cocktails using a 2 mL dounce homogenizer (25 strokes). Protein concentration was determined by Bradford assay and homogenates were normalized to 4.5 mg/mL. The homogenate was centrifuged at 1,000×*g* for 2 min at 4 °C and the post-nuclear supernatant was incubated with 120 μL pre-washed anti-HA magnetic beads for 20 min at 4 °C on a gentle rotator. Beads were washed three times with 1 mL PBS, with the tube changed during the final wash. Immunoprecipitated lysosomes were resuspended in 100 μL Triton lysis buffer (40 mM HEPES pH 7.4, 1% Triton X-100, 10 mM β-glycerophosphate, 10 mM sodium pyrophosphate, 2.5 mM MgCl_2_, protease and phosphatase inhibitors) and incubated for 10 min at 4 °C. For immunoblotting, 10 μg of input (0.2% of total) and 20 μL of the lysosomal fraction (15% of total) were resolved by SDS-PAGE as described below. For proteomics, the lysosomal fraction was processed for mass spectrometry as described below. For analysis in R (v4.x), proteins were required to be quantified in at least three of five replicates in both experimental groups. Intensities were log2-transformed and, for each protein, the mean of the three negative-controls was compared to the mean of the experimental samples. Proteins were retained when they were at least 1.2-fold enriched over the negative-control mean; proteins without any negative-control signal were retained by default. Retained proteins were median-normalized across samples using global median scaling.

### 4.11 Sucrose gradients

A 20% w/v homogenate was prepared with a piece of snap-frozen liver tissue in homogenization buffer (50 mM Tris-HCl [pH 7.4], 250 mM sucrose, 25 mM KCl, 5 mM MgCl_2_, 3 mM imidazole, supplemented with protease inhibitors [Roche]). To remove nuclei and debris, homogenates were centrifuged at 1000×*g* for 10 min at 4 °C. Protein concentrations were determined using the Bradford method (Bio-Rad). 1.5 mg of liver homogenate was loaded onto a 3.7 mL continuous 10%–40% sucrose gradient, which was centrifuged for 16 h at 40,000 rpm in a Beckman Colter SW55 Ti rotor. For further analysis with untargeted proteomics, 30 μL of each fraction was mixed with 10 μL of 4*x* LDS sample buffer.

### 4.12 Cell culture

VPS35-deficient cells were generated in a Cas9-expressing Hepa1-6 murine hepatoma cell line (kindly provided by Noam Zelcer [88]), as previously described [29]. Adenovirus to express sgRNAs to target *Vps35* was generated as previously reported [9]; sgRNA sequences are listed in Supplemental Table 1. The cells were cultured in Dulbecco's modified Eagle medium (DMEM) GlutaMAX (Gibco), supplemented with 10% fetal calf serum, 1% penicillin-streptomycin, and 1.25 μg/mL puromycin, at 37 °C and 5% CO_2_. Subsequently, cells were used to assess LAL activity (further described below).

### 4.13 Precision-cut liver slices

Precision-cut liver slices (PCLS) were prepared from mouse liver biopsy cores obtained using a 6 mm punch tool (KAI Medical), following the general methodology outlined by Groothuis et al. [89]. Liver tissue sections, approximately 250–300 μm thick and weighing ∼5 mg, were generated with a Krumdieck Tissue Slicer (Alabama Research and Development). Each slice was individually placed in a well of a 12-wells plate containing Williams E medium (US Biological Life Sciences, C17082259), supplemented with 25 nM glucose and 50 μg/mL gentamycin (Invitrogen). Cultures were maintained at 37 °C under continuous agitation (90 rpm) and exposed to an 80% O_2_ /5% CO_2_ atmosphere for up to 24 h*.* Liver slices were treated with 25 nM bafilomycin A1 or 2.5 mM ammonium chloride for 16 h to inhibit lysosomal degradation. To suppress the formation and release of extracellular vesicles and exosomes, PCLS were treated with 5 μM GW4869 or 250 nM manumycin A1 for 16 h. To block proteasomal protein degradation, PCLS were treated with 5 μM or 2.5 μM MG132 for 16 or 22 h, respectively. Brefeldin A at a concentration of 10 μg/mL for 22 h, refreshed every 6 h, was used to inhibit the transport of proteins from the ER to the Golgi apparatus. After each respective incubation period, three liver tissue slices for each experimental condition were pooled in a single 1.5 mL microcentrifuge tube. Subsequently, protein extraction was performed as described below.

### 4.14 Genome editing of human induced pluripotent stem cells (hiPSCs) and genotyping

The *VPS35*^−/−^ hiPS cell line was derived from the genomic editing of a control hiPS cell line reprogrammed from urinary progenitor cells and previously characterized [90], [91]. Genomic editing was performed using the Alt-R™ CRISPR-Cas9 system (Integrated DNA Technologies) following a described procedure [92]. Two CRISPR guide RNAs (TACGAACTTGTACAGTATGC and GGTGTGCAACATCCCTTGAG) targeting exons 4 and 5 of *VPS35,* respectively, were used to delete 655 bp. After genome editing and clonal selection, hiPSC clones were genotyped by PCR and Sanger sequencing. Primers are listed in Supplemental Table 2.

### 4.15 hiPSCs culture and differentiation into liver organoids

hiPSCs were cultured under hypoxia (4% O_2_, 5% CO_2_) on plates coated with 0.05 mg/mL Matrigel® (Corning) in StemMACS™ iPS-Brew medium XF (Miltenyi) and passaged using the Gentle Dissociation reagent (Stem Cell Technologies). In-suspension liver organoids were generated following a modified protocol previously described [93]. hiPSCs were first dissociated and seeded in 24-wells AggreWell™400 plates (Stem Cell Technologies) at 1000 cells/well as recommended by the manufacturer, in StemMACS™ iPS-Brew XF medium supplemented with 10 μM Y27632 (Cell Guidance Systems) to form aggregates (day -2). The next day, the media was carefully changed using StemMACS™ iPS-Brew medium XF without disturbing the aggregates. On day 0, aggregates were collected using a 37 μm reversible strainer (Stem Cell Technologies) and washed with complete RPMI 1640 media supplemented with 1*X* insulin containing B27, 1*X* NEAA and 1*X* GlutaMAX™-I (Life Technologies). The aggregates were further resuspended in a vented Erlenmeyer flask in complete RPMI medium supplemented with 4 μM CHIR99021 (BOC Sciences) to induce mesendoderm formation and incubated at 37 °C under agitation. All subsequent steps were performed under orbital shaking at 70 rpm. On day 1 after initiation of the differentiation, the media was changed to complete RPMI medium without any other molecule to pattern aggregates towards definitive endoderm. From day 2–6, aggregates were directed towards hepatic endoderm with medium changes every 2 days using knockout DMEM supplemented with 20% Knockout serum replacement (KSR), 1*X* NEAA, 1*X* GlutaMAX™-I (Life Technologies) and 1% DMSO and 100 μM 2-mercaptoethanol (Sigma). From day 7–19, liver organoids were cultured in liver maturation medium with medium changes every 2 to 3 d using hepatocyte culture medium (HCM) without EGF (Lonza) supplemented with 100 nM dexamethasone (Sigma) and 100 nM N-hexanoic-Tyr-Ile-(6) aminohexanoic amide (Active Peptide). Organoids were harvested on day 20 for further analysis. Cellular pluripotency was assessed by measuring the expression of *OCT4*, *NANOG*, *SOX2 and ALB, HNF4, AFP* as liver-like specific markers (Supplemental Table 2). RNA was extracted from hiPSCs and liver organoids using the NucleoSpin Tissue Purification Kit (Macherey–Nagel). Reverse transcription of RNA to cDNA was performed using the high-capacity cDNA reverse-transcription kit (Applied Biosystems). qPCR studies were performed in triplicate for hiPSCs and quadruplicate for liver organoids using the Power SYBR® Green PCR Master Mix on the QuantStudio^5^ system with automatic threshold calculation (Applied Biosystems™).

### 4.16 Isolation of liver plasma membrane

Subcellular fractionation was obtained by using the Mem-PER Plus Membrane Protein Extraction kit (Life Technologies). Liver tissue weighing 20–40 mg was placed in a 5 mL microcentrifuge tube. 4 mL of cell wash solution was added to the tube, followed by a brief vortex. The wash solution was discarded. The tissue was transferred to a 2 mL tissue grinder. 1 mL of permeabilization buffer was added, and the tissue was homogenized with 6–10 strokes until an even suspension was achieved. An additional 1 mL of permeabilization buffer was added to the homogenate, which was then transferred to a new tube and incubated at 4 °C for 10 min with constant mixing. The permeabilized suspension was centrifuged at 16,000×*g* for 15 min at 4 °C to obtain a pellet. The supernatant containing the cytoplasmic fraction was removed and transferred to a new tube. The pellet was resuspended in 1 mL of solubilization buffer by pipetting up and down to create a homogeneous suspension. The sample was incubated for 30 min at 4 °C with constant mixing. Following incubation, the tubes were centrifuged again at 16,000×*g* for 15 min at 4 °C. The supernatant containing solubilized membrane and membrane-associated proteins was collected in a new tube. The prepared samples were stored at -80 °C or used directly for downstream analysis.

### 4.17 *In vivo* biotinylation assay

*In vivo* biotinylation was performed as reported previously with minor modifications [94]. Mice were anesthetized via intraperitoneal injection of ketamine (7.5 mg/kg), xylazine (20 mg/kg), and acepromazine (3 mg/kg). Following anesthesia, transcardial perfusion was performed with 10 mL of pre-warmed PBS containing 10% dextrane 40 for 10 min at a flow rate of 1 mL/min, and subsequently, with 15 mL of pre-warmed biotinylation buffer containing non-permeable, cleavable 1 mg/mL EZ Link Sulfo–NHS–SS-Biotin (Thermofisher, #21331) in PBS containing 10% dextrane 40 for 10 min at a flow rate of 1.5 mL/min. To inactivate the EZ Link Sulfo–NHS–SS-Biotin, mice were perfused with pre-warmed 15 mL of inactivation buffer, containing 50 mM Tris/HCl and 10% dextrane 40 in PBS, at a flow rate of 1.5 mL/min. In parallel, the liver surface was sprinkled with inactivation sprinkling buffer containing 50 mM Tris/HCl in PBS and then harvested and snap-frozen. For the pulldown of biotinylated surface proteins, livers were homogenized using 15 strokes of a Dounce homogenizer in IP lysis buffer (25 mM Tris/HCl [pH 7.4], 150 mM NaCl, 1 mM EDTA, 1% NP-40, 5% glycerol) supplemented with phosphatase and protease inhibitors, followed by sonification for 20 s. Equal amount of protein for each sample was incubated with pre-washed NeutrAvidin Agarose Resins (Thermo Fisher, #29200). Afterward, NeutrAvidin Agarose Resins were washed three times and biotinylated proteins were eluted with 2*x* SDS sample buffer containing 0.25 M DTT, boiled for 5 min at 95 °C and analyzed using immunoblotting as described below.

### 4.18 Gene expression analysis

A piece of snap-frozen liver tissue was homogenized in QIAzol Lysis Reagent (Qiagen). RNA was isolated by chloroform extraction, precipitated with isopropanol, washed with ethanol, and dissolved in RNase/DNase-free water. 2 μg of RNA were used as input for cDNA synthesis, and 20 ng of cDNA was used for quantitative reverse transcription PCR with the FastStart SYBR Green Master (Roche) and the QuantStudio 7 Flex Real-Time PCR System (Applied Biosystems). The qRT-PCR program was as follows: 50 °C/2 min; 95 °C/10 min; 40 cycles with 95 °C/15 s; and 60 °C/1 min. Gene expression was calculated using the ΔΔCT method using QuantStudio Real-Time PCR Software (Applied Biosystems), with *Ppia* expression as an internal control. Primer sets used for gene expression analysis are listed in Supplemental Table 3.

For RNA sequencing analysis, 6 representative liver samples were selected, and total RNA was isolated with the RNAeasy plus kit (#74134, Qiagen) according to the manufacturer's instructions. Quantity and quality were determined using nanodrop and gel electrophoresis, respectively. Library preparation, sequencing, and analysis were done by Novogene Co. Ltd. Europe. A heatmap was constructed using the z-score calculated from the FPKM values, to visualize the expression of genes involved in cholesterol biosynthesis.

### 4.19 Immunoblotting

Liver and cell samples were homogenized in NP-40 buffer (0.1% Nonidet P-40, 0.4 M NaCl, 10 mM Tris-HCl [pH 8.0], and 1 mM EDTA) supplemented with 1 mM DTT and protease and phosphatase inhibitors (Roche). Protein concentrations were measured with the Bradford assay (Bio-Rad), and samples were diluted in 4*x* sample buffer. Samples were boiled for 5 min at 95 °C prior to loading on gel, except for samples used to visualize NPC1 and SCARB2. Proteins were separated with SDS-PAGE and subsequently transferred to Amersham Hybond-P PVDF Transfer Membrane (RPN303F, GE Healthcare). Membranes were blocked in 5% milk in TBS buffer containing 0.01% Tween 20 and incubated with the antibodies listed in Supplemental Table 3. Proteins of interest were visualized and quantified with the ChemiDoc XRS + System and Image Lab software version 5.2.1 (Bio-Rad).

### 4.20 Discovery-based (untargeted) proteomic analysis

Discovery-based proteomics was used to determine relative protein concentrations in whole liver samples and sucrose-fractionated liver samples. For whole liver samples, six representative samples were selected from each group of mice. In-gel digestion was performed on 50 μg of liver lysates or 30 μL from the collected sucrose fractions as described previously [9].

For the whole liver lysates, the discovery-based proteomics analyses (Label Free Quantification) were performed as described previously [95]. A rerun of the whole liver lysates was done with a digestion based on the published SP3 digestion protocol [96] using the discovery-based proteomics settings similar to the sucrose gradient samples, to detect SOAT2. For the SP3 digestion protocol, 10 μg of total protein was diluted in 100 mM ammonium bicarbonate to a final volume of 25 μL as starting material. Reduction was performed by adding 10 μL 10 mM dithiothreitol and incubation for 30 min at 57 °C. Alkylation was done with 10 μL of 30 mM iodoacetamide for 30 min at RT under aluminum foil. The proteins were bound to 10 μL prewashed beads (1:1 mix of hydrophilic and hydrophobic Cytiva SeraMag beads, Fisher Scientific), and the samples were diluted to 50% acetonitrile with 55 μL acetonitrile. After removal of the acetonitrile, the beads were washed twice with 200 μL 80% v/v ethanol, once with 180 μL acetonitrile, and resuspended in 40 μL 2.5 ng/μL trypsin (V5111 sequencing grade modified trypsin, Promega) for overnight digestion at 37 °C. The digestion was stopped by addition of 10 μL 1% v/v formic acid and an equivalent of 1 μg was loaded on the Evosep tips for the untargeted proteomics analyses.

For the sucrose gradient samples, the discovery mass spectrometric analyses were performed on a quadrupole orbitrap mass spectrometer equipped with a nano-electrospray ion source (Orbitrap Exploris 480, Thermo Scientific). Chromatographic separation of the peptides was performed by liquid chromatography (LC) on an Evosep system (Evosep One, Evosep) using a nano-LC column (EV1137 Performance column 15 cm × 150 μm, 1.5 μm, Evosep; buffer A: 0.1% v/v formic acid, dissolved in water, buffer B: 0.1% v/v formic acid, dissolved in acetonitrile). Ten percent of the sucrose fraction digests were injected and separated using the 30SPD workflow (Evosep). The mass spectrometer was operated in positive ion mode and data-independent acquisition mode (DIA) using isolation windows of 16 *m*/*z* with a precursor mass range of 400–1000, switching the FAIMS between CV-45V and −60V with three scheduled MS1 scans during each screening of the precursor mass range.

Raw LC-MS data were processed with Spectronaut (16.0.220606) (Biognosys) with the standard settings of the directDIA workflow except that quantification was performed on MS1 with a mouse SwissProt database (www.uniprot.org, 17021 entries). For data quantification of total livers, local normalization was applied, and the Q-value filtering was set to the classic setting without applying imputation. With these data, z-scores were calculated and used to construct a heatmap to visualize the relative expression of lysosomal proteins. Alternatively, data were used to express relative SOAT2 levels. For sucrose fractionated samples, the Q-value filtering was set to the classic setting without applying normalization or imputation, and the data were used to visualize the expression of proteins in each sucrose fraction, relative to the total protein expression in all fractions. For LysoIP data, quantification intensities were exported without normalization or imputation and processed in R (x4.x).

### 4.21 LAL activity assay

LAL activity was determined in control and VPS35-deficient Hepa1-6 cells using the LysoLive™ Lysosomal Acid Lipase Assay Kit (ab253380, Abcam) according to the manufacturer's instructions for adherent cells. Cells were incubated with LysoLive™ LipaGreen™ for 6 h in serum-free medium prior to reading cells with flow cytometry. Cells were analyzed with the BD FACSVerse™ (BD, Biosciences). Data was processed with Kaluza software (Beckman Coulter).

### 4.22 Immunofluorescence and cholesterol staining

To detect free cholesterol and lysosomes, mouse primary hepatocytes were fixed with 4% PFA for 30 min and stored in 2% PFA at 4 °C before they were washed with PBS, quenched in 50 mM NH_4_Cl for 10 min, washed with PBS, permeabilized with filipin (0.5 mg/mL, Sigma–Aldrich #F9765) in PBS/1% bovine serum albumin (BSA, Sigma–Aldrich #A3803) for 30 min at 37 °C, and washed with PBS. Subsequently, cells were incubated with primary LAMP2A antibody (Abcam #18528, 1:200) in 1% BSA for 60 min at room temperature, washed with PBS, incubated with secondary antibody anti-rabbit Alexa 647 (molecular probes #A31573, 1:200) in 1 % BSA for 60 min at room temperature, and washed with PBS.

To detect CE, mouse primary hepatocytes were washed with PBS after quenching and incubated twice in 70% ethanol for 5 min to extract free cholesterol and incubated with cholesterol esterase (1.65 U/mL, Sigma–Aldrich #C3766 [97]) in PBS for 3h at 37 °C. Subsequently, cells were treated with filipin, washed with PBS, and mounted as described below or stained with perilipin-3 antibody (Proteintech, #10694-1-AP, 1:200) in 1% BSA for 60 min at room temperature, followed by PBS washes, incubation with secondary antibody anti-rabbit Alexa 647 (Molecular Probes, #A31573, 1:200) in 1% BSA for 60 min at room temperature, and PBS washes. After staining, cells were mounted in Mowiol-DABCO on microscopy slides.

Cells were imaged with a Zeiss LSM 880 confocal microscope and Airyscan processed, as described before [98]. The amount of FC in lysosomes was assessed by measuring filipin intensity in LAMP2-positive structures detected using Ilastik [99] and CellProfiler [100] with a custom module. Individual cells were detected on filipin channel by using CellPose [101]and manual curation in CellProfiler. Total intensity of filipin in LAMP2-positive structures per cell was measured and normalized to cell area (in pixels) to get the estimate for FC/cell area.

To assess CE levels in cells, bright filipin-positive structures in cholesterol esterase- and filipin-treated cells were detected by using Ilastik and CellProfiler. Individual cells were segmented on filipin channel using CellPose and manual curation in CellProfiler. Total filipin intensity in these structures per cell was measured and normalized to cell area (in pixels) to get the estimate for CE/cell area. Filipin intensity was set similarly for each exemplary image comparing WT and VPS35-deficient cells, except in Fig. S7B, where channel intensities were set to best demonstrate CE signal in perilipin-3-positive organelles. Significance was determined using an unpaired Student's t-test.

### 4.23 Ratiometric pH imaging of late endosomes and lysosomes using ApHID-dextran probe

Lysosomal pH was measured in live primary hepatocytes by ratiometric imaging using custom-made 70 kDa dextran polymers, conjugated to the pH-sensor ApHID and the pH-independent fluorophore Alexa 647, as described previously [39]. Hepatocytes were seeded in collagen-coated 35 mm imaging dishes with 4 quadrants and allowed to adhere for at least 4 h. For live cell imaging, cells were incubated overnight with 0.5 mg/mL dextran in complete medium, followed by a ≥3-hour chase in fresh medium to ensure dextran localization to late endosomes and lysosomes. For calibration, a separate set of cells was pulsed with 2 mg/mL dextran for 2 h, chased for 3 h, washed, fixed in 0.5% PFA for 5 min, and equilibrated in pH 5.0 TRIS maleate buffer containing 40 mM methylamine, 40 mM sodium acetate, and 40 μM monensin for 30 min at 37 °C prior to imaging. ApHID/Alexa 647 ratiometric imaging was performed on a Stellaris confocal microscope (Leica Systems) using a 20× water objective with sequential acquisition of the ApHID and Alexa 647 channels. The pH-independent Alexa 647 channel was used to generate a binary mask of dextran-positive compartments, and integrated fluorescence intensities of both channels were measured within this mask. The ApHID/Alexa 647 ratio was calculated per quadrant (4–6 fields per quadrant, n = 6 quadrants) and converted to pH values by interpolation from a sigmoidal calibration curve fitted in GraphPad Prism, generated using the pH 5.0 fixed-cell measurements and ApHID's dynamic range previously determined in solution at 37 °C.

### 4.24 Click chemistry-based pulse chase assay

Primary hepatocytes were isolated from control and hepatic VPS35-deficient mice. After an overnight culture, cells were used for a pulse experiment. The pulse procedure was adapted from Morey et al. [50]. To deplete endogenous methionine, cells were washed twice with methionine-free DMEM (Thermo Fisher Scientific, #21013024) and incubated for 30 min at 37 °C prior to labeling. Cells were pulsed with 50 μM Click-iT l-azidohomoalanine (AHA; Thermo Fisher Scientific, #C10102) in methionine-free DMEM supplemented with 10% dialyzed fetal calf serum (FCS; Thermo Fisher Scientific, #A3382001) for 2, 4, 8, 12, 16, 24, 48 and 72 h at 37 °C. Medium containing fresh Click-iT AHA was replaced every 4 h to maintain consistent labeling conditions during extended pulse periods. For control experiments, cells were cultured in methionine-containing DMEM without AHA labeling. Following pulse, cells were collected on ice and lysed in ice-cold RIPA buffer (50 mM Tris-HCl pH 8.9, 150 mM NaCl, 1% Triton X-100, 0.5% sodium deoxycholate, 0.5% SDS) supplemented with protease and phosphatase inhibitors. Homogenates were sonicated (10 s at 40% amplitude) and centrifuged at 12,000 g for 10 min at 4 °C. Protein concentrations of cleared cell lysates were determined using BCA protein assay (Thermo Fisher Scientific). Equal protein amounts (normalized by BCA assay) were reacted with 10 μM Biotin-DIBO (Thermo Fisher Scientific, #C20023) in RIPA buffer containing protease inhibitors for 1 h at 21 °C with gentle agitation. Following click chemistry, biotin-labeled lysates were incubated with 1 μg of anti-SCARB2 antibody (Cell Signaling Technology, #27960S) for 1.5 h at 4 °C with continuous rotation. Protein A/G PLUS-Agarose beads (pre-washed in RIPA buffer; 50 μL per reaction) were added and incubated for 2 h at 4 °C with rotation. Immunocaptured samples were washed twice with ice-cold 0.5% SDS in RIPA buffer. Biotin-labeled whole-cell lysates (input controls) and immunoprecipitated samples were denatured in 2 × Laemmli sample buffer containing 5% 2-mercaptoethanol, resolved on SDS-PAGE gels, and transferred to PVDF membranes. Biotin-labeled proteins were detected using HRP-conjugated streptavidin (30 min at room temperature) using standard ECL substrate.

### 4.25 Statistical analysis

Unless indicated otherwise, data are presented as mean ± SEM. Statistical analyses were performed with GraphPad version 9.1 (GraphPad software). Differences between two groups were determined with unpaired two-tailed Student's *t* test. For comparing two variables between groups, two-way ANOVA with Tukey's multiple comparison test was performed. In all cases, P values of <0.05 were considered statistically significant. LysoIP proteomics data were analyzed in R (v4.x). For each protein, the log2 fold change between Vps35-KO and EV LysoIP samples (n = 5 per group) was calculated as the difference in group mean log2 intensity, and significance was tested by unpaired two-sample Welch t-test. p-values were adjusted for multiple testing across all quantified proteins using Benjamini–Hochberg; proteins with FDR <0.05 and log2FC > 0.585 or < were considered differentially abundant.

### 4.26 Ethics declaration

This study was conducted in accordance with the following guidelines for animal welfare and/or reporting: The Central Authority for Scientific Procedures on Animals (CCD). Ethics committee approval was not required. No human studies were involved.

## CRediT authorship contribution statement

**Cristy R.C. Verzijl:** Writing – original draft, Visualization, Methodology, Investigation, Formal analysis, Data curation, Conceptualization, Funding acquisition, Writing – review & editing. **Markus G. Barbosa:** Writing – original draft, Methodology, Investigation, Formal analysis, Conceptualization, Writing – review & editing. **Dyonne Y. Vos:** Writing – original draft, Visualization, Methodology, Investigation, Formal analysis, Data curation, Conceptualization, Writing – review & editing. **Andries H. Heida:** Writing – review & editing, Investigation, Formal analysis, Data curation. **Amandine Caillaud:** Methodology, Investigation, Formal analysis, Data curation. **Lauri Vanharanta:** Visualization, Methodology, Formal analysis, Data curation. **Xiaoke Ge:** Formal analysis, Data curation. **Joël J. Tissink:** Methodology, Formal analysis. **Mirjam H. Koster:** Methodology, Formal analysis. **Marieke Smit:** Methodology, Formal analysis. **Nicolette Huijkman:** Methodology, Formal analysis. **Niels J. Kloosterhuis:** Methodology, Formal analysis. **Aurore Girardeau:** Methodology, Formal analysis. **Zoé Begué-Racapé:** Methodology, Formal analysis. **Vincent W. Bloks:** Data curation. **Rick Havinga:** Methodology. **Marceline M. Fuh:** Formal analysis. **Fulvio Reggiori:** Writing – review & editing, Methodology, Formal analysis. **Muriel Mari:** Methodology, Formal analysis. **Ludger Scheja:** Methodology, Formal analysis. **Folkert Kuipers:** Methodology, Formal analysis. **Santiago Solé-Domènech:** Resources, Methodology, Formal analysis. **Sander Kooijman:** Writing – review & editing, Resources, Methodology, Formal analysis. **Joerg Heeren:** Resources, Methodology, Formal analysis. **Jan Freark de Boer:** Methodology, Formal analysis, Data curation. **Patrick C.N. Rensen:** Writing – review & editing, Resources, Methodology, Formal analysis. **Elina Ikonen:** Writing – review & editing, Visualization, Methodology, Formal analysis. **Antoine Rimbert:** Writing – review & editing, Methodology, Formal analysis. **Justina C. Wolters:** Writing – review & editing, Methodology, Formal analysis, Data curation. **Jan Albert Kuivenhoven:** Writing – review & editing, Supervision, Funding acquisition. **Bart van de Sluis:** Writing – original draft, Visualization, Supervision, Resources, Project administration, Methodology, Investigation, Funding acquisition, Formal analysis, Conceptualization.

## Declaration of competing interest

The authors declare no competing interests.

## Data Availability

Data will be made available on request.

## References

[1] Vos D.Y., van de Sluis B. (2021). Function of the endolysosomal network in cholesterol homeostasis and metabolic-associated fatty liver disease (MAFLD). Mol Metabol.

[2] Brown M.S., Goldstein J.L. (1976). Receptor-mediated control of cholesterol metabolism. Science.

[3] Ikonen E. (2008). Cellular cholesterol trafficking and compartmentalization. Nat Rev Mol Cell Biol.

[4] Goldstein J.L., Dana S.E., Faust J.R., Beaudet A.L., Brown M.S. (1975). Role of lysosomal acid lipase in the metabolism of plasma low density lipoprotein. Observations in cultured fibroblasts from a patient with cholesteryl ester storage disease. J Biol Chem.

[5] Infante R.E., Wang M.L., Radhakrishnan A., Kwon H.J., Brown M.S., Goldstein J.L. (2008). NPC2 facilitates bidirectional transfer of cholesterol between NPC1 and lipid bilayers, a step in cholesterol egress from lysosomes. Proc Natl Acad Sci.

[6] Heybrock S., Kanerva K., Meng Y., Ing C., Liang A., Xiong Z.-J. (2019). Lysosomal integral membrane protein-2 (LIMP-2/SCARB2) is involved in lysosomal cholesterol export. Nat Commun.

[7] Lange Y. (1991). Disposition of intracellular cholesterol in human fibroblasts. JLR (J Lipid Res).

[8] Brown M.S., Goldstein J.L. (1997). The SREBP pathway: regulation of cholesterol metabolism by proteolysis of a membrane-bound transcription factor. Cell.

[9] Fedoseienko A., Wijers M., Wolters J.C., Dekker D., Smit M., Huijkman N. (2018). The COMMD family regulates plasma LDL levels and attenuates atherosclerosis through stabilizing the CCC complex in endosomal LDLR trafficking. Circ Res.

[10] Bartuzi P., Billadeau D.D., Favier R., Rong S., Dekker D., Fedoseienko A. (2016). CCC- and WASH-mediated endosomal sorting of LDLR is required for normal clearance of circulating LDL. Nat Commun.

[11] Wijers M., Zanoni P., Liv N., Vos D.Y., Jäckstein M.Y., Smit M. (2019). The hepatic WASH complex is required for efficient plasma LDL and HDL cholesterol clearance. JCI Insight.

[12] Vanier M.T. (2010). Niemann-Pick disease type C. Orphanet J Rare Dis.

[13] Reiner Ž., Guardamagna O., Nair D., Soran H., Hovingh K., Bertolini S. (2014). Lysosomal acid lipase deficiency – an under-recognized cause of dyslipidaemia and liver dysfunction. Atherosclerosis.

[14] Pericleous M., Kelly C., Wang T., Livingstone C., Ala A. (2017). Wolman's disease and cholesteryl ester storage disorder: the phenotypic spectrum of lysosomal acid lipase deficiency. Lancet Gastroenterol Hepatol.

[15] Kelly D.A., Portmann B., Mowat A.P., Sherlock S., Lake B.D. (1993). Niemann-Pick disease type C: diagnosis and outcome in children, with particular reference to liver disease. J Pediatr.

[16] Haft C.R., Sierra M. de la Luz, Bafford R., Lesniak M.A., Barr V.A., Taylor S.I. (2000). Human orthologs of yeast vacuolar protein sorting proteins Vps26, 29, and 35: assembly into multimeric complexes. Mol Biol Cell.

[17] Burd C., Cullen P.J. (2014). Retromer: a master conductor of endosome sorting. Cold Spring Harbor Perspect Biol.

[18] Wang J., Fedoseienko A., Chen B., Burstein E., Jia D., Billadeau D.D. (2018). Endosomal receptor trafficking: retromer and beyond. Traffic.

[19] Seaman M.N.J. (2021). The retromer complex: from genesis to revelations. Trends Biochem Sci.

[20] Marquer C., Tian H., Yi J., Bastien J., Dall'Armi C., Yang-Klingler Y. (2016). Arf6 controls retromer traffic and intracellular cholesterol distribution via a phosphoinositide-based mechanism. Nat Commun.

[21] Arighi C.N., Hartnell L.M., Aguilar R.C., Haft C.R., Bonifacino J.S. (2004). Role of the mammalian retromer in sorting of the cation-independent mannose 6-phosphate receptor. J Cell Biol.

[22] Seaman M.N.J. (2004). Cargo-selective endosomal sorting for retrieval to the Golgi requires retromer. J Cell Biol.

[23] Dominko K., Rastija A., Sobocanec S., Vidatic L., Meglaj S., Babic A.L. (2021). Impaired retromer function in niemann-pick type C disease is dependent on intracellular cholesterol accumulation. Int J Mol Sci.

[24] Dostál V., Humhalová T., Beránková P., Pácalt O., Libusová L. (2023). SWIP mediates retromer-independent membrane recruitment of the WASH complex. Traffic.

[25] Seaman M.N.J., Gautreau A., Billadeau D.D. (2013). Retromer-mediated endosomal protein sorting: all WASHed up. Trends Cell Biol.

[26] Cui Y., Carosi J.M., Yang Z., Ariotti N., Kerr M.C., Parton R.G. (2019). Retromer has a selective function in cargo sorting via endosome transport carriers. JCB (J Cell Biol).

[27] Carosi J.M., Hein L.K., Hurk M. van den, Adams R., Milky B., Singh S. (2021). Retromer regulates the lysosomal clearance of MAPT/tau. Autophagy.

[28] McNally K.E., Faulkner R., Steinberg F., Gallon M., Ghai R., Pim D. (2017). Retriever is a multiprotein complex for retromer-independent endosomal cargo recycling. Nat Cell Biol.

[29] Vos D.Y., Wijers M., Smit M., Huijkman N., Kloosterhuis N.J., Wolters J.C. (2022). Cargo-specific role for retriever subunit VPS26C in hepatocyte lipoprotein receptor recycling to control postprandial triglyceride-rich lipoproteins. Arterioscler Thromb Vasc Biol.

[30] Laqtom N.N., Dong W., Medoh U.N., Cangelosi A.L., Dharamdasani V., Chan S.H. (2022). CLN3 is required for the clearance of glycerophosphodiesters from lysosomes. Nature.

[31] Rohlmann A., Gotthardt M., Hammer R.E., Herz J. (1998). Inducible inactivation of hepatic LRP gene by cre-mediated recombination confirms role of LRP in clearance of chylomicron remnants. J Clin Investig.

[32] Ronda O.A.H.O., Dijk T.H., Verkade H.J., Groen A.K. (2016). Measurement of intestinal and peripheral cholesterol fluxes by a dual-tracer balance method. Curr Protoc Mol Biol.

[33] Neese R.A., Faix D., Kletke C., Wu K., Wang A.C., Shackleton C.H. (1993). Measurement of endogenous synthesis of plasma cholesterol in rats and humans using MIDA. Am J Physiol Endocrinol Metabol.

[34] Veen J.N. van der, Dijk T.H. van, Vrins C.L.J., Meer H. van, Havinga R., Bijsterveld K. (2009). Activation of the liver X receptor stimulates Trans-intestinal excretion of plasma cholesterol. J Biol Chem.

[35] Aqul A., Lopez A.M., Posey K.S., Taylor A.M., Repa J.J., Burns D.K. (2014). Hepatic entrapment of esterified cholesterol drives continual expansion of whole body sterol pool in lysosomal acid lipase-deficient mice. Am J Physiol Gastrointest Liver Physiol.

[36] Zavodszky E., Seaman M.N.J., Moreau K., Jimenez-Sanchez M., Breusegem S.Y., Harbour M.E. (2014). Mutation in VPS35 associated with Parkinson's disease impairs WASH complex association and inhibits autophagy. Nat Commun.

[37] Maruzs T., Lőrincz P., Szatmári Z., Széplaki S., Sándor Z., Lakatos Z. (2015). Retromer ensures the degradation of autophagic cargo by maintaining lysosome function in drosophila. Traffic.

[38] Daly J.L., Danson C.M., Lewis P.A., Zhao L., Riccardo S., Filippo L.D. (2023). Multi-omic approach characterises the neuroprotective role of retromer in regulating lysosomal health. Nat Commun.

[39] Solé-Domènech S., Singh P.K., Funes L., Ma C.-I.J., Warren J.D., Maxfield F.R. (2025). Real-time pH imaging of macrophage lysosomes using the pH-sensitive probe ApHID. Cell Rep Methods.

[40] Gelsthorpe M.E., Baumann N., Millard E., Gale S.E., Langmade S.J., Schaffer J.E. (2008). Niemann-Pick type C1 I1061T mutant encodes a functional protein that is selected for endoplasmic Reticulum-associated degradation due to protein misfolding. J Biol Chem.

[41] Schultz M.L., Krus K.L., Kaushik S., Dang D., Chopra R., Qi L. (2018). Coordinate regulation of mutant NPC1 degradation by selective ER autophagy and MARCH6-dependent ERAD. Nat Commun.

[42] Vonk W.I.M., Bie P. de, Wichers C.G.K., Berghe P.V.E. van den, Plaats R. van der, Berger R. (2011). The copper-transporting capacity of ATP7A mutants associated with Menkes disease is ameliorated by COMMD1 as a result of improved protein expression. Cell Mol Life Sci : CMLS.

[43] Schultz M.L., Krus K.L., Lieberman A.P. (2016). Lysosome and endoplasmic reticulum quality control pathways in Niemann-Pick type C disease. Brain Research 1649(Pt B).

[44] Buratta S., Tancini B., Sagini K., Delo F., Chiaradia E., Urbanelli L. (2020). Lysosomal exocytosis, exosome release and secretory autophagy: the Autophagic- and endo-lysosomal systems Go extracellular. Int J Mol Sci.

[45] Strauss K., Goebel C., Runz H., Möbius W., Weiss S., Feussner I. (2010). Exosome secretion ameliorates lysosomal storage of cholesterol in niemann-pick type C disease. J Biol Chem.

[46] Samie M.A., Xu H. (2014). Lysosomal exocytosis and lipid storage disorders. JLR (J Lipid Res).

[47] Peng X., Yang L., Ma Y., Li X., Yang S., Li Y. (2021). IKKβ activation promotes amphisome formation and extracellular vesicle secretion in tumor cells. Biochim Biophys Acta Mol Cell Res.

[48] Eitan E., Suire C., Zhang S., Mattson M.P. (2016). Impact of lysosome status on extracellular vesicle content and release. Ageing Res Rev.

[49] Kim J.H., Lee C.-H., Baek M.-C. (2022). Dissecting exosome inhibitors: therapeutic insights into small-molecule chemicals against cancer. Exp Mol Med.

[50] Morey T.M., Esmaeili M.A., Duennwald M.L., Rylett R.J. (2021). SPAAC Pulse-Chase: a novel click chemistry-based method to determine the half-life of cellular proteins. Front Cell Dev Biol.

[51] Zimprich A., Benet-Pagès A., Struhal W., Graf E., Eck S.H., Offman M.N. (2011). A mutation in VPS35, encoding a subunit of the retromer complex, causes late-onset parkinson disease. Am J Hum Genet.

[52] Vilariño-Güell C., Wider C., Ross O.A., Dachsel J.C., Kachergus J.M., Lincoln S.J. (2011). VPS35 mutations in parkinson disease. Am J Hum Genet.

[53] Small S.A., Kent K., Pierce A., Leung C., Kang M.S., Okada H. (2005). Model-guided microarray implicates the retromer complex in Alzheimer's disease. - PubMed - NCBI. Ann Neurol.

[54] Phillips-Krawczak C.A., Singla A., Starokadomskyy P., Deng Z., Osborne D.G., Li H. (2015). COMMD1 is linked to the WASH complex and regulates endosomal trafficking of the copper transporter ATP7A. Mol Biol Cell.

[55] McNally K.E., Cullen P.J. (2018). Endosomal retrieval of cargo: Retromer is not alone. Trends Cell Biol.

[56] Morales S.V., Mahmood A., Pollard J., Mayne J., Figeys D., Wiseman P.W. (2023). The LDL receptor is regulated by membrane cholesterol as revealed by fluorescence fluctuation analysis. Biophys J.

[57] Islam M.M., Hlushchenko I., Pfisterer S.G. (2022). Low-density lipoprotein internalization, degradation and receptor recycling along membrane contact sites. Front Cell Dev Biol.

[58] Fischer A.W., Albers K., Krott L.M., Hoffzimmer B., Heine M., Schmale H. (2018). The adaptor protein PID1 regulates receptor-dependent endocytosis of postprandial triglyceride-rich lipoproteins. Mol Metabol.

[59] Gordts P.L.S.M., Bartelt A., Nilsson S.K., Annaert W., Christoffersen C., Nielsen L.B. (2012). Impaired LDL receptor-related protein 1 translocation correlates with improved dyslipidemia and atherosclerosis in apoE-Deficient mice. PLoS One.

[60] Frolov A., Zielinski S.E., Crowley J.R., Dudley-Rucker N., Schaffer J.E., Ory D.S. (2003). NPC1 and NPC2 regulate cellular cholesterol homeostasis through generation of low density lipoprotein cholesterol-derived oxysterols. J Biol Chem.

[61] Liscum L., Faust J.R. (1987). Low density lipoprotein (LDL)-mediated suppression of cholesterol synthesis and LDL uptake is defective in Niemann-Pick type C fibroblasts. J Biol Chem.

[62] Liu B., Turley S.D., Burns D.K., Miller A.M., Repa J.J., Dietschy J.M. (2009). Reversal of defective lysosomal transport in NPC disease ameliorates liver dysfunction and neurodegeneration in the npc1−/− mouse. Proc Natl Acad Sci.

[63] Parini P., Davis M., Lada A.T., Erickson S.K., Wright T.L., Gustafsson U. (2004). ACAT2 is localized to hepatocytes and is the major cholesterol-esterifying enzyme in human liver. Circulation.

[64] Zeng J., Acin-Perez R., Assali E.A., Martin A., Brownstein A.J., Petcherski A. (2023). Restoration of lysosomal acidification rescues autophagy and metabolic dysfunction in non-alcoholic fatty liver disease. Nat Commun.

[65] Cao X., Lenk G.M., Mikusevic V., Mindell J.A., Meisler M.H. (2023). The chloride antiporter CLCN7 is a modifier of lysosome dysfunction in FIG 4 and VAC14 mutants. PLoS Genet.

[66] Nicoli E.-R., Weston M.R., Hackbarth M., Becerril A., Larson A., Zein W.M. (2019). Lysosomal Storage and Albinism Due to Effects of a De Novo CLCN7 Variant on Lysosomal Acidification. Am J Hum Genet.

[67] Ko D.C., Gordon M.D., Jin J.Y., Scott M.P. (2001). Dynamic movements of organelles containing Niemann-Pick C1 protein: NPC1 involvement in late endocytic events. Mol Biol Cell.

[68] Markmann S., Krambeck S., Hughes C.J., Mirzaian M., Aerts J.M.F.G., Saftig P. (2017). Quantitative proteome analysis of mouse liver lysosomes provides evidence for mannose 6-phosphate-independent targeting mechanisms of acid hydrolases in mucolipidosis II. Mol Cell Proteomics.

[69] Ghosh P., Dahms N.M., Kornfeld S. (2003). Mannose 6-phosphate receptors: new twists in the tale. Nat Rev Mol Cell Biol.

[70] Follett J., Norwood S.J., Hamilton N.A., Mohan M., Kovtun O., Tay S. (2014). The Vps35 D620N mutation linked to Parkinson's disease disrupts the cargo sorting function of retromer. Traffic.

[71] Bugarcic A., Zhe Y., Kerr M.C., Griffin J., Collins B.M., Teasdale R.D. (2011). Vps26A and Vps26B subunits define distinct retromer complexes. Traffic.

[72] Rojas R., Vlijmen T. van., Mardones G.A., Prabhu Y., Rojas A.L., Mohammed S. (2008). Regulation of retromer recruitment to endosomes by sequential action of Rab5 and Rab7. J Cell Biol.

[73] Zhang J., Ali M.Y., Chong H.B., Tien P.-C., Woods J., Noble C. (2025). Oxidation of retromer complex controls mitochondrial translation. Nature.

[74] Boomen D.J.H. van den, Sienkiewicz A., Berlin I., Jongsma M.L.M., Elsland D.M. van, Luzio J.P. (2020). A trimeric Rab7 GEF controls NPC1-dependent lysosomal cholesterol export. Nat Commun.

[75] Nguyen M.K.L., Nikenich M.R., Seifert K., Pinkenburg C., Lai F., Walther H.-L. (2026). Small molecules to elevate Rab7-GTPase activity and lower cholesterol accumulation in Niemann-Pick type C disease. Pharm Res.

[76] Meneses-Salas E., García-Melero A., Kanerva K., Blanco-Muñoz P., Morales-Paytuvi F., Bonjoch J. (2020). Annexin A6 modulates TBC1D15/Rab7/StARD3 axis to control endosomal cholesterol export in NPC1 cells. Cell Mol Life Sci.

[77] Choudhury A., Dominguez M., Puri V., Sharma D.K., Narita K., Wheatley C.L. (2002). Rab proteins mediate Golgi transport of caveola-internalized glycosphingolipids and correct lipid trafficking in Niemann-Pick C cells. J Clin Investig.

[78] Wouters K., Gorp P.J. van., Bieghs V., Gijbels M.J., Duimel H., Lütjohann D. (2008). Dietary cholesterol, rather than liver steatosis, leads to hepatic inflammation in hyperlipidemic mouse models of nonalcoholic steatohepatitis. Hepatology.

[79] Jouffe C., Gobet C., Martin E., Métairon S., Morin-Rivron D., Masoodi M. (2016). Perturbed rhythmic activation of signaling pathways in mice deficient for sterol carrier protein 2-dependent diurnal lipid transport and metabolism. Sci Rep.

[80] Barbosa M.G., Vos D.Y., Verzijl C.R.C., Heida A.H., Bakker M., Kloosterhuis N.J. (2026). Vacuolar protein sorting 35 controls hepatocellular proliferation through SRC signaling and promotes diethyl nitrosamine–induced tumor initiation. Cell Mol Gastroenterol Hepatol.

[81] Groot R.E.A. de., Farin H.F., rkovÃ M.M., Es J.H. van., Clevers H.C., Korswagen H.C. (2013). Retromer dependent recycling of the Wnt secretion factor Wls is dispensable for stem cell maintenance in the mammalian intestinal epithelium. - PubMed - NCBI. PLoS One.

[82] Platt R.J., Chen S., Zhou Y., Yim M.J., Swiech L., Kempton H.R. (2014). CRISPR-Cas9 Knockin mice for genome editing and cancer modeling. Cell.

[83] Boer P. de., Pirozzi N.M., Wolters A.H.G., Kuipers J., Kusmartseva I., Atkinson M.A. (2020). Large-scale electron microscopy database for human type 1 diabetes. Nat Commun.

[84] Kuipers J., Giepmans B.N.G. (2020). Neodymium as an alternative contrast for uranium in electron microscopy. Histochem Cell Biol.

[85] Bligh E.G., Dyer W.J. (1959). A rapid method of total lipid extraction and purification. Can J Biochem Physiol.

[86] Evangelakos I., Schwinge D., Worthmann A., John C., Roeder N., Pertzborn P. (2021). Oxysterol 7-α hydroxylase (CYP7B1) attenuates metabolic-associated fatty liver disease in mice at thermoneutrality. Cells.

[87] Abu-Remaileh M., Wyant G.A., Kim C., Laqtom N.N., Abbasi M., Chan S.H. (2017). Lysosomal metabolomics reveals V-ATPase- and mTOR-dependent regulation of amino acid efflux from lysosomes. Science.

[88] Loregger A., Raaben M., Nieuwenhuis J., Tan J.M.E., Jae L.T., Hengel L.G. (2020). Haploid genetic screens identify SPRING/C12ORF49 as a determinant of SREBP signaling and cholesterol metabolism. Nat Commun.

[89] Graaf I.A.M. de, Olinga P., Jager M.H. de, Merema M.T., Kanter R. de, Kerkhof E.G. van de (2010). Preparation and incubation of precision-cut liver and intestinal slices for application in drug metabolism and toxicity studies. Nat Protoc.

[90] Roudaut M., Caillaud A., Souguir Z., Bray L., Girardeau A., Rimbert A. (2024). Human induced pluripotent stem cells-derived liver organoids grown on a Biomimesys® hyaluronic acid-based hydroscaffold as a new model for studying human lipoprotein metabolism. Bioeng Transl Med.

[91] Bray L., Caillaud A., Girardeau A., Patitucci M., May C.L., Cariou B. (2022). Generation of a GPR146 knockout human induced pluripotent stem cell line (ITXi001-A-1). Stem Cell Res.

[92] Caillaud A., Lévêque A., Thédrez A., Girardeau A., Canac R., Bray L. (2022). FACS-assisted CRISPR-Cas9 genome editing of human induced pluripotent stem cells. STAR Protoc.

[93] Harrison S.P., Siller R., Tanaka Y., Chollet M.E., Morena-Barrio M.E. de la, Xiang Y. (2023). Scalable production of tissue-like vascularized liver organoids from human PSCs. Exp Mol Med.

[94] Rybak J.-N., Ettorre A., Kaissling B., Giavazzi R., Neri D., Elia G. (2005). In vivo protein biotinylation for identification of organ-specific antigens accessible from the vasculature. Nat Methods.

[95] Lin C., Mostafa A., Jans A., Wolters J.C., Mohamed M.R., Vorst E.P.C.V. (2023). Targeting ligand independent tropism of siRNA-LNP by small molecules for directed therapy of liver or Myeloid immune cells. Adv Healthcare Mater.

[96] Müller T., Kalxdorf M., Longuespée R., Kazdal D.N., Stenzinger A., Krijgsveld J. (2020). Automated sample preparation with SP3 for low-input clinical proteomics. Mol Syst Biol.

[97] Curcio C.A., Millican C.L., Bailey T., Kruth H.S. (2001). Accumulation of cholesterol with age in human Bruch's membrane. Investig Ophthalmol Vis Sci.

[98] Dumesnil C., Vanharanta L., Prasanna X., Omrane M., Carpentier M., Bhapkar A. (2023). Cholesterol esters form supercooled lipid droplets whose nucleation is facilitated by triacylglycerols. Nat Commun.

[99] Morawski M., Kirilina E., Scherf N., Jäger C., Reimann K., Trampel R. (2018). Developing 3D microscopy with CLARITY on human brain tissue: towards a tool for informing and validating MRI-based histology. Neuroimage.

[100] Carpenter A.E., Jones T.R., Lamprecht M.R., Clarke C., Kang I.H., Friman O. (2006). CellProfiler: image analysis software for identifying and quantifying cell phenotypes. Genome Biol.

[101] Stringer C., Wang T., Michaelos M., Pachitariu M. (2021). Cellpose: a generalist algorithm for cellular segmentation. Nat Methods.

