# Supplementary material for: Hepatic retromer is essential for systemic cholesterol homeostasis by regulating lysosomal cholesterol handling
Source: Mol Metab. 2026 Oct 11;114:102456. doi: 10.1016/j.molmet.2026.102456 (PMC13717181; doi:10.1016/j.molmet.2026.102456)

Supplemental Figure 1

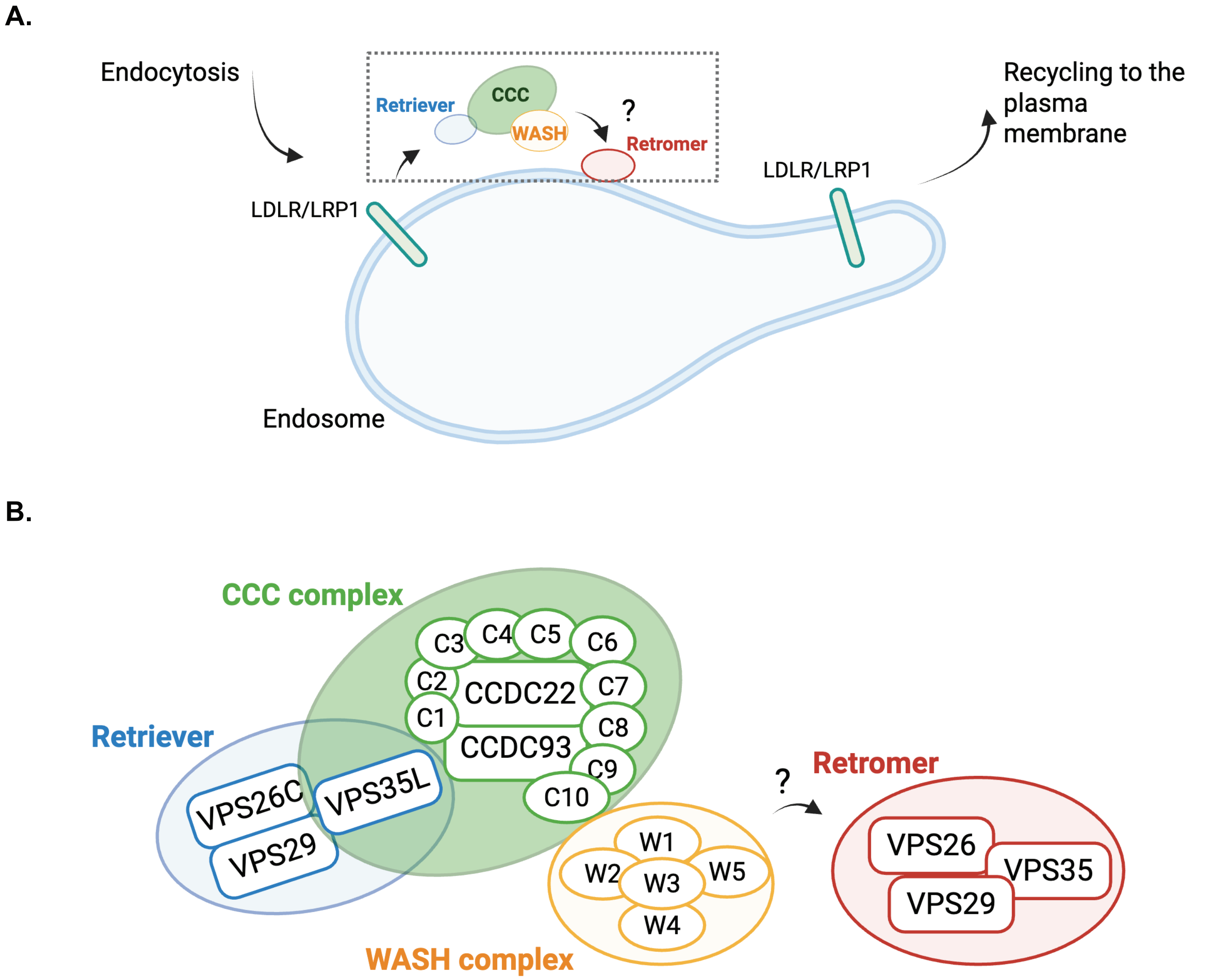

Supplemental Figure 2

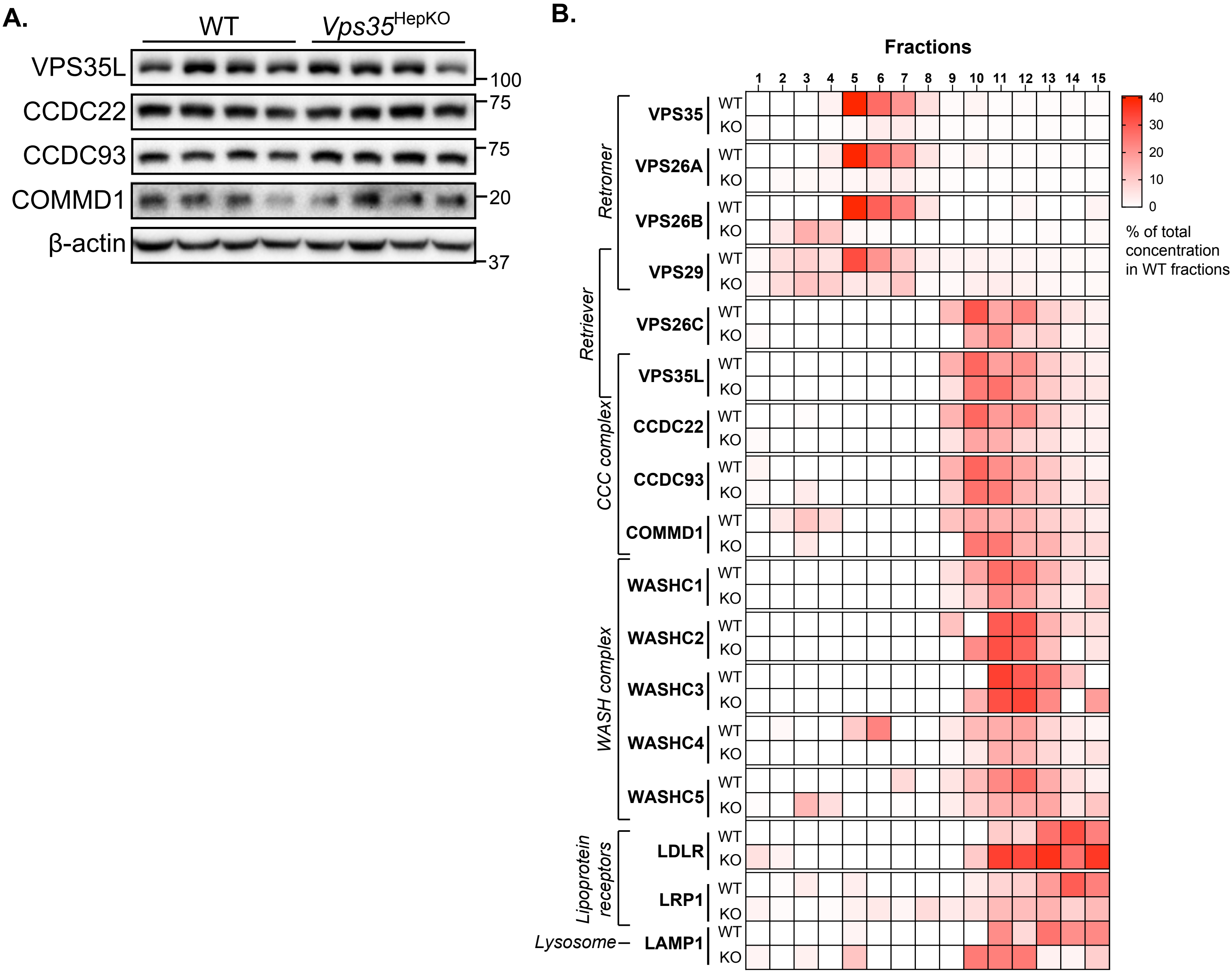

Supplemental Figure 3

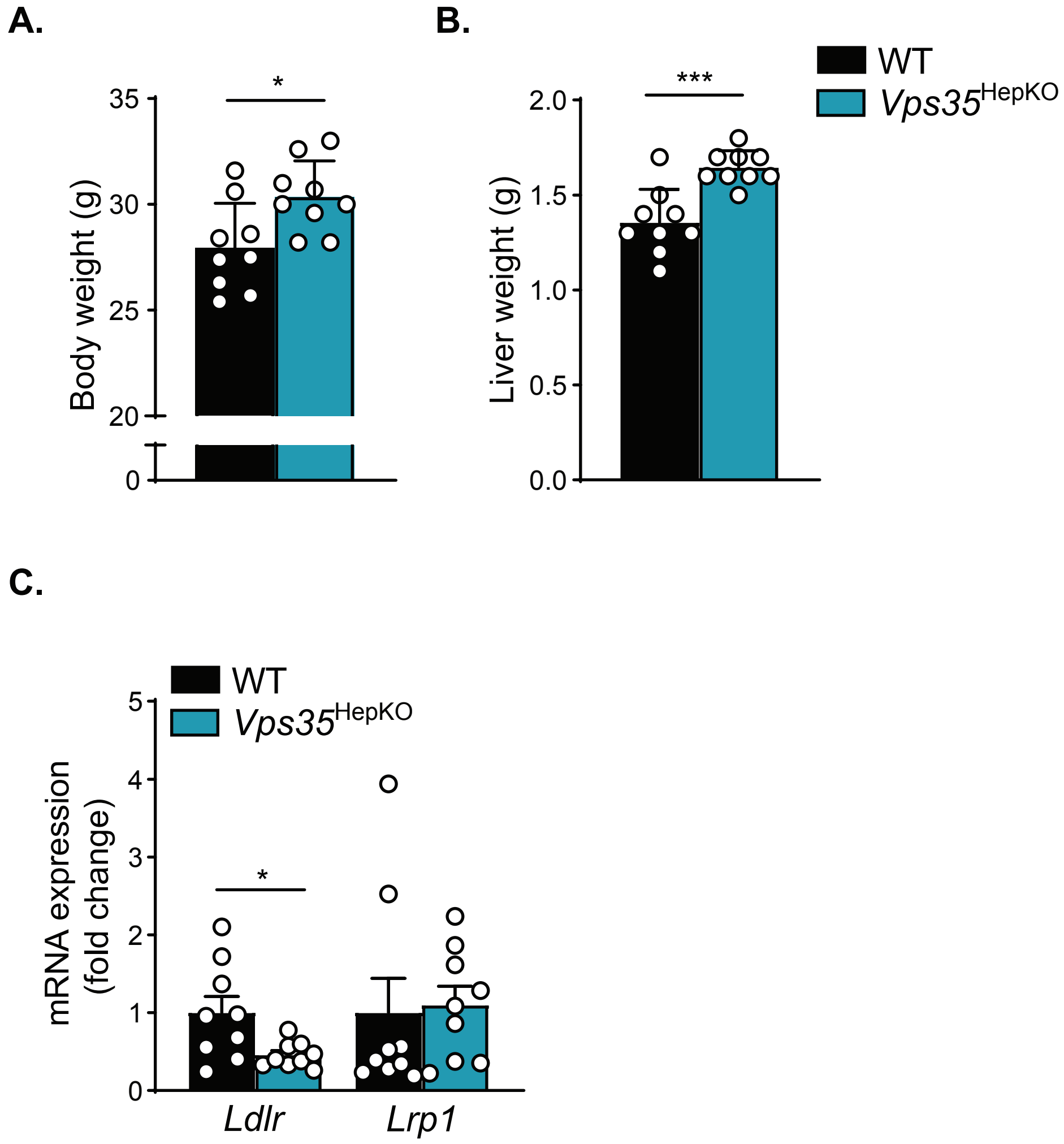

Supplemental Figure 4

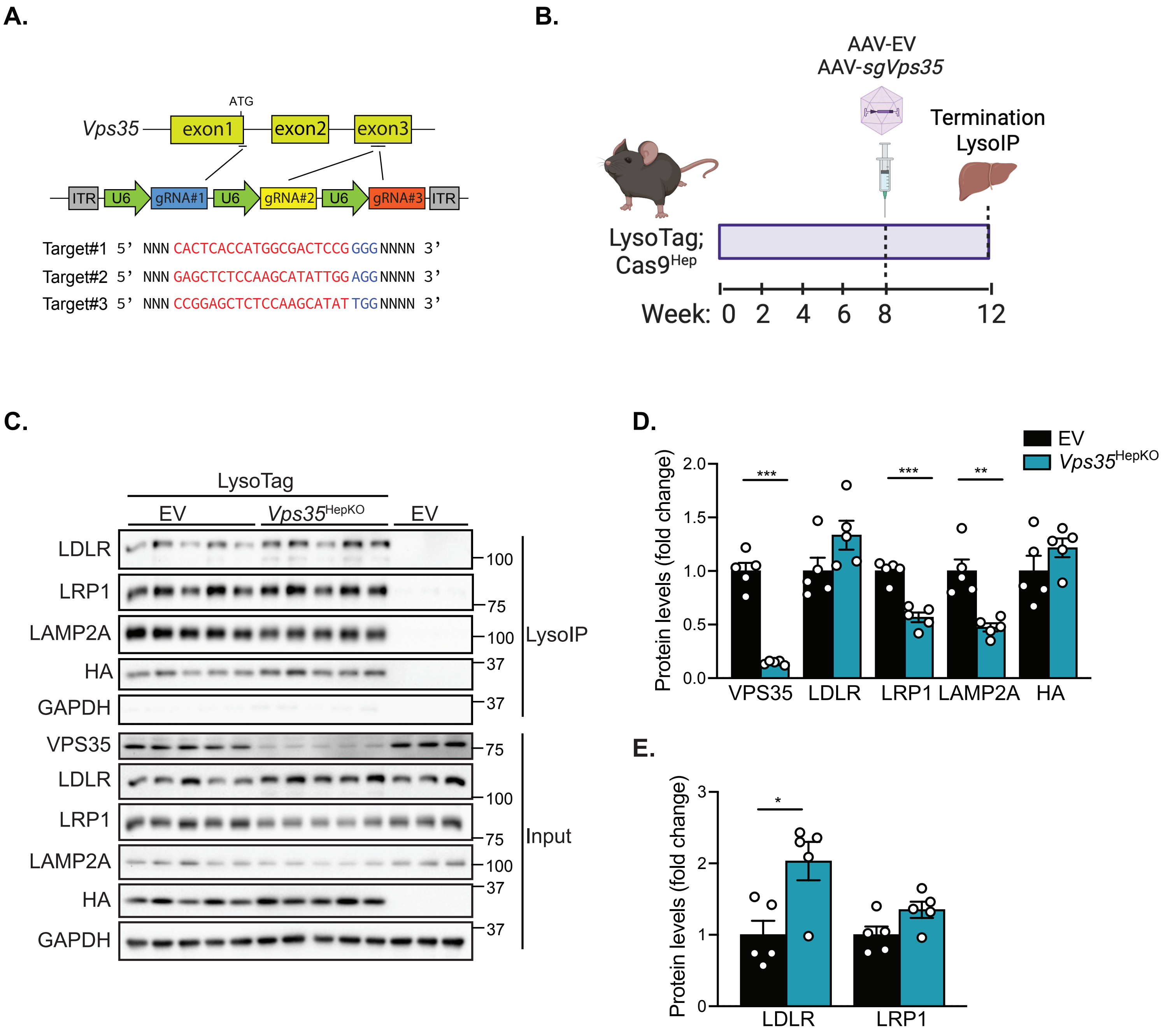

Supplemental Figure 5

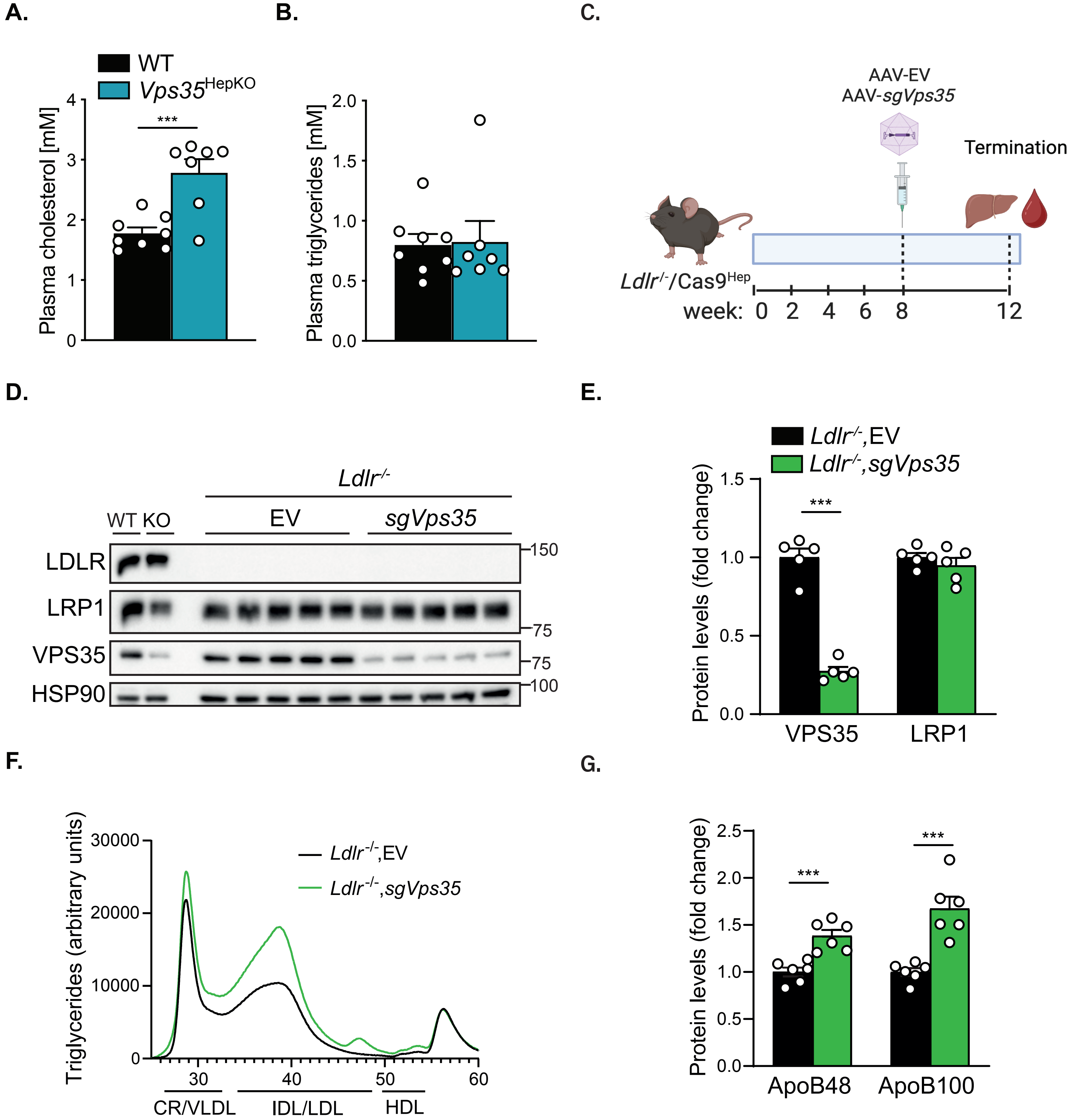

Supplemental Figure 6

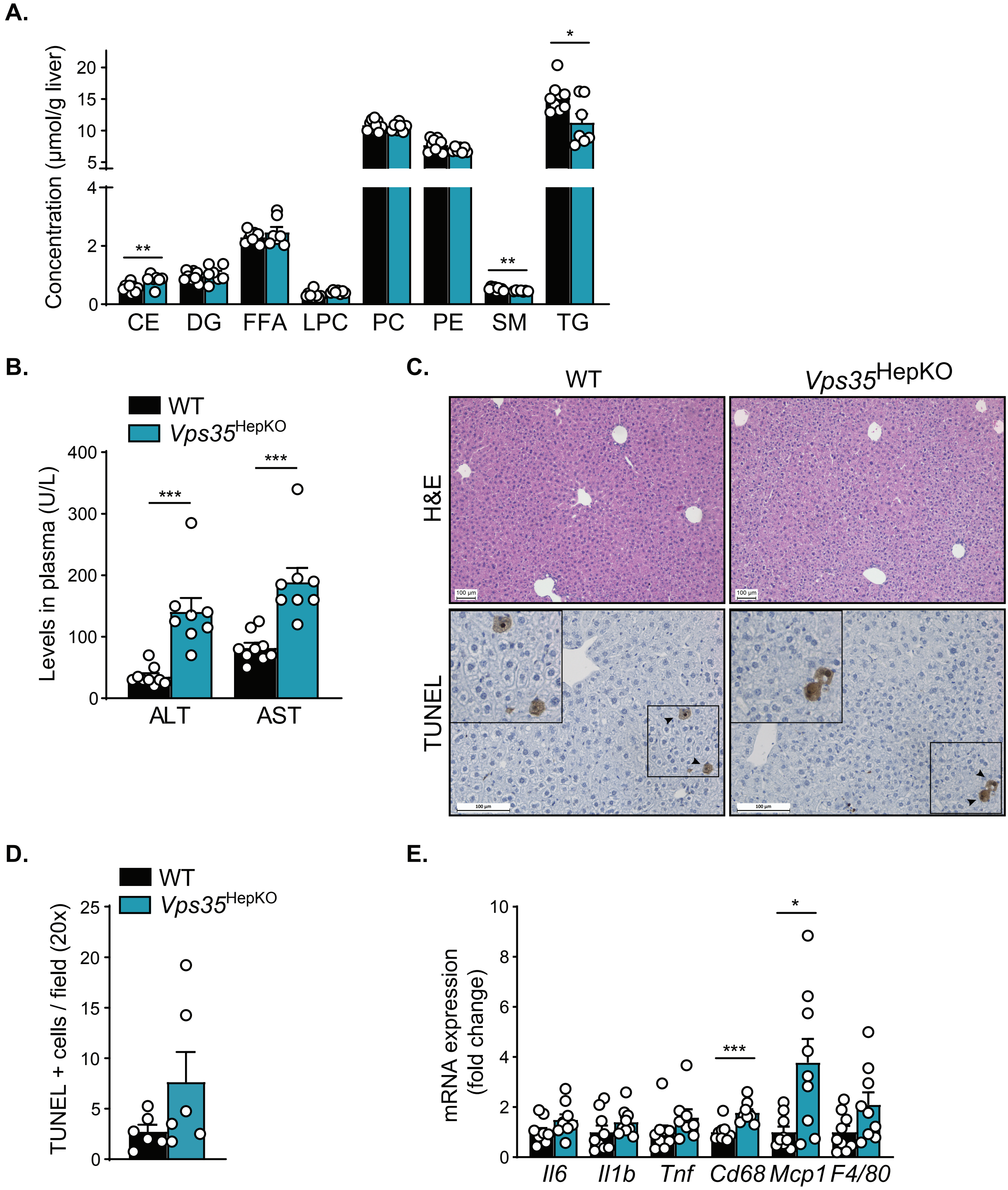

Supplemental Figure 7

A.

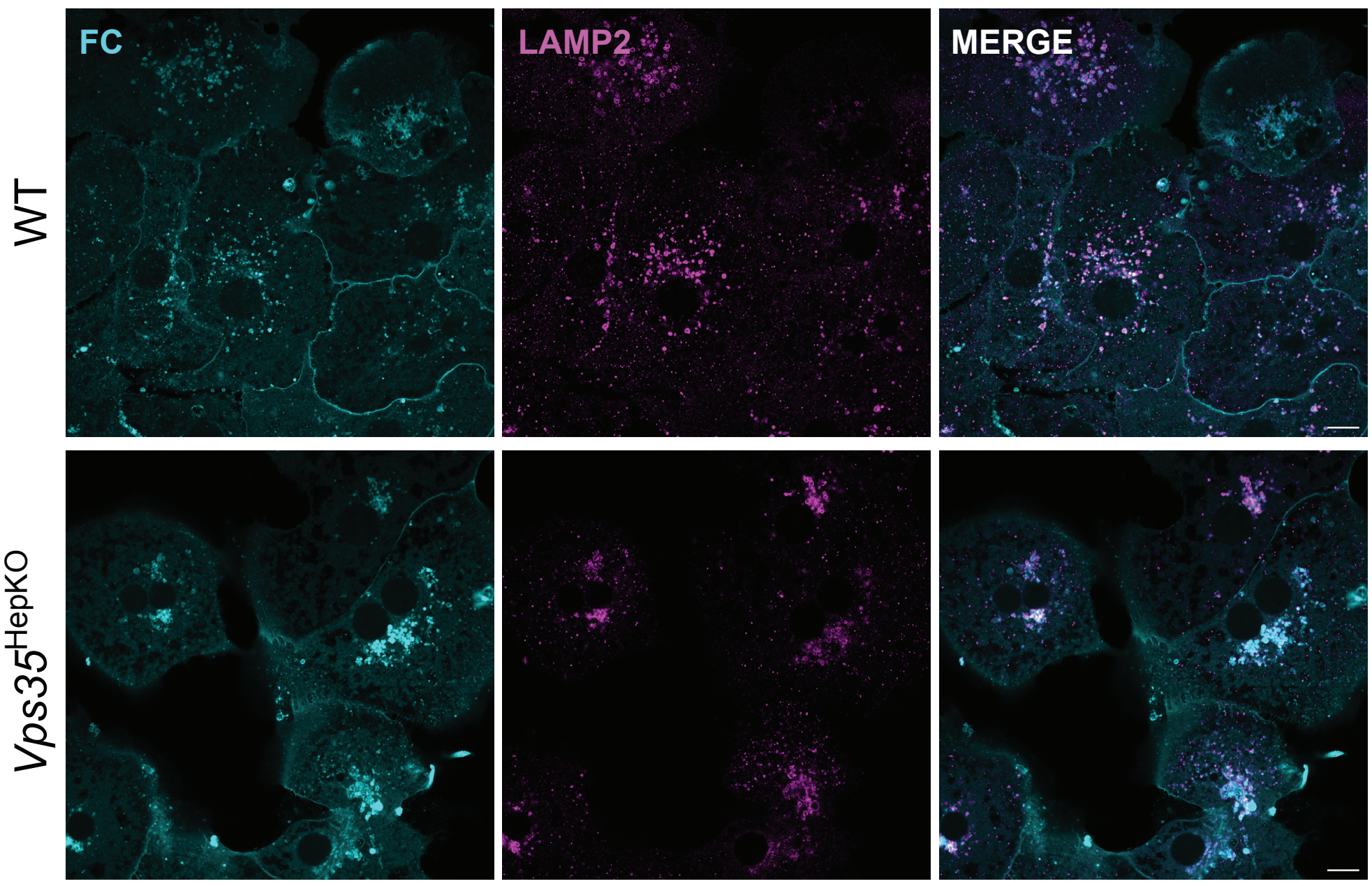

B.

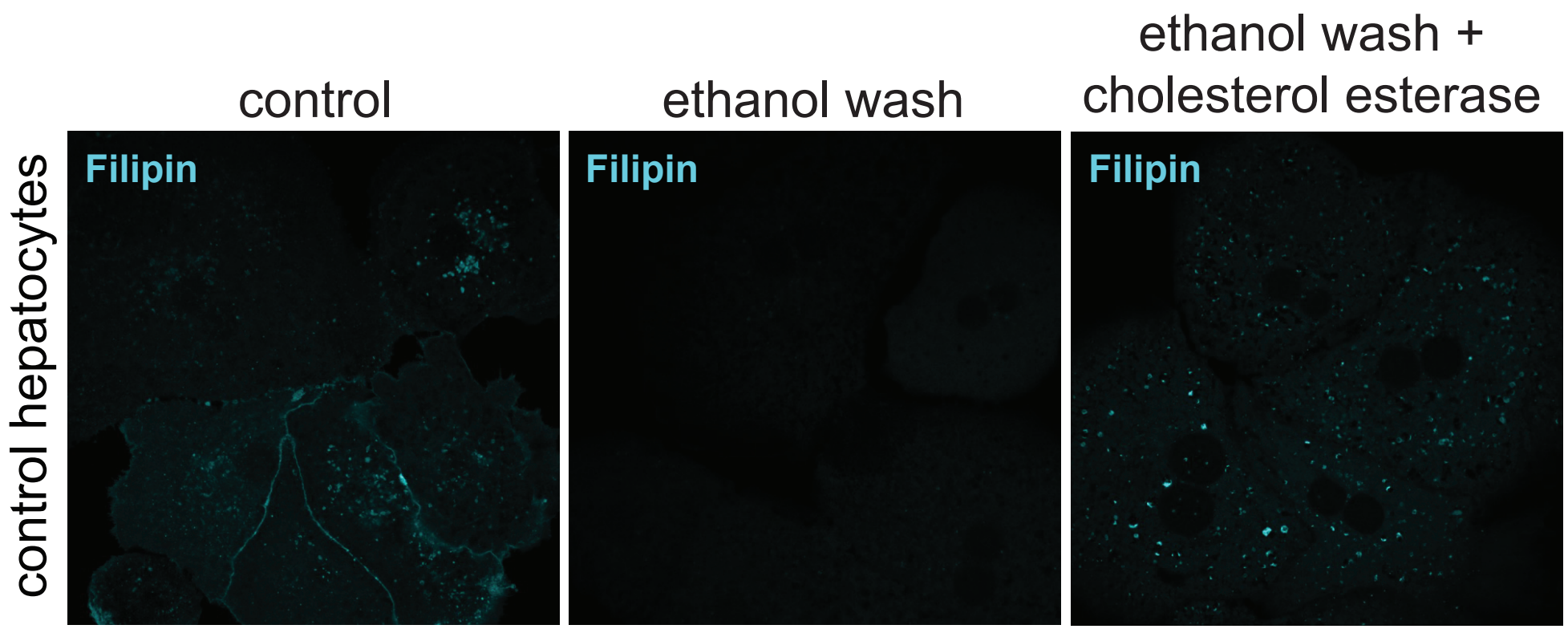

C.

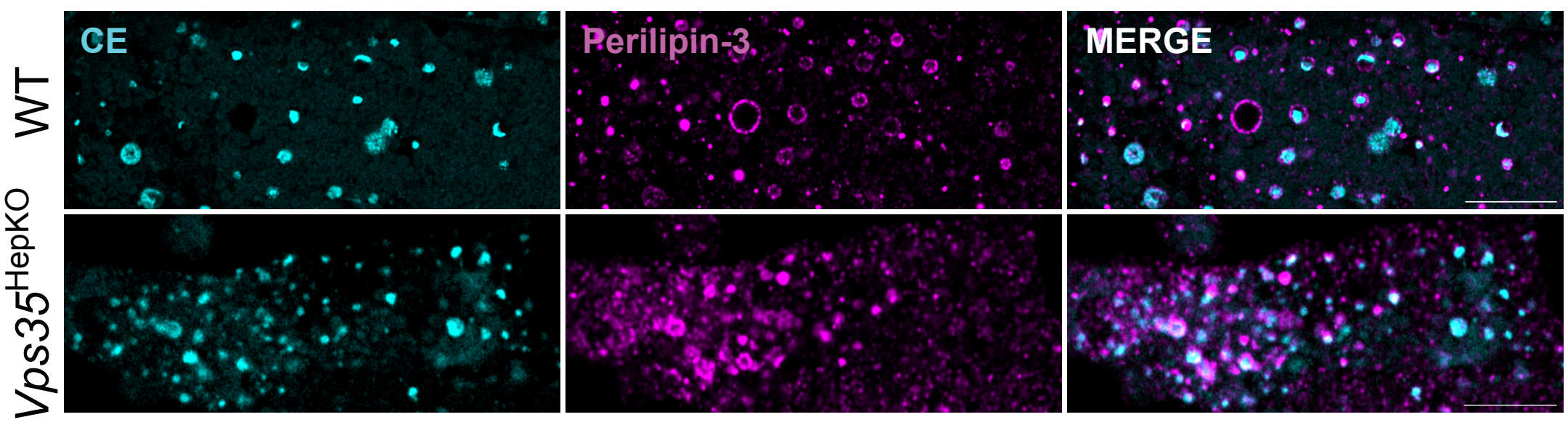

Supplemental Figure 8

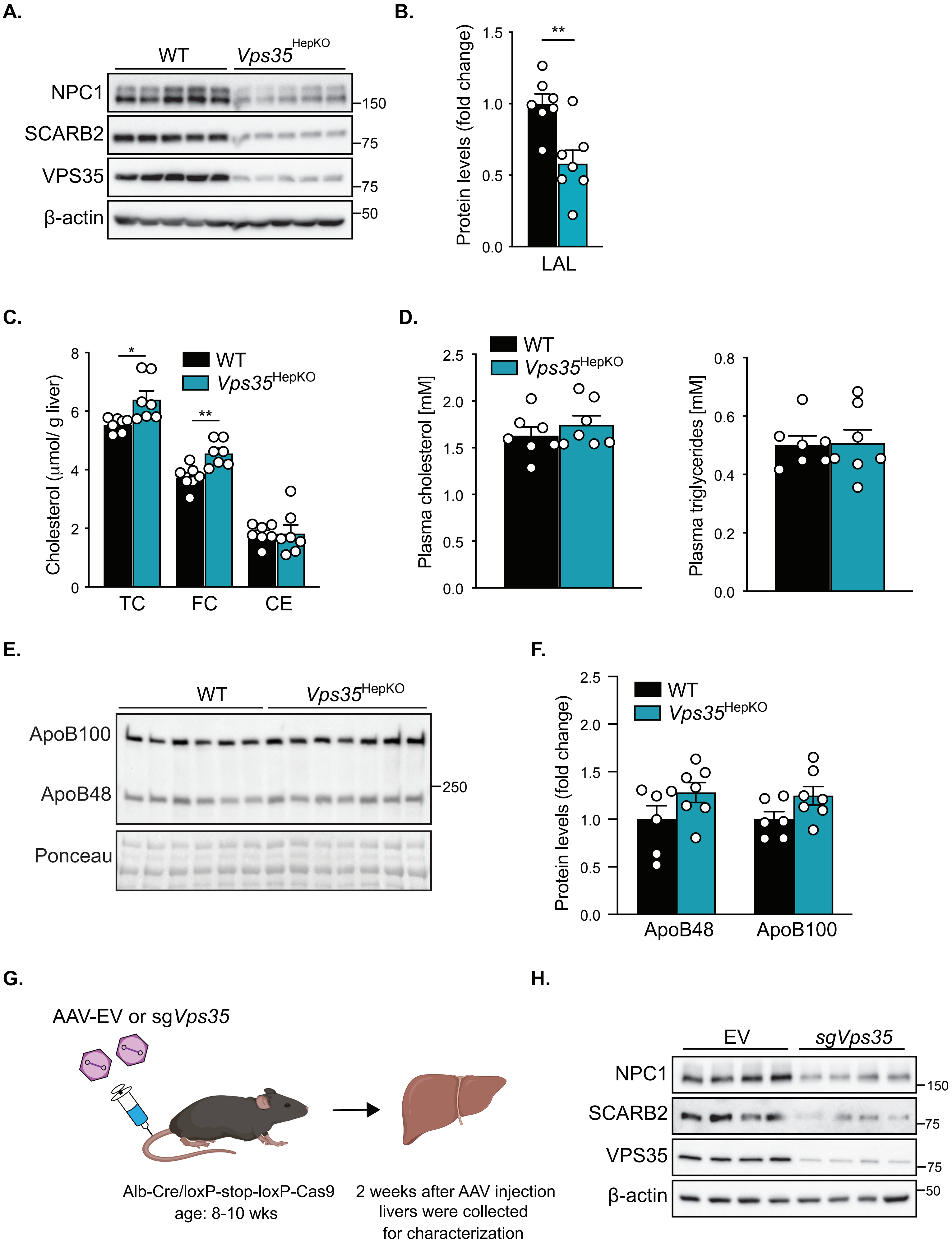

Supplemental Figure 9

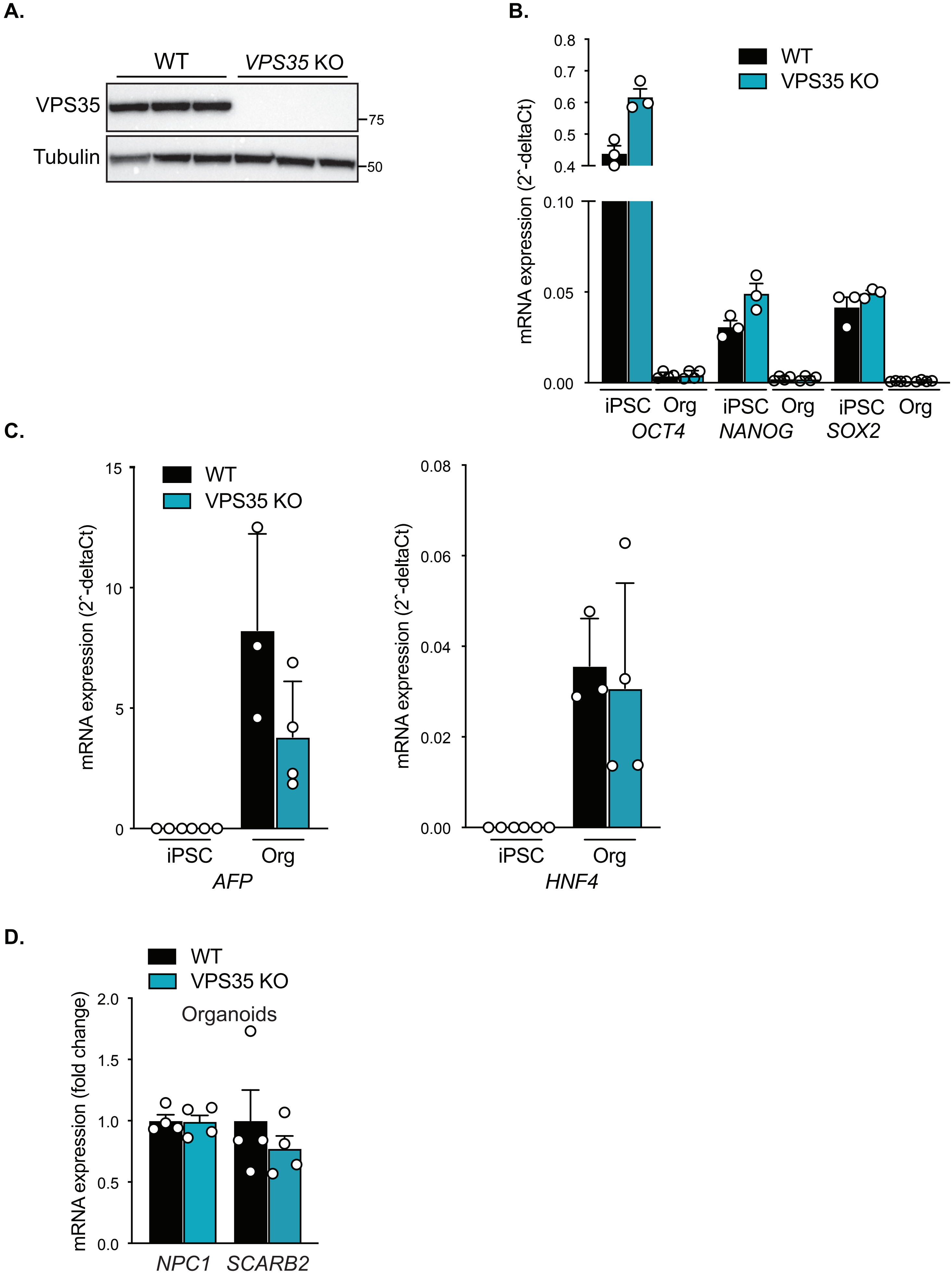

Supplemental Figure 10

A.

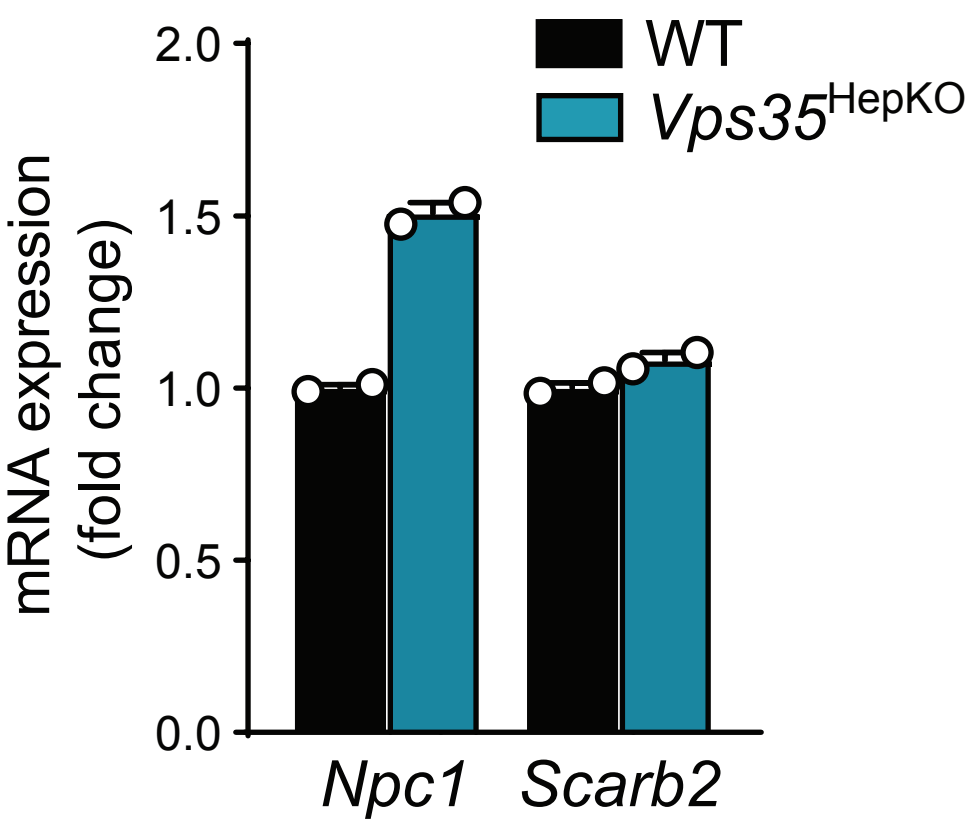

B.

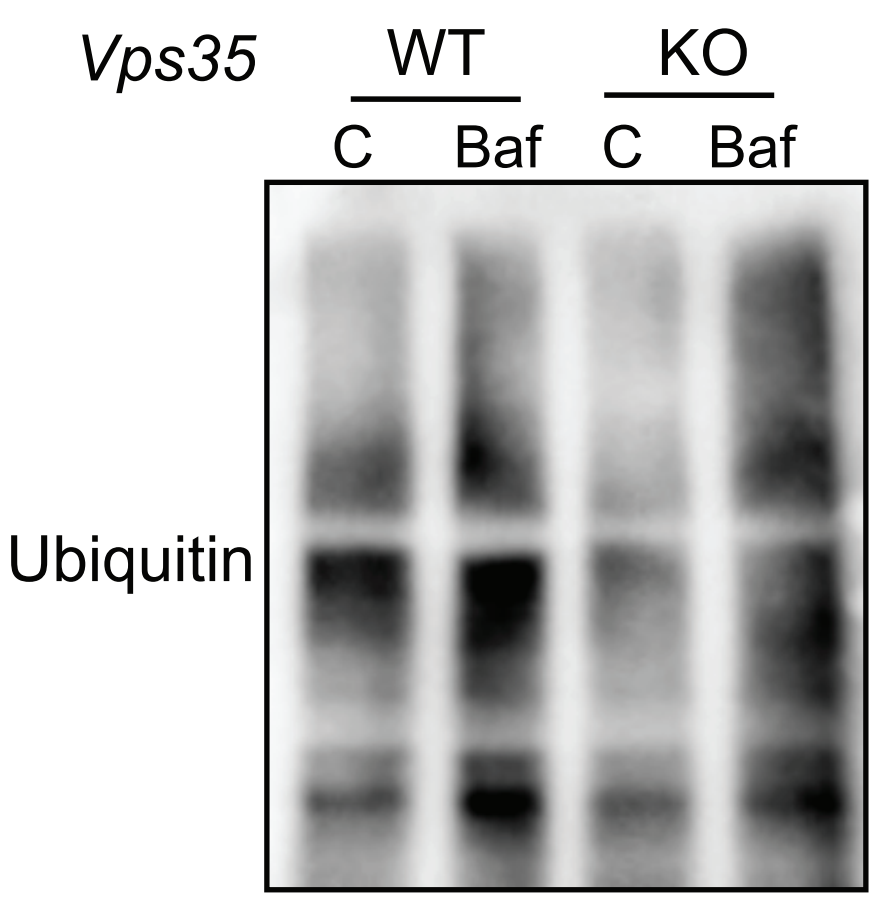

C.

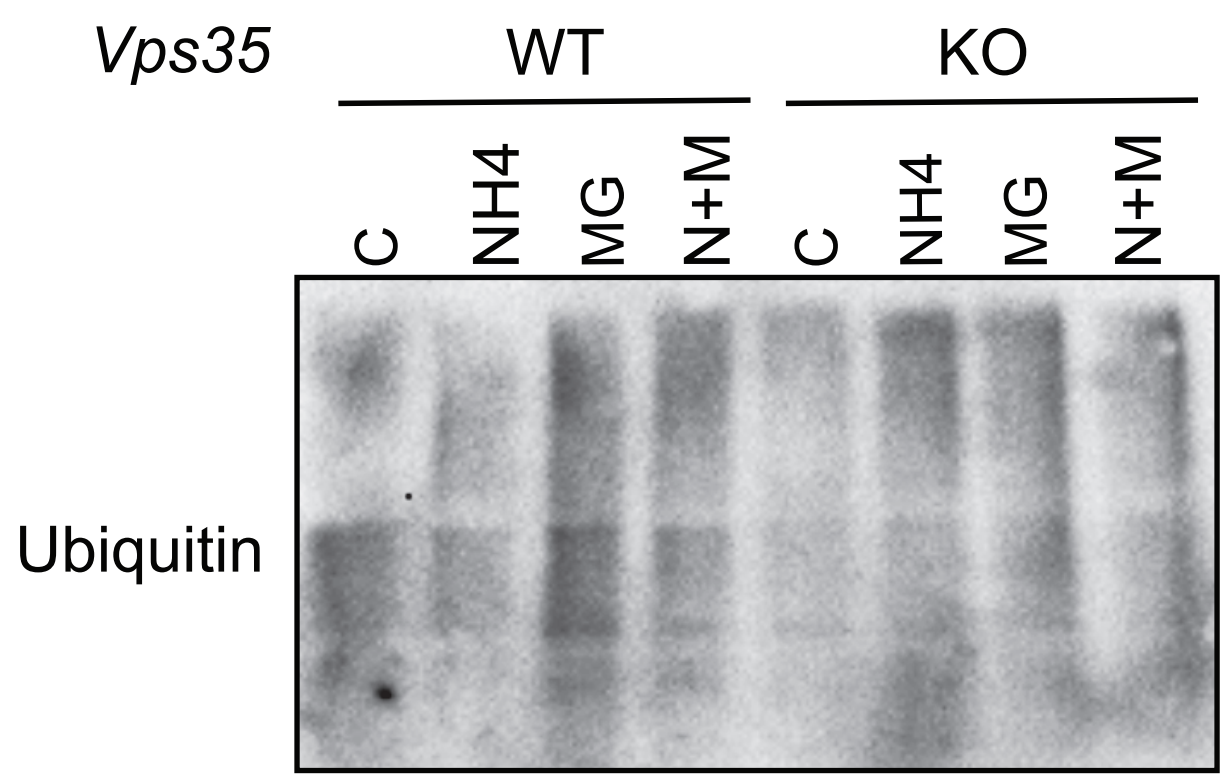

D.

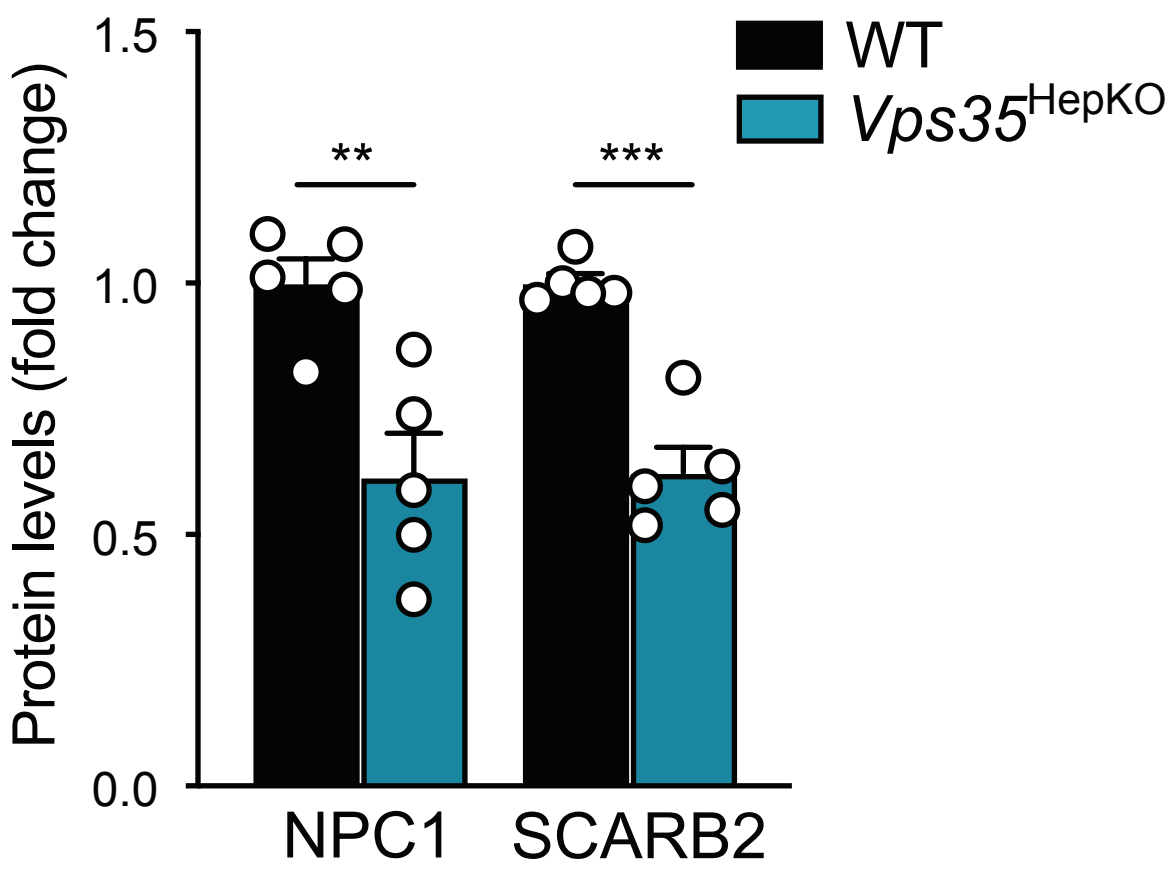

Supplemental Figure 11

A.

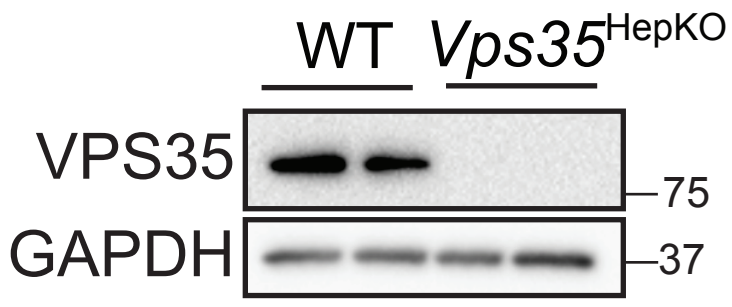

B.

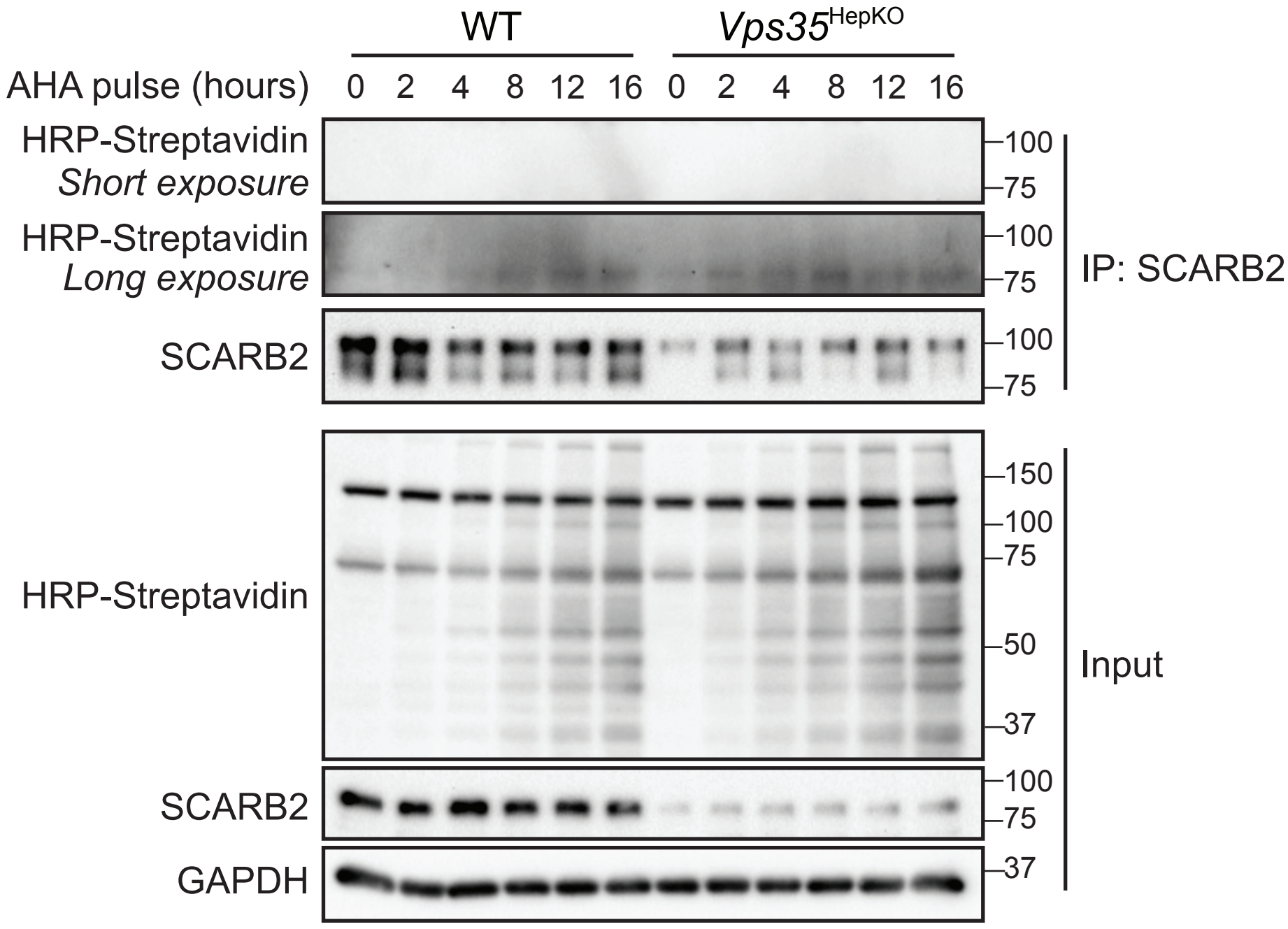

C.

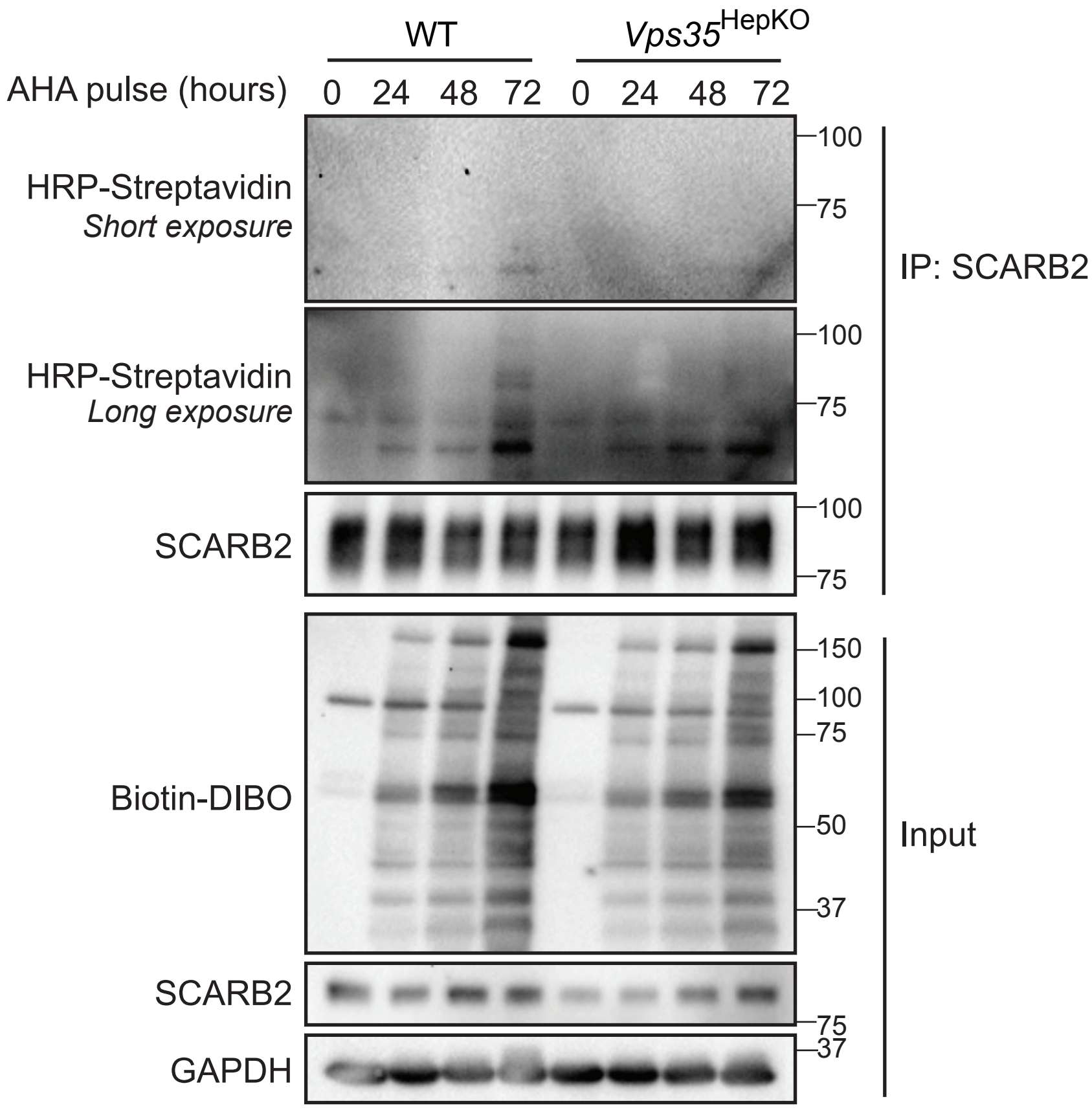

Supplement: Supplementary file 1 [file mmc2.pdf]
