# Supplementary material for: Hepatic retromer is essential for systemic cholesterol homeostasis by regulating lysosomal cholesterol handling
Source: Mol Metab. 2026 Oct 11;114:102456. doi: 10.1016/j.molmet.2026.102456 (PMC13717181; doi:10.1016/j.molmet.2026.102456)
Supplement: Supplementary file 2 [file mmc1.docx]

**Supplemental Information and Data**

**Hepatic retromer is essential for systemic cholesterol homeostasis by regulating lysosomal cholesterol handling**

Cristy R.C. Verzijl^1*^, Markus G. Barbosa^1*^, Dyonne Y. Vos^1*^, Andries H. Heida^1^, Amandine Caillaud^2^, Lauri Vanharanta^3,4^, Xiaoke Ge^5,6^, Joël J. Tissink^1^, Mirjam H. Koster^1^, Marieke Smit^1^, Nicolette Huijkman^1^, Niels J. Kloosterhuis^1^, Aurore Girardeau^2^, Zoé Begué-Racapé^2^, Vincent W. Bloks^1^, Rick Havinga^1^, Marceline M. Fuh^7^, Fulvio Reggiori^8^, Muriel Mari^8^, Ludger Scheja^7^, Folkert Kuipers^1,9^, Santiago Solé-Domènech^10^, Sander Kooijman^5,6^, Joerg Heeren^7^, Jan Freark de Boer^1,11^, Patrick C.N. Rensen^5,6^, Elina Ikonen^3,4^, Antoine Rimbert^2^, Justina C. Wolters^1^, Jan Albert Kuivenhoven^1^, Bart van de Sluis^1^

^1^Department of Pediatrics, ^9^European Research Institute for the Biology of Ageing (ERIBA) and ^11^Department of Laboratory Medicine, University of Groningen, University Medical Center Groningen, Groningen, The Netherlands; ^2^ Nantes Université, CHU Nantes, CNRS, INSERM, l’institut du thorax, F-44000 Nantes, France; ^3^Department of Anatomy and Stem Cells and Metabolism Research Program, University of Helsinki, Helsinki, Finland; ^4^Minerva Foundation Institute for Medical Research, Helsinki, Finland; ^5^Department of Medicine, Division of Endocrinology, Leiden University Medical Center, Albinusdreef 2, 2333 ZA Leiden, The Netherlands; ^6^Einthoven Laboratory for Experimental Vascular Medicine, Leiden University Medical Center, Albinusdreef 2, 2333 ZA Leiden, The Netherlands; ^7^Department of Biochemistry and Molecular Cell Biology, University Medical Center Hamburg-Eppendorf, Hamburg, Germany; ^8^Department of Biomedicine, Aarhus University, Aarhus, Denmark.^10^ Department of Biochemistry and Biophysics, Weill Cornell Medicine, New York, USA.

* Equal contribution

**Supplemental Figure Legends**

**Figure S1. Simplified model of the endosomal protein sorting complex. (A)** Upon endocytosis, receptors, like LDLR and LRP1, are recycled back to the cell surface. Our recent work demonstrates the essential role of the retriever, CCC, and WASH complexes in selective endosomal cargo sorting. However, the role of retromer in endosomal LDLR and LRP1 transport remains unclear. (**B**) The structure of the Commander complex with sub-assemblies, retriever and CCC complex, WASH complex, and retromer; subunits as C1-10 indicate COMMD proteins, W1-5 indicate WASHC proteins.

**Figure S2. Hepatic VPS35 deficiency does not affect the retriever, CCC and WASH complexes.** (**A**) Protein levels of retriever and CCC complex subunits in WT and *Vps35*^HepKO^ male livers (n=4), determined by immunoblotting. (**B**) Relative distribution of retromer, retriever, CCC, WASH subunits, LAMP1, LDLR and LRP1 in WT and *Vps35*^HepKO^ male liver homogenates determined by sucrose gradient fractionation and proteomics; presented as the percentage of total protein levels of all WT fractions.

**Figure S3. Body and liver weight are increased upon hepatic *Vps35* ablation.** (**A**)**.** Body and (**B**) liver weights of WT and *Vps35*^HepKO^ male mice at 18 weeks of age (n=9). (**C**) Relative mRNA levels of hepatic *Ldlr* and *Lrp1* in WT and *Vps35*^HepKO^ male mice (n=9). Data are presented as mean ± SEM; * p < 0.05 and *** p < 0.001.

**Figure S4. Hepatic VPS35 ablation results in increased LAMP2-positive lysosomal LDLR levels. (A, B).**  Schematic illustration of single-vector AAV (adeno-associated virus) system for targeting *Vps35* in (**B**) hepatic Cas9-expressing; LysoTag (LysoTag; Cas9^Hep^); (**A**) Target sequences are shown in red and PAM sequences in blue. (**B**) Control and AAV-sgVps35 were injected into 8-week-old LysoTag/Cas9^Hep^ mice. Four weeks after injection, the mice were sacrificed to isolate intact lysosomes from their livers. (**C**) LDLR and LRP1 levels in isolated lysosomes from control (EV) and liver-specific VPS35-deficient (*Vps35*^HepKO^) male mice, as determined by immunoblotting (**D, E**). Quantification of the indicated proteins, as determined in (**D**) the total protein levels (input), and (**E**) shows relative lysosomal levels of LDR and LRP1, corrected for LAMP2 levels. Data are presented as mean ± SEM; * p < 0.05, ** p < 0.01, *** p < 0.001.

**Figure S5. Characterization of liver-specific VPS35-deficient mice and LDLR-deficient mice lacking hepatic VPS35.** (**A**) Cholesterol and (**B**) TG levels in plasma of WT and *Vps35*^HepKO^ male mice (n=7-8) used for the LDL clearance assay. (**C**) Schematic illustration of a single-vector AAV (adeno-associated virus) system for targeting *Vps35* in hepatic Cas9-expressing, LDLR-deficient mice (*Ldlr*^-/-^;Cas9^Hep^). Control and AAV-sgVps35 were injected into 8-week-old *Ldlr^-/-^*;Cas9^Hep^ mice. Four weeks after injection, the mice were sacrificed. (**D**) Hepatic protein expression of LDLR, LRP1, and VPS35 in *Ldlr^-/-^*;Cas9^Hep^ mice and hepatic VPS35-deficient *Ldlr^-/-^*;Cas9^Hep^ male mice, determined by immunoblotting. The first two lanes are control liver homogenates from WT and hepatic VPS35-deficient mice, respectively. (**E**) Quantification of the protein levels in (**G**) (n=5). (**F**) Triglyceride levels in plasma fractionated by FPLC of LDLR-deficient and hepatic VPS35-deficient *Ldlr^-/-^*;Cas9^Hep^ male mice (n=7) (**G**) Quantification of ApoB levels in plasma of *Ldlr^-/-^*;Cas9^Hep^ mice and hepatic VPS35-deficient *Ldlr^-/-^*;Cas9^Hep^ male mice, determined by immunoblotting, shown in **Figure 2J**. Data are presented as mean ± SEM; *** p < 0.001.

**Figure S6. Hepatic loss of VPS35 does not lead to severe liver damage or liver inflammation.** (**A**) Concentrations of lipid classes in livers of WT and *Vps35*^HepKO^ male mice, determined by lipidomics analysis (n=7-9). (**B**) Plasma levels of ALT and AST (n=8-9). (**C**) Representative images of H&E and TUNEL staining of liver sections from WT and *Vps35*^HepKO^ male mice; scale bars represent 100μm. (**D**) Quantification of TUNEL^+^ cells from images shown in **B** (n=6). (**E**) Relative hepatic mRNA levels of inflammatory markers (n=9). Data are presented as mean ± SEM; * p < 0.05, ** p < 0.01 and *** p < 0.001. *Abbreviations: CE = cholesteryl ester; DG = diacylglycerol; FC = free cholesterol; FFA = free fatty acid; LPC = lysophosphatidylcholine; PC = phosphatidylcholine; PE = phosphatidylethanolamine; SM = sphingomyelin; TC = total cholesterol; TG = triacylglycerol. ALT = alanine transaminase; AST = aspartate transaminase*

**Figure S7. Free cholesterol and cholesterol esters content in control and VPS35-deficient hepatocytes. (A)** Airyscan fluorescence microscopy images of wild-type (WT) and VPS35-deficient mouse primary hepatocytes stained with filipin to detect free cholesterol (FC) and LAMP2 antibody to detect lysosomes. Scale 10 µm. Larger image of **Figure 4C**. (**B**) Control hepatocytes were stained with filipin to detect FC before and after the cells were treated with ethanol to extract FC and after ethanol extraction and incubation of the cells with cholesterol esterase. (**C**) Airyscan fluorescence microscopy images of wild-type (WT) and VPS35-deficient primary hepatocytes treated as in **Figure 4E** to detect cholesterol esters and stained with Perilipin-3 antibody to detect lipid droplets. WT scale = 10 µm, VPS35-KO scale = 6.7 µm.

**Figure S8. Protein levels of NPC1 and SCARB1 in hepatic VPS35-deficient livers of female mice and in a model in which hepatic *Vps35* was acutely ablated. (A)** Immunoblot of NPC1 and SCARB2 in WT and *Vps35*^HepKO^ female livers (n=5), age 10 wks. (**B**) Hepatic LAL levels, as determined by proteomics. (**C**) hepatic cholesterol content (*TC = total cholesterol*, *FC = free cholesterol*, *CE cholesteryl ester*), and (**D**) plasma cholesterol and triglyceride content in WT and *Vps35*^HepKO^ female livers (n=7), age 10 wks. (**E**) ApoB levels in plasma of WT and *Vps35*^HepKO^ female livers (n=6-7), age 10 wks. (**F**) Quantification of the ApoB levels shown in **F**. (G) Schematic illustration of a single-vector AAV system for targeting *Vps35* in hepatic Cas9-expressing mice. Control and AAV-sgVps35 were injected into 8-week-old Cas9^Hep^ mice. Two weeks after injection, we sacrificed the mice. (**H**) Hepatic protein expression of NPC2, SCARB2 and VPS35 in control (EV) and sg*Vps35* mice, determined by immunoblotting. Data are presented as mean ± SEM; * p < 0.05 and ** p < 0.01.

**Figure S9. Basic characterization of the human iPSCs and human iPSC-derived liver organoids.** (**A**) Protein expression of VPS35 in control WT and VPS35 knockout (KO) iPSCs, as determined by immunoblotting. (**B**) mRNA expression of several stemness and (**C**) liver organoids markers in WT and VPS35 KO iPSCs and liver organoids (WT and VPS35 knockout iPSC clones were differentiated into liver organoids, data present 3-4 independent differentiation experiments). (**D**) mRNA expression of NPC1 and SCARB2 in WT and VPS35 KO human iPSC-derived liver organoids (n=4).

**Figure S10.** **mRNA and protein levels of NPC1 and SCARB2 in WT and *Vps35*^HepKO^ hepatocytes or plasma membrane-enriched fraction, respectively.** (**A**) Relative mRNA expression of *Npc1* and *Scarb2* in primary hepatocytes isolated from WT and *Vps35*^HepKO^ mice (n=2). (**B**, **C**) The levels of ubiquitinated proteins in WT or *Vps35*^HepKO^ liver slices after 16 hours of treatment with different inhibitors as indicated, determined by immunoblotting. C = DMSO, Baf = Bafilomycin (25 nM), NH4 = ammonium chloride (2.5 mM), MG = MG132 (2.5 µM), N+M = ammonium chloride (2.5 mM) and MG132 (2.5 µM). (**D**) Hepatic NPC1 and SCARB2 protein levels in plasma membrane-enriched fractions, determined by proteomics (n=5). Data are presented as mean ± SEM with ** p < 0.01 and *** p < 0.001.

**Figure S11. Click chemistry-based pulse experiment to study newly synthesized SCARB2 proteins.** (**A**) Protein expression of VPS35 in control and VPS35-deficient primary hepatocytes, which were used for the pulse experiments. (**B, C**) Pulse experiment to label newly synthesized proteins with L-azidohomoalanine (AHA) for different hours (as indicated). Protein levels of the indicated proteins in whole-cell lysates (input) and after SCARB2 immunoprecipitation (IP). AHA-labeled proteins reacted with 10 µM Biotin-DIBO were resolved on SDS-PAGE gels, transferred to PVDF membranes, and detected by chemiluminescence using an HRP-conjugated streptavidin, or with the indicated antibodies.

**Supplemental Tables**

**Supplemental Table 1.** Sequence of the sgRNAs used in the current study.

| Gene |  | Sequence (5’-3’) |
| --- | --- | --- |
| *Vps35* | sgRNA #1 | CACTCACCATGGCGACTCCG |
|  | sgRNA #2 | GAGCTCTCCAAGCATATTGG |
|  | sgRNA #3 | CCGGAGCTCTCCAAGCATAT |
| *VPS35* | sgRNA #1 | TACGAACTTGTACAGTATGC |
|  | sgRNA #2 | GGTGTGCAACATCCCTTGAG |

**Supplemental Table 2.** Primer sequences used for qRT-PCR and PCR analysis.

| Gene | Forward primer (5’-3’) | Reverse primer (5’-3’) |
| --- | --- | --- |
| *Cd68* | TGACCTGCTCTCTCTAAGGCTACA | TCACGGTTGCAAGAGAAACATG |
| *F4/80* | TGTGTCGTGCTGTTCAGAACC | AGGAATCCCGCAATGATGG |
| *Il1b* | TGCAGCTGGAGAGTGTGG | TGCTTGTGAGGTGCTGATG |
| *Il6* | CTGCAAGAGACTTCCATCCAGTT | AGGGAAGGCCGTGGTTGT |
| *Lipa* | TGTTCGTTTTCACCATTGGGA | CGCATGATTATCTCGGTCACA |
| *Ldlr* | TCCAATCAATTCAGCTGTGG | GAGCCATCTAGGCAATCTCG |
| *Lrp1* | GACCAGGTGTTGGACACAGATG | AGTCGTTGTCTCCGTCACACTTC |
| *Mcp1* | GCTGGAGAGCTACAAGAGGATCA | ACAGACCTCTCTCTTGAGCTTGGT |
| *Npc1* | TGTTTGGTATGGAGAGTGTGGA | GTCACAGCAGAGACTGACATTG |
| *Npc2* | AGGACTGCGGCTCTAAGGT | AGGCTCAGGAATAGGGAAGGG |
| *Ppia* | TTCCTCCTTTCACAGAATTATTCCA | CCGCCAGTGCCATTATGG |
| *Scarb2* | AGAAGGCGGTAGACCAGAC | GTAGGGGGATTTCTCCTTGGA |
| *Tnf* | GTAGCCCACGTCGTAGCAAAC | AGTTGGTTGTCTTTGAGATCCATG |
| *VPS35* | TAAATGGCTGACTGGGTGGA | GTGTTGTCGGTGCATCTCAA |
| *GAPDH* | AATCCCATCACCATCTTCCA | TGGACTCCACGACGTACTCA |
| *OCT4* | TGGGTGGAGGAAGCTGACAACAAT | TTCGGGCACTGCAGGAACAAATTC |
| *NANOG* | ATAGCAATGGTGTGACGCAGAAGG | CTGGTTGCTCCACATTGGAAGGTT |
| *SOX2* | CCTACTCGCAGCAGGGCACC | CTCGGCGCCGGGGAGATACA |
| *VPS35 exon1-2* | TGCCTACAACACAGCAGTCC | AAGTCCGGAGTTCACCAAGC |
| *VPS35 exon3-4* | CAGGCTGTGAAGGTCCAGTC | GTCCGGAGTTCACCAAGCATA |
| *ALB* | TTTATGCCCCGGAACTCCTTT | AGTCTCTGTTTGGCAGACGAA |
| *HNF4* | CACGGGCAAACACTACGG | TTGACCTTCGAGTGCTGCTCC |
| *AFP* | AGTGAGGACAAACTATTGGCCT | ACACCAGGGTTTACTGGAGTC |
| *SCARB1* | TGGGGATGCCTTCAAACAC | TTGAACTTCTGGGCAAATG |
| *LAL* | GAGAGCGGCCCGGCA | TGCAGGGTCCAGAGAACCAA |
| *NPC1* | AGCCAGTAATGTCACCGAAAC | CCGAGGTTGAAGATAGTGTCG |

**Supplemental Table 3.** Antibodies used for immunoblot analysis.

| Target | Company | Catalog # | Dilution |
| --- | --- | --- | --- |
| β-actin | Sigma | A5441 | 1:1000 |
| ApoB | EMD Millipore | 178467 | 1:1000 |
| Tubulin | Sigma | T9026 | 1:500 |
| CDCC22 | Proteintech | 16636-1-AP | 1:1000 |
| CDCC93 | Proteintech | 20861-1-AP | 1:1000 |
| COMMD1 | Proteintech | 11938-1-AP | 1:1000 |
| HSP90 | Cell Signaling | 4874 | 1:1000 |
| LAMP1 | BioLegend | 121602 | 1:1000 |
| LDLR | Abnova | PAB8804 | 1:1000 |
| LRP1 | Abcam | ab92544 | 1:1000 |
| NPC1 | Invitrogen | PA1-16817 | 1:1000 |
| SCARB2 | Cell Signaling | 27960 | 1:1000 |
| Vinculin | Cell Signaling | 4650 | 1:1000 |
| VPS26 | Abcam | ab23892 | 1:1000 |
| VPS29 | Abcam | ab98929 | 1:1000 |
| VPS35 | Abcam | ab10099 | 1:1000 |
| VPS35L | Thermofisher | PA5-28553 | 1:1000 |
| goat anti-mouse IgG (H+L)-HRP conjugate | Bio-Rad | 170-6516 | 1:10000 |
| goat anti-rabbit IgG (H+L)-HRP conjugate | Bio-Rad | 170-6515 | 1:10000 |
